# Hepmarc: A 96 week randomised controlled feasibility trial of add-on maraviroc in people with HIV and non-alcoholic fatty liver disease

**DOI:** 10.1371/journal.pone.0288598

**Published:** 2023-07-14

**Authors:** Daniel Bradshaw, Iga Abramowicz, Stephen Bremner, Sumita Verma, Yvonne Gilleece, Sarah Kirk, Mark Nelson, Rosalie Housman, Helena Miras, Chloe Orkin, Ashini Fox, Michael Curnock, Louise Jennings, Mark Gompels, Emily Clarke, Rachel Robinson, Pauline Lambert, David Chadwick, Nicky Perry

**Affiliations:** 1 The Lawson Unit, University Hospitals Sussex NHS Foundation Trust, Brighton, United Kingdom; 2 Brighton and Sussex Clinical Trials Unit, University of Sussex, Brighton, United Kingdom; 3 Brighton and Sussex Medical School, University of Sussex, Brighton, United Kingdom; 4 Department of HIV and Sexual Health, Chelsea and Westminster Hospital NHS Foundation Trust, London, United Kingdom; 5 Grahame Hayton Unit, Barts Health NHS Trust, London, United Kingdom; 6 Department of Genitourinary Medicine and HIV, Nottingham University Hospitals NHS Trust, Nottingham, United Kingdom; 7 Department of HIV, North Bristol NHS Trust, Bristol, United Kingdom; 8 Department of Genitourinary Medicine and HIV, Liverpool University Hospitals NHS Foundation Trust, Liverpool, United Kingdom; 9 Department of Infectious Diseases, South Tees Hospitals NHS Foundation Trust, Middlesbrough, United Kingdom; Imperial College London, UNITED KINGDOM

## Abstract

**Objectives:**

Maraviroc may reduce hepatic inflammation in people with HIV and non-alcoholic fatty liver disease (HIV-NAFLD) through CCR5-receptor antagonism, which warrants further exploration.

**Methods:**

We performed an open-label 96-week randomised-controlled feasibility trial of maraviroc plus optimised background therapy (OBT) versus OBT alone, in a 1:1 ratio, for people with virologically-suppressed HIV-1 and NAFLD without cirrhosis. Dosing followed recommendations for HIV therapy in the Summary of Product Characteristics for maraviroc. The primary outcomes were safety, recruitment and retention rates, adherence and data completeness. Secondary outcomes included the change in Fibroscan-assessed liver stiffness measurements (LSM), controlled attenuation parameter (CAP) and Enhanced Liver Fibrosis (ELF) scores.

**Results:**

Fifty-three participants (53/60, 88% of target) were recruited; 23 received maraviroc plus OBT; 89% were male; 19% had type 2 diabetes mellitus. The median baseline LSM, CAP & ELF scores were 6.2 (IQR 4.6–7.8) kPa, 325 (IQR 279–351) dB/m and 9.1 (IQR 8.6–9.6) respectively. Primary outcomes: all individuals eligible after screening were randomised; there was 92% (SD 6.6%) adherence to maraviroc [target >90%]; 83% (95%CI 70%-92%) participant retention [target >65%]; 5.5% of data were missing [target <20%]. There were noo Serious Adverse Reactions; mild-moderate intensity Adverse Reactions were reported by five participants (5/23, 22% (95%CI 5%-49%)) [target <10%]. All Adverse Reactions resolved. Secondary outcomes: no important differences were seen by treatment group for the change from baseline in LSM, CAP or ELF scores

**Conclusions:**

This feasibility study provides preliminary evidence of maraviroc safety amongst people with HIV-NAFLD, and acceptable recruitment, retention, and adherence rates. These data support a definitive randomised-controlled trial assessing maraviroc impact on hepatic steatosis and fibrosis.

**Trial registration:**

**Clinical trial registry:** ISCRTN, registration number 31461655.

## Introduction

Non-alcoholic fatty liver disease (NAFLD), defined as fat accumulation in ≥5% of hepatocytes without a secondary cause, [1] may progress to steatohepatitis (NASH), fibrosis, cirrhosis and hepatocellular carcinoma. NAFLD represents one of the commonest causes of liver disease in people with HIV (PWH), with a prevalence of 35% in a systematic review, but varying from 13% to 73% [2–4]. Progression to NASH and hepatic fibrosis may be more likely in PWH with NAFLD (HIV-NAFLD) than in HIV-negative individuals with NAFLD (primary NAFLD) [5]. In a meta-analysis, metabolic abnormalities were risk factors for HIV-NAFLD, including type 2 diabetes mellitus, hypertension, and elevated body mass index (BMI) [2]. HIV-related factors, such as metabolic effects of viral proteins, immune activation, gut microbial translocation, and antiretrovirals, may also play a role, although their contribution is not well understood [4, 6].

Lifestyle changes, including physical activity and dietary modification, are the mainstay of therapy [7] for HIV-NAFLD, but are difficult to achieve [8]. Pharmacological therapies are urgently required but few trials have investigated potential candidates, especially in PWH [1]. In a randomised controlled trial (RCT), the growth hormone-releasing hormone analogue, tesamorelin, reduced hepatic fat fraction in HIV-NAFLD; however, it is given by subcutaneous injection, limiting its use [9]. In a small, single-arm trial, oral vitamin E showed efficacy in HIV-NAFLD, although there are concerns over long-term safety [1, 10].

Chemokine (C-C motif) ligand 5 (CCL5)/ Regulated upon Activation, Normal T cell Expressed and Secreted (RANTES), the ligand for C-C chemokine receptor type 5 (CCR5), plays a key role in hepatic inflammation. CCR5 mediates intrahepatic immune cell interactions which promote activation and migration of Kupffer cells and hepatic stellate cells; these in turn promote inflammation and fibrosis [11, 12]. Antagonism of this pathway could therefore reduce fibrosis progression [13, 14].

The CCR5 receptor antagonist, maraviroc (MVC), is licensed for HIV-1 treatment as part of combination antiretroviral therapy (cART) in therapy-naïve and -experienced individuals, where the infecting strain is CCR5 tropic [15, 16]. MVC inhibits HIV-1 gp120 binding to the CCR5 co-receptor, preventing virus entry into the cell. Its antagonism of CCL5-CCR5-mediated interactions raises the possibility of additional anti-inflammatory effects. Data from *in vitro* studies, mouse models, and retrospective HIV/HCV and HIV-NAFLD cohorts, suggest a reduction in hepatic fibrosis progression [17–20]. In an RCT, cenicriviroc, a CCR2 and CCR5 antagonist, reduced hepatic fibrosis in primary NAFLD [21].

MVC therefore represents a potential treatment for HIV-NAFLD. However, dosing is usually twice daily, unlike currently recommended antiretrovirals. We therefore aimed to conduct a RCT to evaluate the safety, acceptability and feasibility of MVC add-on therapy to cART in HIV-NAFLD in preparation for conducting a larger RCT in the future. The RCT was successfully completed and findings are presented below.

## Materials and methods

### Study design and participants

This was a phase IV, open-label, dual, parallel arm, randomised, multi-centre, feasibility trial comparing MVC plus optimised background therapy (MVC+OBT) versus OBT alone in HIV-NAFLD over 96 weeks. The sites were hospitals in London (Barts, Chelsea and Westminster), the Midlands (Nottingham), North (Liverpool, South Tees), South (Brighton) and West (Bristol) of England.

Potentially eligible individuals were identified by members of the direct care team through review of clinic databases, pre-identification of those attending routine consultations and review of medical notes during follow-up. Anonymised information collected on individuals contacted but not randomised were age, gender, ethnicity, reason for lack of eligibility or if they were potentially eligible but declined.

Detailed methodology has been presented previously [22]. In brief, inclusion criteria included: provision of written, informed consent, ≥18 years of age, HIV-1 viral load (VL) <50 copies/ml for ≥6 months, evidence of NAFLD including hepatic steatosis on imaging (either ultrasound, CT or MRI of the liver) or liver biopsy, performed within 6 months of screening. Exclusion criteria included: severe cardiovascular disease (including known angina or history of myocardial infarction), postural hypotension, prior MVC exposure, other causes of liver disease including viral, alcohol-associated, haemochromatosis, Wilson’s disease, alpha-1 antitrypsin deficiency, drug-induced, autoimmune; cirrhosis (defined by histology, imaging, or liver stiffness (LSM) >13kPa); ALT or AST >205 IU/L (five times the upper limit of normal [23]); severe renal insufficiency (defined as creatinine clearance <30mL/min); HIV-2; MVC allergy or intolerance; co-medications contraindicated with MVC; pregnancy; breastfeeding.

### Randomisation

Participants were randomised 1:1 in accordance with a computer-generated randomization schedule using Sealed Envelope [24] to receive MVC+OBT versus OBT. Randomisation was stratified according to: (1) current or past history of ≥6 months’ exposure to a protease inhibitor (PI) versus no current and <6 months prior exposure (2) BMI ≥25 versus <25 (3) diabetes mellitus status (4) current exposure to lipid-lowering agents. This was due to reported positive associations for (1–3) with hepatic fibrosis progression [2, 25] in HIV-NAFLD and a negative association for (4) with fibrosis progression in primary NAFLD [26, 27]. In a cohort of 300 patients with HIV monitored with transient elastography for NAFLD development, current PI use was independently predictive of significant liver fibrosis (adjusted OR 3.96, 95% CI 1.64–9.54) [25]. In a systematic review of studies enrolling patients with HIV-NAFLD, elevated BMI and fasting glucose were independently associated with increased risk of significant liver fibrosis (Mean Difference (MD) 1.38, 95% CI 0.04–2.71, p = 0.004 and MD 0.80, 95% CI 0.47–1.13, p<0.00001 respectively) [2]. In primary NAFLD, current lipid lowering agent use was associated with protection from steatosis, NASH and fibrosis stages F2-F4 in a dose dependent manner (adjusted p<0.05 for all) [26]. As this was a feasibility study, the study was unblinded, with a plan for blinding within a larger RCT, should feasibility be demonstrated.

### Procedures

MVC was prescribed twice daily at the licensed dose for HIV-1 therapy, adjusted according to co-medications according to the Summary of Product Characteristics. Participants attended every 24 weeks for 96 weeks plus a week 4 safety visit for those receiving maraviroc. All participants received advice on lifestyle and dietary modifications as standard of care interventions for HIV-NAFLD. Supplementary Table S1 in S1 File details the schedule of events.

In brief, the screening visit included history-taking, physical examination, height and weight measurement, laboratory investigations for eligibility, ECG, LSM and Controlled Attenuation Parameter (CAP) score measured by Fibroscan, and hepatic ultrasound scan. Fibroscan readings, HbA1c, CD4 T-lymphocyte count, and VL from screening were used as the baseline measures. This visit was performed by the site investigator or sub-investigator, who also confirmed eligibility. The baseline assessment included diet and exercise history, fasting bloods for glucose and lipids, Enhanced Liver Fibrosis (ELF) score and proviral DNA co-receptor tropism. There was an optional CT scan liver and spleen, and this was only offered at one site. Participants additionally completed questionnaires to evaluate quality of life (QoL): chronic liver disease questionnaire for NAFLD (CLDQ-NAFLD) [28], 36-Item Short Form Survey (SF-36) [29] and Work Productivity and Activity Impairment: Specific Health Problem (WPAI:SHP) [30].

Assessments performed each 24 weeks were review of symptoms and Adverse Events (AE), examination, weight, VL, full blood count, routine serum chemistries and urinalysis. Additional assessments performed every 48 weeks were diet and exercise history, questionnaire completion, CD4 count, fasting glucose and lipids, HbA1c, ELF score, Fibroscan. An optional CT scan of liver and spleen was performed at 96 weeks. Self-reported adherence noted on diary cards and pill counts by the pharmacist of returned MVC doses were recorded every visit. AE severity and laboratory abnormalities was assessed using the National Institute of Allergy and Infectious Diseases Division of AIDS toxicity grading scale [31]. Laboratory analyses and imaging were performed at site.

The first participant was enrolled on 24/07/18 and the final participant completed the last study assessment on 08/11/21.

### Outcome measures

The primary objective was to assess the safety, feasibility and acceptability of adding MVC to OBT in HIV-NAFLD. This was assessed through the following outcome measures and minimum target values to indicate feasibility: (1) proportion of eligible individuals who were recruited [target >50%] (2) monthly recruitment rate [>2 individuals per month] (3) retention rate [>65%] (4) proportion of participants with missing data [<80%] (5) proportion of participants with Adverse Reactions (AR) [<10%], which represents the rate observed for a potential alternative agent for treatment of HIV-NAFLD [32] (6) self-reported adherence to MVC [>90%].

Secondary outcome measures were: (1) ELF score (2) LSM (3) CAP (4) fasting lipids (5) fasting glucose (6) HbA1c (7) ALT (8) BMI (9) waist circumference (10) CD4 count (11) VL (12) change in the % with a CT liver: spleen attenuation ratio of <1.0 (13) questionnaire-assessed QoL.

### Analyses

#### Sample size

This was a pilot study evaluating feasibility, with the intention that results would be used to estimate the variability of the treatment effect of MVC on the ELF score. This in turn would inform the sample size calculation for an RCT to evaluate drug efficacy. We based our sample size justification for this pilot study on change in ELF score. In a previous longitudinal study in people with chronic liver disease, a unit increase of 1 in the ELF score was associated with a 2.5-fold increased risk of a liver-related event, adjusted for age and stage of fibrosis. A unit increase of 1 was therefore considered a clinically important entity [33]. Assuming the standard deviation (SD) of the ELF score is 1.12 [33], with 20 participants per group for the analysis, a difference in ELF of 1 point could be estimated with a 95% confidence interval (CI) from 0.3 to 1.7. Assuming an attrition rate of 33%, as reported in previous RCTs evaluating MVC [34], there was a target of 30 participants per group.

#### Statistical analyses

Reasons for screen failures are summarised in the CONSORT flowchart, according to the 2010 Statement extension to pilot and feasibility studies [35]. Baseline demographic and clinical characteristics are presented by treatment group and overall. Descriptive statistical analyses were performed. Continuous variables were described by their medians and interquartile ranges, and categorical variables by the frequency and percentage in each category.

Primary outcome measures are presented with 95% CI for the following measures, at 48 and 96 weeks: participant retention, the proportion of individuals for whom data are missing, the proportion of individuals with ARs and the level of self-reported adherence to MVC. The latter was calculated by (a) the proportion of study medication dispensed that was returned at the final study visit (b) the mean number of doses taken that were prescribed. The proportion of eligible individuals approached who were successfully recruited with 95% CI, and the monthly recruitment rate, are also presented.

For each secondary outcome, we estimated the difference in means between groups from baseline to 48 and to 96 weeks and the 95% CI around these differences [36]. A t-method, using 10,000 bootstrapped samples due to non-normality of data, was used for continuous variables and exact 95% binomial CIs for categorical variables [37]. Analyses followed intention-to-treat principles, using available data; no imputation of missing data was conducted. All analyses were performed using Stata version 17.0 [38].

### Ethics

This study was approved by the London Dulwich Research Ethics Committee (Reference 17/LO/2093). The authors did not have access to information that could identify individual participants during or after data collection. Study data will be made available on reasonable request to the Brighton & Sussex Clinical Trials Unit. The trial was registered on the ISRCTN Registry, registration number 31461655

## Results

### Participant flow

**Fig 1:**
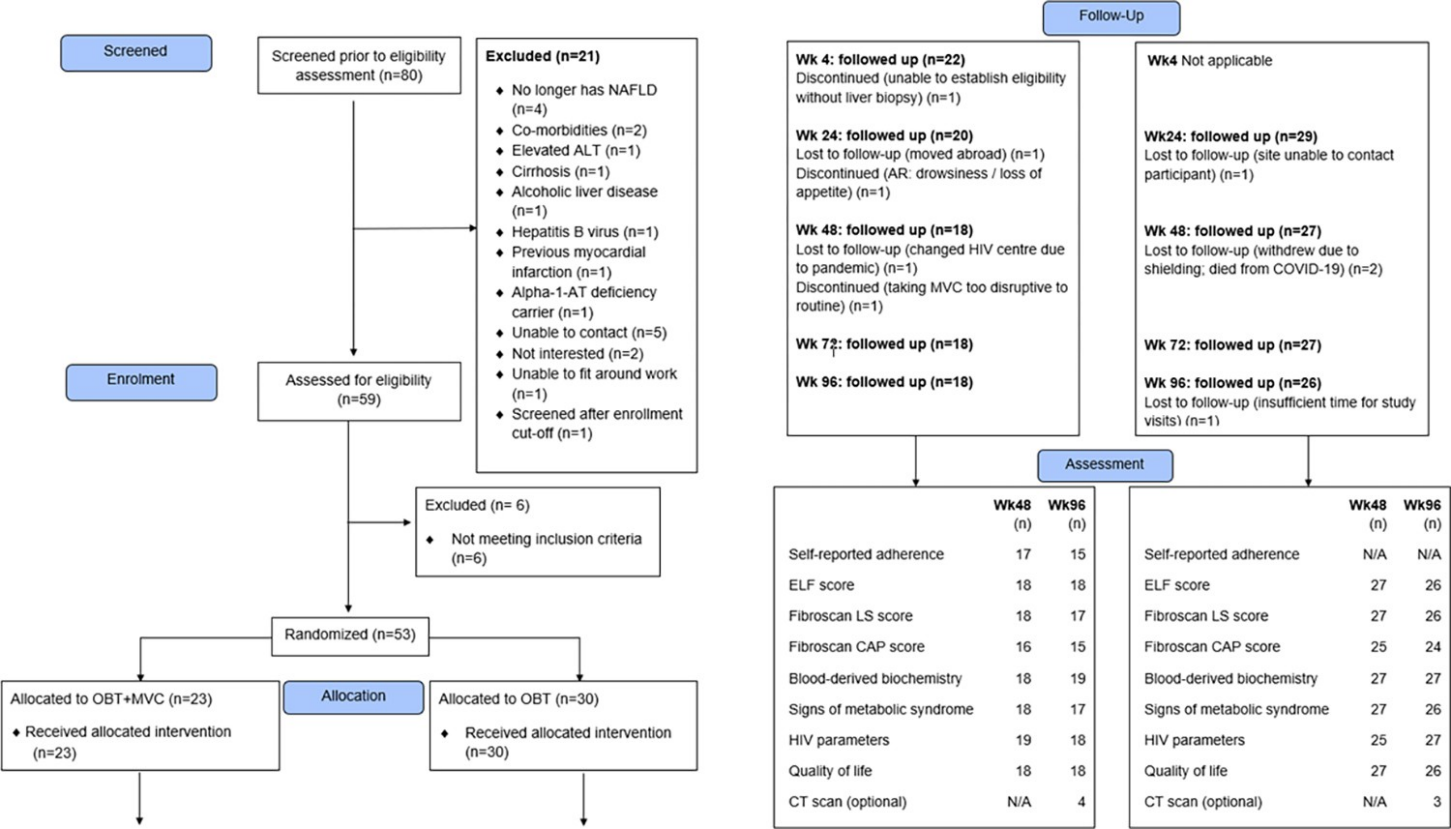
CONSORT flow diagram.

Eighty individuals were referred for screening, 21 (26%) and 6 (8%) of whom were excluded prior to and following attendance at the formal eligibility assessment, respectively. The remaining 53 (66%) individuals entered the randomized treatment portion of the trial and were included in analyses; 23 (43%) and 30 (57%) were allocated to MVC+OBT and OBT, respectively. The flowchart and reasons for participant exclusion are shown in Fig 1. Five participants in the MVC+OBT group (5/23, 22%) and four in the OBT group (4/30, 13%) discontinued the trial before completing the final visit.

### Baseline characteristics

Baseline demographic and clinical characteristics were broadly similar across groups (Table 1). Overall, 47 (89%) were male and 89% white; median (25^th^ centile - 75^th^ centile) age, BMI and waist circumference were 53 (IQR 46–59) years, 30 (IQR 26–35) kg/m^2^, and 106 cm (IQR 95–115) cm, respectively. Median ALT was 44 (IQR 31–69) IU/L, AST 32 (IQR 24–44) IU/L and GGT 42 (IQR 30–72) IU/L, HbA1c 38 (IQR 33–43) mmol/mol and fasting HDL: total cholesterol ratio 4.0 (IQR 3.5–4.8). Fifty-one percent fulfilled criteria for metabolic syndrome. The median baseline ELF, LSM and CAP scores were 9.1 (IQR 8.6–9.6), 6.2 (IQR 4.6–7.8) kPa and 325 (IQR 279–351) dB/m. Twenty-nine (55%) participants were receiving integrase strand transfer inhibitor (INSTI)-based cART and fifteen (28%) were receiving tenofovir alafenamide (TAF).

**Table 1:** Participant baseline characteristics.

	OBT+MVC			OBT			Total		
	N	Med/n	IQR/%	N	Med/n	IQR/%	N	Med/n	IQR/%
Age, years	23	51	38 to 59	30	55	49 to 61	53	53	46 to 59
Male	23	20	87%	30	27	90%	53	47	89%
White	23	19	83%	30	28	93%	53	47	89%
BMI, Kg/m^2^	23	28	26 to 32	30	31	26 to 35	53	30	26 to 35
Waist circumference, cm	23	102	95 to 113	30	108	96 to 116	53	106	95 to 115
Systolic blood pressure, mmHg	23	129	124 to 136	30	132	121 to 141	53	130	123 to 140
Antihypertensive therapy	23	4	17%	30	7	23%	53	11	21%
Lipid lowering therapy	23	9	39%	30	14	47%	53	23	43%
Glucose lowering therapy	23	2	9%	30	4	13%	53	6	11%
Duration HIV infection, years	23	16	12 to 23	29	14	9 to 22	52	15	9.5 to 23
CCR5 tropic*	23	12	80%	30	20	83%	53	32	82%
CD4 count, cells/mm^3^	23	702	546 to 1007	30	745	514 to 1055	53	702	545 to 1035
HbA1c, mmol/mol	23	38	32 to 42	30	39	35 to 45	53	38	33 to 43
ALT, U/L	23	45	31 to 62	29	44	29 to 69	52	44	30 to 69
AST, U/L	23	32	24 to 39	28	33	22 to 58	51	32	24 to 44
GGT, U/L	23	45	32 to 112	30	41	26 to 58	53	42	30 to 72
Fasting LDL cholesterol, mmol/L	21	2.8	2.3 to 3.3	28	2.8	1.8 to 3.1	49	2.8	2 to 3.2
Fasting HDL cholesterol, mmol/L	22	1.2	1 to 1.4	30	1.1	0.9 to 1.2	52	1.1	0.9 to 1.3
Fasting Triglycerides, mmol/L	22	1.7	1.2 to 3.0	30	1.8	1.3 to 2.3	52	1.7	1.3 to 2.5
Fasting HDL: cholesterol ratio	22	3.9	3.5 to 4.7	30	4.3	3.5 to 4.8	52	4.0	3.5 to 4.8
Metabolic syndrome	23	11	48%	30	16	53%	53	27	51%
Fibroscan median LS, kPa	22	6.4	4.9 to 8	29	5.7	4.6 to 7.7	51	6.2	4.6 to 7.8
Fibroscan CAP score, dB/m	21	337	281 to 349	27	320	277 to 352	53	325	279 to 351
ELF score	22	9.2	8.5 to 9.5	29	9.0	8.7 to 9.6	51	9.1	8.6 to 9.6
NNRTI-based cART	23	8	35%	30	8	27%	53	16	30%
INSTI-based cART	23	11	48%	30	18	60%	53	29	55%
PI-based cART	23	4	17%	30	4	13%	53	8	15%
TAF	23	5	22%	30	10	33%	53	15	28%

*Tropism testing from HIV proviral DNA sequencing was successful for 39/53 (74%)

cART combination antiretroviral therapy, CAP controlled attenuation parameter, INSTI integrase strand transfer inhibitor, LS liver stiffness, MVC maraviroc, NNRTI Non-nucleoside reverse transcriptase inhibitor, OBT optimised background therapy, PI protease inhibitor, TAF tenofovir alafenamide

### Completion of assessments

All mandatory 48 and 96 week clinical assessments were completed by 41 (77%) and 39 (74%) participants, respectively. At 48 / 96 weeks, CLDQ-NAFLD, SF36 and WPAI: SHIP questionnaires were completed by 45 (85%) / 44 (83%), 43 (81%) / 43 (81%) and 25 (47%) / 25 (47%) participants, respectively. The incompletely answered questions for WPAI: SHIP were related to work productivity. For optional CT scans, only seven (13%) participants, all in one site, undertook paired scans. Therefore, this analysis was excluded from the main results and is presented in Supplementary Table S2 (S1 File).

### Primary outcome measures–assessment of progression criteria against predefined targets

All individuals considered eligible after screening consented to participate, exceeding the target of 50%. The average recruitment rate was 2.9 per month over 18 months, exceeding the target of 2 per month. Participant retention was 45/53 (85%, 95% CI (72%, 93%)) and 44/53 (83%, 95%CI (70%, 92%)) by weeks 48 and 96, respectively, exceeding the target of 65%. At weeks 48 / 96, data completeness was 94% / 96% respectively, exceeding the target of 80%. There were no Serious Adverse Reactions, with 5/23 (22%, 95% CI (5%, 49%)) participants reporting AR by week 48 and no new AR by week 96 (Table 2). This did not meet the target of less than 10%. Self-reported adherence could not be measured as diary cards were not consistently completed or brought to appointments. Therefore, adherence was identified from counting of returned pills and was 92% (SD 7%), exceeding the target of 90%.

**Table 2:** Adverse Reactions (AR) to maraviroc.

Participant	Type of AR	Grade	No. days post BL	AR duration (days)	Caused by MVC?	Outcome	Discontinued MVC?
A	Generalised rash	Mild	3	5	Possibly	Resolved	No
	Vomiting	Mild	7	8	Possibly	Resolved	No
B	Dizziness	Mild	1	7	Definitely	Resolved	No
C	Worsening of restless legs	Moderate	32	557	Possibly	Resolved	Yes
D	Dizziness	Mild	2	21	Possibly	Resolved	No
E	Drowsiness	Moderate	28	182	Probably	Resolved	Yes
	Loss appetite	Moderate	28	182	Probably	Resolved	Yes

AR Adverse Reaction, BL baseline visit, MVC maraviroc

### Adverse events

Two SAEs were observed in the MVC+OBT group (community acquired pneumonia and gastro-oesophageal reflux disease with vomiting) and four in the OBT group (fatal COVID-19 pneumonia, urinary retention from benign prostatic hyperplasia, suicidal ideation, listeria meningitis). One SAE was observed in a participant prior to randomisation (spinal degenerative disease)

There were 71 AEs (3.1 per participant) and 62 AEs (2.1 per participant) in the MVC+OBT and OBT groups, respectively (Supplementary Table S3 in S1 File). Of five ARs, four were neurological: worsening of restless legs (n = 1), drowsiness with loss of appetite (n = 1) and dizziness (n = 2). The fifth individual experienced rash and vomiting possibly MVC-related. Two individuals with AR discontinued maraviroc, at 21 weeks and 81 weeks. All five ARs were of mild or moderate intensity and resolved. One additional individual discontinued maraviroc at week 14 due to disruption to their regimen from twice daily dosing.

### Secondary outcome measures

Table 3 shows the differences between treatment groups, comparing change from baseline to weeks 48 and 96 in clinical characteristics, including ALT, AST, lipids, HbA1c, CD4 count, ELF, LSM and CAP scores. The 95% CI were generally wide, indicating relatively imprecise estimation of the between-group difference in changes from baseline, due to the small sample size. In all cases, 95% CI included zero, consistent with there being no detectable differences between treatment groups. No cases of virological failure were observed. Blips, defined as a single VL 50–1000 c/ml followed by a VL<50c/ml, were seen in 5/23 (22%) and 3/30 (10%) individuals in the MVC+OBT versus OBT group respectively. Excluding blips identified at the baseline visit, the rate was 3/23 (13%) and 2/30 (7%), respectively.

**Table 3:** Difference in change from baseline in metabolic and liver parameters for the maraviroc + OBT versus OBT group with bootstrapped 95% confidence intervals.

	Week	Number of participants	Difference between MVC+OBT / OBT groups in change from baseline	95% LCL	95% UCL
BMI, kg/m^2^	48	45	0.1	-0.7	0.8
	96	45	0.1	-0.5	0.7
Waist circumference, cm	48	43	2.6	-4.0	9.2
	96	42	1.5	-6.4	9.3
CD4 cell count, cells/mm^3^	48	45	70	-39	179
	96	44	-47	-133	39.4
Fasting glucose, mmol/L	48	44	0.0	-0.8	0.7
	96	42	0.8	-0.5	2.2
HbA1c, mmol/mol	48	45	-2.3	-6.7	2.1
	96	45	2.5	-2.6	7.7
AST, U/L	48	43	-0.5	-6.9	5.9
	96	38	7.0	-2.8	16.8
ALT, U/L	48	44	-5.7	-21.1	9.7
	96	45	0.5	-19.8	20.7
Fasting triglycerides, mmol/L	48	44	0.7	0.0	1.4
	96	46	-0.2	-1.0	0.6
Fasting LDL, mmol/L	48	40	0.0	-0.4	0.4
	96	41	0.0	-0.4	0.5
Fasting HDL, mmol/L	48	44	0.0	-0.1	0.2
	96	46	0.1	-0.1	0.3
Fasting HDL: total chol. ratio	48	44	0.3	-0.6	1.1
	96	46	-0.3	-1.0	0.4
Fasting total chol., mmol/L	48	44	0.1	-0.4	0.6
	96	46	0.1	-0.4	0.6
Fibroscan median LS, kPa	48	43	-1.7	-3.7	0.4
	96	41	-0.6	-2.4	1.2
Fibroscan CAP score, dB/m	48	41	-15.1	-57.8	27.6
	96	39	-5.8	-40.0	28.5
ELF score	48	43	0.3	0.0	0.6
	96	42	0.1	-0.3	0.5

LCL, lower confidence limit; UCL upper confidence limit

Supplementary Table S4 in S1 File shows clinical characteristics comparing baseline, week 48 and 96 visits. Trends over time for most characteristics were similar comparing groups although two differences were observed. From baseline to week 96, decreases were observed in the median ALT (-8 IU/L) and LSM scores (-0.95kPa) for the MVC+OBT group versus increases in the OBT group (+4 IU/L and +0.65 kPa respectively). Consistent with this, median CAP score improvements over this period were greater in the MVC+OBT group (-59 dB/m versus -20 dB/m). However, 95% CI values for all characteristics were consistent with there being no change.

For QoL outcomes, comparing change from baseline through weeks 48 and 96 between treatment groups, 95% CI were wide indicating imprecise estimation of between-group differences. In all cases, 95% CI included zero, consistent with there being no detectable between-group differences (Table 4).

**Table 4:** Difference in change from baseline in quality of life outcomes for the maraviroc + OBT versus OBT group with bootstrapped 95% confidence intervals.

Outcome	Week	N	Difference between MVC+OBT / OBT groups in change from baseline	95% LCL	95% UCL
**CLDQ-NAFLD**	48	45	-0.3	-1.1	0.4
	96	44	-0.5	-1.3	0.2
**SF36**					
Physical function	48	45	-1.7	-11.4	8.1
	96	44	-1.0	-11.0	8.9
Role physical	48	44	1.4	-18.0	20.8
	96	44	13	-8.9	35.0
Role emotional	48	44	-0.6	-27.3	26.2
	96	44	-14.5	-36.0	6.9
Energy/fatigue	48	43	-0.2	-10.4	10.0
	96	43	-1.8	-11.9	8.3
Emotional wellbeing	48	43	6.0	-3.4	15.3
	96	43	2.6	-7.9	13.1
Social function	48	44	-4.1	-16.0	8.0
	96	44	-4.9	-19.2	9.4
Bodily pain	48	43	-1.3	-15.6	12.9
	96	43	-7.5	-19.7	4.7
General health	48	45	2.9	-7.1	12.9
	96	44	1.5	-7.2	10.3
**WPAI:SHP**					
% Work time missed	48	25	-10.5	-25.6	4.7
	96	25	-8.2	-22.8	6.3
% Impairment while working due to NAFLD	48	29	-2.6	-21.3	16.1
	96	30	-7.1	-21.0	6.7
% Overall work impairment due to NAFLD	48	25	-5.5	-27.6	16.7
	96	25	-13.7	-30.5	3.1
% Activity impairment due to NAFLD	48	43	1.3	-10.3	13.0
	96	43	0.1	-12.4	12.6

LCL, lower confidence interval, UCL upper confidence interval

CLDQ-NAFLD Chronic Liver Disease Questionnaire for NAFLD, SF36 36-item Short Form Survey, WPAI:SHP Work Productivity and Activity Impairment: Specific Health Problem

### Protocol deviations

There were 263 protocol deviations due to missing a study assessment (96, 37%); COVID-19 pandemic related factors resulting in assessments out of window (67, 25%); assessment out of window for other reasons (46, 17%); scheduling of the whole visit out of window (36, 14%); other reason (wrong stratification, incorrect randomisation number, missing data, IMP dispensing, incorrect eligibility assessment) (18, 7%).

## Discussion

These data provide preliminary evidence that add-on maraviroc as therapy for NAFLD without cirrhosis in PWH on effective cART may be safe, acceptable and feasible. We recruited ~90% of our target of 60 individuals, and, of those referred for possible participation, 66% were randomised, including all who met eligibility criteria at screen. Participant retention until week 96 was acceptable, achieving ~80%, despite the unprecedented circumstances of the SARS-CoV-2 pandemic. Adherence to maraviroc, taken twice daily by almost all participants, was high (>90%). The adverse reaction rate of 22% was numerically greater than the 10% pre-selected target; however, this figure fell within the 95% CI around 22% of 5%-49% indicating the uncertainty as to whether or not there was an increase in AR compared to the target. Taken in conjunction with findings that all adverse reactions resolved, including in the 9% who discontinued maraviroc, and that there were no serious adverse reactions, no clear evidence of a significant safety concern was identified It was notable that blip rates were numerically greater in the MVC+OBT versus OBT arms (22% versus 10%), but if baseline visit blips were excluded, these became 13% and 7% respectively. Although this remained higher in the MVC+OBT group, both rates are comparable to that seen in the clinic population for one of the participating sites with available published data (~10%) [39].

We observed differences in the changes of two participant characteristics during follow up between the two treatment groups. ALT and LSM scores improved in the MVC+OBT but worsened in the OBT group over 96 weeks. CAP scores declined in both groups but by a numerically greater level in the MVC+OBT group. These findings raise the possibility of an improvement in liver fat and fibrosis. However, the 95% CI for the differences contained zero so were consistent with there being no detectable differences, and the trial was not powered to evaluate for differences in these characteristics. A future RCT of add-on maraviroc with a larger sample size should assess for any differences in these outcomes.

There was a high level (>80%) of completion of questionnaires to assess QoL outcomes for CLDQ-NAFLD and SF36, but <50% completion for WPAI:SHIP. This was related to lack of responses to work productivity questions only, likely due to a combination of some participants being unemployed plus missing data. CLDQ-NAFLD and SF36 would therefore be more suitable for inclusion in a larger trial. Regarding QoL outcomes, 95% CI for differences between groups contained zero which was consistent with there being no detectable difference. Few individuals undertook optional CTs, either as this was not offered by sites or participant choice. This may be because CT is less acceptable than ultrasound for NAFLD follow up due to the increased radiation exposure and should not be included in a future RCT. The alternative imaging modality of Magnetic resonance imaging (MRI)–estimated proton density fat fraction (PDFF) is expensive and not routinely available.

Limited data on the potential efficacy of maraviroc as a treatment for NAFLD are available from previous studies. In a retrospective cohort in PWH, 74 individuals receiving maraviroc were compared to 312 who had never been exposed to maraviroc, matched for age, sex and CD4 count nadir. In the non-maraviroc group, a significant association was found between a marker of inflammation (hsCRP) and lipoprotein levels (LDL, TG, TC) at baseline and after 3 years. By contrast, in the maraviroc group, this relationship was observed at baseline but lost after 3 years. The incidence of non-AIDS-defining disease was lower in the maraviroc group but this was not statistically significant. The authors speculated that CCR5 inhibition may offer protection against a lipid-dependent inflammatory process [17].

In a phase III trial, PWH -NAFLD were randomised into four groups: OBT (n = 24), ± maraviroc (n = 23), metformin (n = 21) or maraviroc with metformin (n = 22). Hepatic fat scores were measured at baseline and week 48 using MRI-PDFF. Maraviroc ± metformin was found to be safe with acceptable tolerability but did not reduce liver fat scores compared to no adjunctive treatment, and LSM scores were not presented [40]. In a proof of concept, single arm, prospective study of OBT plus add-on maraviroc with HIV-NAFLD, liver biopsies were compared at baseline and week 48. No significant impact on liver histology was observed including an absence of change in CD4+, CD8+ or CD68+ immune cell infiltrates, NAFLD Activity Score, ballooning and steatosis or lipid metabolism, although only thirteen individuals were included [41].

We instituted a number of measures in response to the COVID-19 pandemic. First, time windows for visits were lengthened from ±1 to ±8 weeks to provide flexibility for staff redeployed to COVID-19 responsive work and for participants experiencing difficulty in accessing clinical sites. Second, virtual visits were permitted for follow up visits, excluding week 48 and 96. This was to provide focus on high completeness of the final dataset whilst enabling participants to avoid travel and clinical contact while self-isolating. Third, we regularly sought feedback from sites to identify problems early and work towards finding local solutions for keeping participants engaged, for example, mailing out IMP.

### Limitations

We used non-invasive assessment methods for hepatic steatosis and fibrosis, whilst the gold standard, liver biopsy, would have permitted more accurate classification of disease. However, recruiting a sufficient sample of PWH and mild NAFLD to a randomised study requiring consecutive liver biopsies presents feasibility challenges. We did not control for all ART classes, and emerging data have highlighted the possible association of INSTI and TAF with NAFLD progression, as well as a possible protective role for tenofovir disoproxil, although data are conflicting [42–44]. It would have been challenging to stratify for all ART agents possibly associated with risk of or protection from NAFLD in this small pilot study, including PIs, INSTI, TAF and TDF. Further, much of the emerging data highlighting an association between INSTI and/or TAF with NAFLD, and the negative association between NAFLD and TDF, became available after the last participant to this study had been recruited. A further limitation was that duration of HIV regimen was not controlled prior to participation. Additionally, 5/30 (17%) and 1/23 (4%) individuals in the OBT and MVC+OBT groups, respectively, switched one of more components of their backbone whilst on study; five were in-class switches and one an NNRTI to INSTI switch.Our cohort was ~90% white and male and therefore applicability to other groups, particularly women and black and other minority ethnic groups, is unclear.

### Considerations for an efficacy RCT of add-on MVC in HIV-NAFLD

Given conflicting data for associations between NAFLD development and different ART classes [42–44], eligibility criteria for a future RCT assessing efficacy of add-on MVC in HIV-NAFLD would need to carefully consider OBT regimen, for example, stratifying by PI, INSTI and TAF-containing therapy. Additional stratification may be required according to co-medications with proven or possible activity against NAFLD, such as tesamorelin, vitamin E or statins [9, 10, 45]. Given the uncertainty as to whether or not there was a greater AR rate compared to the pre-selected threshold, careful monitoring of ARs would also be needed. Although the blip rate whilst receiving MVC+OBT was comparable to that in the clinic population, the greater blip rate in the MVC+OBT versus OBT group highlights that close viral load monitoring may also be advised. Finally, given the need in this pilot for 7 sites to achieve 90% of the target, and considerations in a future trial around stratification by ARV backbone, to achieve a sample size of 103 participants per group in an efficacy RCT with 90% power, assuming a Cohen’s D of 0.5 (i.e. a medium effect size) and attrition rate of 17%, a multi-national trial would have greatest likelihood of recruiting to target.

## Conclusions

This study provides preliminary evidence that add-on maraviroc as therapy for HIV-NAFLD may be safe, feasible and acceptable. Although there were differences in the trend for ALT and LSM scores, with improvement in the MVC+OBT versus OBT group, the 95% CI contained zero, which was consistent with there being no detectable differences, and the study was not powered to evaluate changes in these characteristics. Overall, these data support a larger RCT assessing efficacy of add-on maraviroc on hepatic steatosis and fibrosis in PWH and NAFLD.

## Supporting information

S1 Checklist CONSORT 2010 checklist of information to include when reporting a pilot or feasibility trial. (DOC)

S1 File This file contains all supporting tables (S1-S4 Tables). (DOCX)

S2 File Trial protocol. (PDF)
