# Supplementary material for: Hepmarc: A 96 week randomised controlled feasibility trial of add-on maraviroc in people with HIV and non-alcoholic fatty liver disease
Source: PLoS One. 2023 Jul 14;18(7):e0288598. doi: 10.1371/journal.pone.0288598 (PMC10348519; doi:10.1371/journal.pone.0288598)
Supplement: S1 File — (DOCX) [file pone.0288598.s002.docx]

**Supplementary Table S1. Summary of trial procedures**

|  | **Screening visit**  **(-42d)** | **Baseline** | **Wk 4^1^**  **+/- 2d** | **Wk 24**  **+/- 7d** | **Wk 48**  **+/- 7d** | **Wk 72**  **+/- 7d** | **Wk 96**  **+/- 7d** | **Early Termination Visit** |
| --- | --- | --- | --- | --- | --- | --- | --- | --- |
| **Informed consent** | X |  |  |  |  |  |  |  |
| **Demographic data and medical history including full ART history and alcohol assessment** | x |  |  |  |  |  |  |  |
| **Randomisation** |  | x |  |  |  |  |  |  |
| **Vital signs** | x^2^ | X^2^ | X^2^ | X^2^ | X^2^ | x^2^ | x^2^ | x^2^ |
| **Physical examination including height, weight and waist circumference** | x^3^ | x^3,4^ |  | x^3,4^ | x^3,4^ | x^3,4^ | x^3,4^ | x^3,4^ |
| **ECG** | x |  |  |  |  |  |  |  |
| **Urine dip^5^ and pregnancy test (for WOCBP)** | x | x |  | x | x | x | X | X |
| **Concomitant medications** | x | x | X | X | X | x | x | x |
| **HIV associated conditions** | x | x |  | X | X | x | x | x |
| **Symptom & AE review** | X | X | X | X | X | x | x | x |
| **Diet and exercise history^6^** |  | x |  |  | x |  | x | x |
| **CLDQ:NAFLD, SF36, WPAI:SHP questionnaires** |  | X |  |  | X |  | X | X |
| **ELF Score** |  | X |  |  | X |  | x | x |
| **CD4/CD8 T cell count** | x |  |  |  | x |  | x | x |
| **HIV-1 RNA level** | x |  | x | x | x | x | x | x |
| **Proviral DNA Tropism^7^** |  | x |  |  |  |  |  |  |
| **Haematology^8^** | x | x | x | x | x | x | x | x |
| **Routine chemistry^9^** | X | x | x | x | x | x | x | x |
| **Fasting chemistry^10^** |  | X |  |  | X |  | X | X |
| **Additional chemistry^11^** | X |  |  |  | X |  | X | X |
| **HIV, HBV & HCV serology^12^** | x |  |  |  |  |  |  |  |
| **Full liver screen^13^** | x |  |  |  |  |  |  |  |
| **Ultrasound Liver^14^** | x |  |  |  |  |  |  |  |
| **Fibroscan^15^** | x |  |  |  | x |  | x | X |
| **CT liver : spleen attenuation ratio^16^** |  | X |  |  |  |  | X |  |
| **Drug dispensation^17^** |  | x | x | x | x | x |  |  |

1 Week 4 visit only for individuals receiving maraviroc. Bloods are unfasted.

2 HR, RR, Temp, BP, Lying and standing BP (postural BP at screening only and to be repeated if history indicates)

3 Height only at screening; weight and waist circumference at every visit except week 4

4 Symptom directed physical examination only

5 Point of care urine dip for haematuria, proteinuria, glycosuria, leucocytes and nitrites

6 Dietary history will be daily intake of olive oil, fruit, vegetables or salad, legumes, fish, wine, meat, white bread, rice and whole-grain bread)(22). Exercise history will be number of times per week exercise is undertaken, number of minutes of exercise per episode and type of exercise.

7 If no result within the preceding 24 weeks

8 Haemoglobin, white cell count and differential, eosinophils, platelets

9 Sodium, potassium, chloride, creatinine, urea, alanine aminotransferase (ALT), aspartate aminotransferase (AST), bilirubin, alkaline phosphatase (ALP), gamma glutamyltransferase (GGT), albumin, phosphate, CK, glucose (screening, weeks 4, 24 and 72); lipids (total cholesterol, HDL, LDL, triglycerides) (weeks 24 and 72 only)

10 Fasting glucose and fasting lipids (total cholesterol, HDL, LDL, triglycerides)

11 HbA1c

12 anti-HCV Ab, HCV RNA or HCV antigen, HBsAg; if no prior record of result: anti-HBcAb. HIV Ab/Ag only if no previous documented result

13 If no previous record of result: INR, ferritin, caeruloplasmin, copper, thyroid function, alpha-1 antitrypsin, anti-mitochondrial antibodies, anti-nuclear antibodies, anti-smooth muscle antibody, anti-liver/kidney/microsomal antibodies-1, coeliac serology

14 If no previous imaging (US, CT, MRI) result confirming fatty liver in the preceding 24 weeks

15 Includes both median stiffness and controlled attenuation parameter scores. To be performed within 7 days of the study visit.

16 Optional. To be performed within 7 days of the study visit. Preference is for the 7 days prior to baseline.

17 Only for individuals assigned to the maraviroc group

**Supplementary Table S2. CT liver : spleen attenuation results at baseline and 96 weeks**

|  | **No. of participants with CT liver : spleen attenuation score <1** | |
| --- | --- | --- |
|  | **Baseline** | **Week 96** |
| OBT | 3/5 (60%) | 3/3 (100%) |
| MVC+OBT | 2/6 (33%) | 0/4 |

MVC maraviroc, OBT optimised background therapy

**Supplementary Tables S3a (upper) and 2b (lower). All adverse events in the MVC+OBT and OBT groups, respectively.**

| **Adverse Event** | **Grade** | **Relatedness** | **Outcome** | **Treated** | **SAE** |
| --- | --- | --- | --- | --- | --- |
| Generalised rash | Mild | Possibly | Resolved | No | No |
| Vomit | Mild | Possibly | Resolved | No | No |
| Vomit | Mild | Possibly | Resolved | No | No |
| Urinary tract infection | Moderate | Not related | Resolved | Yes | No |
| Acute kidney injury | Moderate | Not related | Resolved | Yes | No |
| Community acquired pneumonia | Severe | Not related | Resolved | Yes | Yes |
| Impaired renal function (Post Acute Kidney Injury) | Moderate | Not related | Ongoing | No | No |
| Osteoporosis L2-4 spine | Moderate | Not related | Ongoing | Yes | No |
| Diarrhoea | Mild | Not related | Resolved | Yes | No |
| Osteoarthritis left knee | Moderate | Not related | Ongoing | Yes | No |
| Abdominal pain - unexplained | Mild | Not related | Resolved | Yes | No |
| Hearing loss right ear | Moderate | Not related | Ongoing | Yes | No |
| Lower respiratory tract infection | Mild | Not related | Resolved | Yes | No |
| Left shoulder pain | Mild | Not related | Ongoing | No | No |
| Headaches | Mild | Not related | Ongoing | Yes | No |
| Dizziness | Mild | Definite | Resolved | No | No |
| Diabetes mellitus Type 2 | Moderate | Not related | Ongoing | No | No |
| Haemorrhoids | Moderate | Not related | Ongoing | No | No |
| Constipation | Moderate | Not related | Resolved | Yes | No |
| Anaemia | Mild | Not related | Resolved | No | No |
| Rectal Lymphogranuloma Venereum | Mild | Not related | Resolved | Yes | No |
| Trauma right foot | Moderate | Not related | Resolved | Yes | No |
| Cold | Mild | Not related | Resolved | No | No |
| Diarrhoea + vomiting | Mild | Not related | Resolved | No | No |
| Throat Gonorrhoea | Mild | Not related | Resolved | Yes | No |
| Worsening of restless legs | Moderate | Possibly | Resolved | No | No |
| Shoulder pain | Moderate | Not related | Resolved | Yes | No |
| Perianal fissures/pile | Moderate | Not related | Ongoing | Yes | No |
| Tooth infection | Moderate | Not related | Resolved | Yes | No |
| Rash query cause on torso | Moderate | Not related | Ongoing | Yes | No |
| Right shoulder pain | Moderate | Not related | Resolved | Yes | No |
| Perineal abscess | Moderate | Not related | Resolved | Yes | No |
| Left knee meniscal injury | Moderate | Not related | Ongoing | Yes | No |
| Vitamin D deficiency | Moderate | Not related | Ongoing | Yes | No |
| Left sciatica | Moderate | Not related | Resolved | No | No |
| Menopause symptoms | Moderate | Not related | Ongoing | No | No |
| Poor diabetic control | Mild | Not related | Resolved | No | No |
| 'Cold' | Mild | Not related | Resolved | No | No |
| Tooth ache | Moderate | Not related | Ongoing | Yes | No |
| Worsening diabetic control | Moderate | Not related | Ongoing | No | No |
| Folliculitis | Mild | Unlikely | Resolved | No | No |
| Dizziness | Mild | Possibly | Resolved | No | No |
| Common Cold | Mild | Not related | Resolved | No | No |
| Peripheral neuropathy worsening in lower extremities | Moderate | Not related | Ongoing | No | No |
| Chronic Obstructive Pulmonary Disease | Moderate | Not related | Ongoing | Yes | No |
| Loss of consciousness | Moderate | Not related | Resolved | No | No |
| Panic Attack | Mild | Not related | Resolved | No | No |
| Common cold | Mild | Not related | Resolved | No | No |
| Bloating | Mild | Not related | Resolved | No | No |
| Sleeping difficulties | Mild | Not related | Ongoing | No | No |
| Acid reflux and vomiting | Severe | Not related | Resolved | Yes | Yes |
| Raised Blood Cholesterol Levels | Moderate | Not related | Ongoing | Yes | No |
| Raised Blood Triglyceride level | Severe | Not related | Ongoing | Yes | No |
| Drowsiness | Moderate | Probably | Resolved | No | No |
| Loss of appetite | Moderate | Probably | Resolved | No | No |
| Anaemia | Mild | Not related | Ongoing | No | No |
| Raised HbA1C | Mild | Not related | Ongoing | No | No |
| Raised Fasting Glucose | Mild | Not related | Ongoing | No | No |
| Elevated HIV viral Load | Mild | Not related | Resolved | No | No |
| Exacerbation of osteoarthritis | Mild | Not related | Resolved | Yes | No |
| Left submandibular lymphadenopathy | Mild | Not related | Unknown | No | No |
| Covid 19 | Mild | Not related | Resolved | Yes | No |
| Raised Lipids | Mild | Not related | Ongoing | No | No |
| Cold symptoms | Mild | Not related | Resolved | Yes | No |
| Low mood | Moderate | Unlikely | Ongoing | No | No |
| Anxiety | Moderate | Unlikely | Unknown | Yes | No |
| Episode of Psychosis | Moderate | Not related | Resolved | Yes | No |
| Genital warts | Mild | Not related | Resolved | Yes | No |
| Flu like symptoms | Mild | Not related | Resolved | No | No |
| Elevated HbA1c | Mild | Not related | Ongoing | No | No |
| Elevated GGT | Mild | Not related | Ongoing | No | No |

| **Adverse Event** | **Grade** | **Relatedness** | **Outcome** | **Treated** | **SAE** |
| --- | --- | --- | --- | --- | --- |
| Penile sore | Mild | Not related | Resolved | Yes | No |
| Raised ALT | Severe | Not related | Resolved | No | No |
| Impaired glucose tolerance | Moderate | Not related | Ongoing | No | No |
| Diverticular disease | Moderate | Not related | Ongoing | No | No |
| Type 2 diabetes | Moderate | Not related | Ongoing | No | No |
| Hyperlipidaemia | Moderate | Not related | Ongoing | Yes | No |
| Probable Covid-19 | Moderate | Not related | Resolved | Yes | No |
| Sciatica | Moderate | Not related | Ongoing | Yes | No |
| Ankle swelling | Mild | Not related | Resolved | No | No |
| Poorly controlled diabetes | Moderate | Not related | Ongoing | No | No |
| Looser bowel motions | Moderate | Not related | Resolved | No | No |
| Fatigue | Moderate | Not related | Resolved | No | No |
| Chest infection | Moderate | Not related | Resolved | Yes | No |
| Query contact dermatitis | Moderate | Not related | Ongoing | Yes | No |
| Aubergine allergy | Moderate | Not related | Resolved | No | No |
| Right wrist pain | Moderate | Not related | Resolved | Yes | No |
| Left shoulder musculoskeletal pain | Moderate | Not related | Ongoing | Yes | No |
| Coryzal illness | Mild | Not related | Ongoing | Yes | No |
| Excision of right epididymal lesion | Moderate | Not related | Resolved | Yes | No |
| Worsening of asthma | Moderate | Not related | Resolved | Yes | No |
| Gastric reflux | Moderate | Not related | Resolved | Yes | No |
| Worsening of asthma | Moderate | Not related | Ongoing | Yes | No |
| Gastric Reflux | Moderate | Not related | Ongoing | Yes | No |
| Depression | Moderate | Not related | Ongoing | No | No |
| Chronic Obstructive Pulmonary Disorder | Moderate | Not related | Ongoing | No | No |
| Bronchitis | Moderate | Not related | Resolved | Yes | No |
| Dizziness | Moderate | Not related | Resolved | No | No |
| Insomnia | Moderate | Not related | Resolved | No | No |
| Common cold | Mild | Not related | Resolved | Yes | No |
| Post Vaccine syndrome | Moderate | Not related | Resolved | No | No |
| Common Cold | Mild | Not related | Resolved | Yes | No |
| Excessive sweating | Mild | Not related | Ongoing | No | No |
| Back Pain | Moderate | Not related | Ongoing | No | No |
| Rectal bleeding/Likely haemorrhoids | Mild | Not related | Ongoing | Yes | No |
| Hospital admission for retention of urine | Severe | Not related | Resolved | Yes | Yes |
| Listeria Meningitis | Severe | Not related | Resolved | Yes | Yes |
| Raised Creatine Kinase | Mild | Unlikely | Resolved | No | No |
| Worsening Liver Function Tests | Moderate | Unlikely | Ongoing | No | No |
| Suicidal ideation | Moderate | Not related | Resolved | Yes | Yes |
| Elevated HbA1c | Mild | Not related | Ongoing | No | No |
| Rectal polyp | Mild | Not related | Ongoing | No | No |
| fungal rash | Mild | Not related | Resolved | Yes | No |
| Raised HbA1C | Mild | Not related | Ongoing | No | No |
| Covid 19 | Mild | Not related | Resolved | No | No |
| Trigger finger | Mild | Not related | Unknown | Yes | No |
| Possible Bechet's disease | Mild | Not related | Ongoing | Yes | No |
| Anxiety | Mild | Not related | Ongoing | No | No |
| Cold | Mild | Not related | Resolved | Yes | No |
| Raised cholesterol | Mild | Not related | Ongoing | No | No |
| Neurosyphilis | Mild | Not related | Resolved | Yes | No |
| Microscopic haematuria on urine dip | Mild | Not related | Ongoing | No | No |
| Elevated Triglycerides | Mild | Not related | Ongoing | No | No |
| Community Acquired Pneumonia - Covid 19 | Severe | Not related | Unknown | Yes | Yes |
| Shingles eye socket | Mild | Not related | Resolved | Yes | No |
| Lichen sclerosis | Mild | Not related | Ongoing | Yes | No |
| Covid 19 | Moderate | Not related | Resolved | No | No |
| HIV viral Load detectable | Mild | Not related | Resolved | No | No |
| Chest pain radiating down left arm | Mild | Not related | Ongoing | Yes | No |
| Bell's Palsy | Mild | Not related | Ongoing | Yes | No |
| Cough/Chest Infection | Moderate | Not related | Resolved | Yes | No |
| Fever and Flu like symptoms | Moderate | Not related | Resolved | No | No |
| Chest Infection | Moderate | Not related | Resolved | Yes | No |

**Supplementary Table S4. Clinical characteristics at baseline, week 48 and week 96.**

|  |  | MVC + OBT (n=23) | | | | OBT only (n=30) | | | | Total (n=53) | | | |
| --- | --- | --- | --- | --- | --- | --- | --- | --- | --- | --- | --- | --- | --- |
|  | Week | n | median | 25th c. | 75th c. | n | median | 25th c. | 75th c. | n | median | 25th c. | 75th c. |
|  | BL | 23 | 702 | 546 | 1007 | 30 | 745 | 514 | 1055 | 53 | 702 | 545 | 1007 |
| CD4 count, cells/mm3 | wk 48 | 18 | 743 | 563 | 1035 | 27 | 758 | 535 | 1001 | 45 | 755 | 561 | 1001 |
|  | wk 96 | 19 | 651 | 454 | 852 | 25 | 743 | 570 | 935 | 44 | 734 | 529 | 904 |
|  | BL | 23 | 28 | 26 | 32 | 30 | 31 | 26 | 35 | 53 | 30 | 26 | 35 |
| BMI, Kg/m2 | wk 48 | 18 | 27 | 25 | 30 | 27 | 29 | 26 | 35 | 45 | 28 | 26 | 33 |
|  | wk 96 | 18 | 27 | 25 | 31 | 27 | 30 | 26 | 36 | 45 | 28 | 26 | 34 |
|  | BL | 23 | 102 | 95 | 115 | 30 | 108 | 96 | 116 | 53 | 106 | 95 | 115 |
| Waist circumference, cm | wk 48 | 17 | 100 | 96 | 112 | 26 | 100 | 94 | 116 | 43 | 100 | 96 | 113 |
|  | wk 96 | 17 | 102 | 99 | 115 | 25 | 105 | 99 | 116 | 42 | 104 | 99 | 116 |
|  | BL | 23 | 5.0 | 4.6 | 6.5 | 29 | 5.2 | 4.8 | 6.3 | 52 | 5.2 | 4.6 | 6.4 |
| Fasting glucose, mmol/L | wk 48 | 18 | 5.3 | 5.0 | 5.6 | 26 | 5.3 | 4.7 | 6.4 | 44 | 5.3 | 4.9 | 5.9 |
|  | wk 96 | 18 | 5.5 | 4.6 | 6.6 | 24 | 5.5 | 4.8 | 7.4 | 42 | 5.5 | 4.7 | 6.7 |
|  | BL | 23 | 38 | 32 | 42 | 30 | 39 | 35 | 45 | 53 | 38 | 33 | 43 |
| HbA1c, mmol/mol | wk 48 | 18 | 38 | 31 | 42 | 27 | 40 | 35 | 46 | 45 | 39 | 34 | 46 |
|  | wk 96 | 18 | 39 | 34 | 46 | 27 | 39 | 35 | 52 | 45 | 39 | 35 | 49 |
|  | BL | 23 | 45 | 31 | 62 | 29 | 44 | 29 | 69 | 52 | 44 | 30 | 69 |
| ALT, IU/L | wk 48 | 18 | 40 | 27 | 59 | 27 | 38 | 33 | 75 | 45 | 38 | 30 | 69 |
|  | wk 96 | 19 | 37 | 22 | 67 | 27 | 48 | 32 | 77 | 46 | 47 | 30 | 67 |
|  | BL | 23 | 32 | 24 | 39 | 28 | 33 | 22 | 45 | 51 | 32 | 24 | 44 |
| AST, IU/L | wk 48 | 18 | 33 | 22 | 40 | 27 | 30 | 23 | 46 | 45 | 30 | 23 | 41 |
|  | wk 96 | 16 | 32 | 28 | 44 | 24 | 30 | 22 | 45 | 40 | 31 | 25 | 44 |
|  | BL | 22 | 1.7 | 1.2 | 3.0 | 30 | 1.8 | 1.3 | 2.3 | 52 | 1.7 | 1.3 | 2.5 |
| Fasting TG, mmol/L | wk 48 | 18 | 1.9 | 1.4 | 2.4 | 26 | 1.4 | 1.1 | 2.4 | 44 | 1.8 | 1.3 | 2.4 |
|  | wk 96 | 19 | 1.7 | 1.4 | 2.1 | 27 | 1.8 | 1.2 | 2.8 | 46 | 1.8 | 1.3 | 2.3 |
|  | BL | 21 | 2.8 | 2.3 | 3.3 | 28 | 2.8 | 1.8 | 3.1 | 49 | 2.8 | 2.0 | 3.2 |
| Fasting LDL, mmol/L | wk 48 | 17 | 2.3 | 2.0 | 3.1 | 25 | 2.3 | 2.0 | 3.0 | 42 | 2.3 | 2.0 | 3.0 |
|  | wk 96 | 18 | 2.5 | 2.1 | 3.3 | 26 | 2.5 | 1.9 | 2.7 | 44 | 2.5 | 1.9 | 2.9 |
|  | BL | 22 | 1.2 | 1.0 | 1.4 | 30 | 1.1 | 0.9 | 1.2 | 52 | 1.1 | 0.9 | 1.3 |
| Fasting HDL, mmol/L | wk 48 | 18 | 1.1 | 0.9 | 1.6 | 26 | 1.0 | 0.9 | 1.2 | 44 | 1.0 | 0.9 | 1.3 |
|  | wk 96 | 19 | 1.1 | 0.9 | 1.4 | 27 | 1.0 | 0.8 | 1.1 | 46 | 1.0 | 0.9 | 1.3 |
|  | BL | 22 | 5.0 | 3.9 | 5.5 | 30 | 4.4 | 3.9 | 5.0 | 52 | 4.5 | 3.9 | 5.2 |
| Fasting TC, mmol/L | wk 48 | 18 | 4.5 | 3.9 | 5.4 | 26 | 4.0 | 3.6 | 4.6 | 44 | 4.3 | 3.7 | 5.1 |
|  | wk 96 | 19 | 4.5 | 3.9 | 5.9 | 27 | 4.3 | 3.6 | 4.9 | 46 | 4.3 | 3.7 | 5.2 |
|  | BL | 22 | 3.9 | 3.5 | 4.7 | 30 | 4.3 | 3.5 | 4.8 | 52 | 4.0 | 3.5 | 4.8 |
| Fasting HDL:cholesterol ratio | wk 48 | 18 | 3.7 | 3.1 | 4.5 | 26 | 3.8 | 3.3 | 4.7 | 44 | 3.8 | 3.2 | 4.7 |
|  | wk 96 | 19 | 4.0 | 3.0 | 4.9 | 27 | 4.4 | 3.5 | 5 | 46 | 4.2 | 3.4 | 4.9 |
|  | BL | 22 | 9.2 | 8.5 | 9.5 | 29 | 9.0 | 8.7 | 9.6 | 51 | 9.1 | 8.6 | 9.6 |
| ELF | wk 48 | 18 | 9.4 | 8.8 | 9.7 | 27 | 8.7 | 8.6 | 9.3 | 45 | 8.9 | 8.6 | 9.6 |
|  | wk 96 | 18 | 9.1 | 8.8 | 9.5 | 26 | 9.2 | 8.4 | 9.7 | 44 | 9.1 | 8.7 | 9.6 |
|  | BL | 22 | 6.4 | 4.9 | 8.0 | 29 | 5.7 | 4.6 | 7.7 | 51 | 6.2 | 4.6 | 7.8 |
| Fibroscan median LS, kPa | wk 48 | 18 | 5.6 | 3.9 | 6.4 | 27 | 5.6 | 4.4 | 9 | 45 | 5.6 | 4.3 | 6.9 |
|  | wk 96 | 17 | 5.4 | 4.0 | 6.7 | 26 | 6.4 | 4.4 | 6.9 | 43 | 5.8 | 4.4 | 6.9 |
|  | BL | 21 | 337 | 281 | 349 | 27 | 320 | 277 | 352 | 48 | 325 | 279 | 351 |
| Fibroscan CAP score, dB/m | wk 48 | 16 | 282 | 248 | 320 | 25 | 311 | 266 | 351 | 41 | 295 | 258 | 347 |
|  | wk 96 | 15 | 278 | 230 | 323 | 24 | 300 | 248 | 348 | 39 | 282 | 248 | 336 |
