# Supplementary material for: Hepmarc: A 96 week randomised controlled feasibility trial of add-on maraviroc in people with HIV and non-alcoholic fatty liver disease
Source: PLoS One. 2023 Jul 14;18(7):e0288598. doi: 10.1371/journal.pone.0288598 (PMC10348519; doi:10.1371/journal.pone.0288598)
Supplement: S2 File — (PDF) [file pone.0288598.s003.pdf]

A phase IV, open-label, pilot study investigating non-invasive markers of hepatic fibrosis in people living with HIV-1 and non-alcoholic fatty liver disease randomised to receiving optimised background therapy (OBT) plus maraviroc or OBT

HEPMARC Study

This protocol has regard for the HRA guidance and order of content

FULL/LONG TITLE OF THE TRIAL

A phase IV, open-label, pilot study, investigating non-invasive markers of hepatic fibrosis in people living with HIV-1 and non-alcoholic fatty liver disease randomised to receiving optimised background therapy (OBT) plus maraviroc or OBT

SHORT STUDY TITLE / ACRONYM

HEPMARC Study

PROTOCOL VERSION NUMBER AND DATE

5.0 13th September 2019

RESEARCH REFERENCE NUMBERS

**IRAS Number:**	228763
**SPONSORS Number:**	228763
**FUNDERS Number:**	Viiv Project Number 207967

SIGNATURE PAGE

The undersigned confirm that the following protocol has been agreed and accepted and that the Chief Investigator agrees to conduct the trial in compliance with the approved protocol and will adhere to the principles outlined in the Medicines for Human Use (Clinical Trials) Regulations 2004 (SI 2004/1031), amended regulations (SI 2006/1928) and any subsequent amendments of the clinical trial regulations, GCP guidelines, the Sponsor's SOPs, and other regulatory requirements as amended.

I agree to ensure that the confidential information contained in this document will not be used for any other purpose other than the evaluation or conduct of the clinical investigation without the prior written consent of the Sponsor.

I also confirm that I will make the findings of the study publicly available through publication or other dissemination tools without any unnecessary delay and that an honest accurate and transparent account of the study will be given; and that any discrepancies from the study as planned in this protocol will be explained.

For and on behalf of the Study Sponsor:

Signature: Date:
..../....../......

Name (please print):

....

Position:
....

Chief Investigator:

Signature: Date:
..../....../......

Name: (please print):

....

Statistician:

Signature:
....

Name: (please print):

....

Position:
....

PROTOCOL ACCEPTANCE FORM

HEPMARC

A phase IV, open-label, pilot study, investigating non-invasive markers of hepatic fibrosis in people living with HIV-1 and non-alcoholic fatty liver disease randomised to receiving optimised background therapy (OBT) plus maraviroc or OBT

Chief Investigator:	Dr Daniel Bradshaw
Sponsor:	Brighton & Sussex University Hospitals NHS Trust,<br>Royal Sussex County Hospital,<br>Eastern Road,<br>Brighton BN2 5BE, United Kingdom

I agree to conduct the study in accordance with the current protocol, Version 5.0

____

Principal Investigator's Name (print)

______________________________ ______________

Principal Investigator's Signature Date

LIST of CONTENTS

GENERAL INFORMATION	Page No.
TITLE PAGE	1
RESEARCH REFERENCE NUMBERS	2
SIGNATURE PAGE	3
PROTOCOL ACCEPTANCE FORM	4
LIST of CONTENTS	5
KEY TRIAL CONTACTS	6
TRIAL SUMMARY	7
FUNDING	9
ROLES & RESPONSIBILITIES OF TRIAL MANAGEMENT COMMITTEES, GROUPS AND INDIVIDUALS	10
LIST OF ABBREVIATIONS	11
TRIAL FLOW CHART	13
**SECTION**	
1. BACKGROUND	17
2. RATIONALE	18
3. OBJECTIVES AND OUTCOME MEASURES/ENDPOINTS	19
4. TRIAL DESIGN	21
5. STUDY SETTING	22
6. ELIGIBILITY CRITERIA	22
7. TRIAL PROCEDURES	24
8. TRIAL MEDICATION	32
9. PHARMACOVIGILANCE	34
10. STATISTICS AND DATA ANALYSIS	43
11. DATA HANDLING	48
12. MONITORING, AUDIT & INSPECTION	49
13. ETHICAL AND TRIAL ADMINISTRATION	49
14. DISSEMINATION POLICY	53
15. REFERENCES	53
16. APPENDICES	56

KEY TRIAL CONTACTS

Chief Investigator	Dr Daniel Bradshaw<br>daniel.bradshaw2@nhs.net<br>0208 327 6237
Trial Co-ordinator	
Sponsor	Scott Harfield scott.harfield@nhs.net<br>Tel: 01273 696955 ext 7497<br>Head of Research and Development, Brighton and Sussex University Hospitals NHS Trust
Funder(s)	Viiv Healthcare (a subsidiary of GSK Pharmaceuticals)
Clinical Trials Unit	Brighton and Sussex Clinical Trials Unit (CTU)<br>bsctu@bsms.ac.uk
Key Protocol Contributors	Dr Daniel Bradshaw daniel.bradshaw2@nhs.net<br>Dr Yvonne Gilleece y.gilleece@nhs.net<br>Professor Mark Nelson mark.nelson@imperial.ac.uk<br>Nicky Perry n.perry@bsms.ac.uk<br>Professor Sumita Verma s.verma@bsms.ac.uk<br>Dr Stephen Bremner s.bremner@bsms.ac.uk<br>Dr Iga Abramowicz i.abramowicz@bsms.ac.uk
Statistician	Dr Stephen Bremner s.bremner@bsms.ac.uk
Trials pharmacist	Tenesa Sargent<br>tenesa.sargent@nhs.net<br>Tel: 01273 664 437
Committees	Trial Management Group<br>Data Safety and Monitoring Board<br>Trial Steering Committee

TRIAL SUMMARY

Trial Title	**A phase IV, open-label pilot study investigating non-invasive markers of hepatic fibrosis in people living with HIV-1 and non-alcoholic fatty liver disease randomised to receiving OBT plus maraviroc or OBT**	
Internal ref. no. (or short title)	HEPMARC Study	
Clinical Phase	Phase IV	
Trial Design	Open-label, randomised controlled trial; pilot study	
Trial Participants	People living with well-controlled HIV-1 and non-alcoholic fatty liver disease	
Planned Sample Size	Total number n=60	
Treatment duration	96 weeks	
Follow up duration	96 weeks	
Planned Trial Period	March 2018 –October 2021 (LPLV)	
	Objectives	Outcome Measures
Primary	Assessment of the feasibility and acceptability of the addition of maraviroc to OBT for the treatment of NAFLD in people living with HIV	Proportion of eligible individuals approached who are successfully recruited<br>Monthly participant recruitment rate<br>Participant retention in the study at 48 and 96 weeks in the maraviroc and non-maraviroc assigned groups<br>Proportion of participants for whom there is missing data at 48 and 96 weeks in the maraviroc and non-maraviroc assigned groups<br>Proportion of participants reporting adverse events at 48 and 96 weeks in the maraviroc and non-maraviroc assigned groups<br>Level of reported adherence to the study drug at 48 and 96

		weeks in those assigned to the maraviroc group
Secondary	Assessment of the effect of maraviroc on non-invasive serum markers of hepatic fibrosis in people living with HIV and NAFLD	Change in the mean ELF score at 48 and 96 weeks in individuals randomised to receiving MVC plus OBT versus OBT alone
	Assessment of the effect of maraviroc on non-invasive markers of hepatic fibrosis in people living with HIV and NAFLD as assessed by transient elastography	Change in the mean Fibroscan score at 48 and 96 weeks in individuals randomised to receiving MVC plus OBT versus OBT alone<br>Change in mean Fibroscan CAP score at 48 and 96 weeks in individuals randomised to receiving MVC plus OBT versus OBT alone
	Assessment of the effect of maraviroc on non-invasive markers of hepatic fibrosis in people living with HIV and NAFLD as assessed by imaging	Change in % with CT liver: spleen attenuation ratio <1.0 at 96 weeks in individuals randomised to receiving MVC plus OBT versus OBT alone
	Assessment of the effect of maraviroc on blood-derived biochemistry in people living with HIV and NAFLD	Change in the mean for fasting HDL:cholesterol ratio, LDL, HDL, triglyceride, and glucose plus HbA1c and ALT at 48 and 96 weeks in individuals randomised to receiving MVC plus OBT versus OBT alone
	Assessment of the effect of maraviroc on clinical signs of the metabolic syndrome in people living with HIV and NAFLD	Change in the mean BMI, weight and waist circumference at 48 and 96 weeks in individuals randomised to receiving MVC plus OBT versus OBT alone

	Assessment of the effect of maraviroc on HIV parameters in people living with NAFLD<br><br>Assessment of quality of life measures in people living with HIV and NAFLD	Change in the mean CD4 count and in the % with an undetectable HIV VL at 48 and 96 weeks in individuals randomised to receiving MVC plus OBT versus OBT alone<br><br>Responses at 48 and 96 weeks in individuals randomised to receiving MVC plus OBT versus OBT alone on the CLDQ:NAFLD, SF-36 and WPAI-SHP questionnaires
Investigational Medicinal Product(s)	Maraviroc	
Formulation, Dose, Route of Administration	Film-coated tablets; oral; dose dependent on interactions with co-prescribed medications (according to SmPC recommendations)	

FUNDING AND SUPPORT IN KIND

FUNDER(S)	FINANCIAL AND NON FINANCIALSUPPORT GIVEN
Viiv Healthcare	**£214,550.77**

ROLES AND RESPONSIBILITIES OF TRIAL MANAGEMENT COMMITTEES / GROUPS & INDIVIDUALS

Trial Management Group (TMG)

This will consist of the chief investigator, operational manager, trial manager, data manager, senior statistician, statistician and research nurses.

The TMG will be responsible for the trial set-up, the day-to-day running of the trial and the release of any trial results or publications according to the BSCTU SOPs.

Data Safety and Monitoring Board (DSMB)

This will consist of the following individuals who are independent from the study team: a consultant in HIV, a consultant hepatologist, and a statistician. The composition of the DSMB will be defined in the DSMB charter.

The DSMB will leverage their scientific expertise and objectively advise the TSC on data interpretation and appropriate modifications to the study if appropriate. The Board's terms of reference, roles and responsibilities will be defined in a charter in accordance with the relevant BSCTU SOP.

Trial Steering Committee (TSC)

This will consist of the chief investigator, co-investigators, operational manager, trial manager, data manager, senior statistician, a consultant in HIV, a consultant hepatologist and a patient representative.

The TSC will have oversight of the trial conduct. The Committee's terms of reference, roles and responsibilities will be defined in a charter in accordance with the relevant BSCTU SOP.

Protocol contributors

All members of the Trial Management Group contributed to writing the protocol with support from the Jaffa Panel, the patient and public involvement (PPI) lay research panel for BSUH.

The final decision regarding the design, conduct, data analysis, manuscript writing and dissemination of results will rest with the sponsor.

KEY WORDS: Maraviroc; HIV-1; non-alcoholic fatty liver disease (NAFLD); non-invasive markers of hepatic fibrosis; Enhanced Liver Fibrosis (ELF) score; Fibroscan

LIST OF ABBREVIATIONS

Define all unusual or ‘technical’ terms related to the trial. Add or delete as appropriate to your trial. Maintain alphabetical order for ease of reference.

AE	Adverse Event
AR	Adverse Reaction
BSUH	Brighton and Sussex University Hospital NHS Trust
BSCTU	Brighton and Sussex Clinical Trials Unit
CA	Competent Authority
CI	Chief Investigator
CRO	Contract Research Organisation
CTA	Clinical Trial Authorisation
CTIMP	Clinical Trial of Investigational Medicinal Product
DDI	Drug drug interaction
DILI	Drug induced liver injury
DSMB	Data Safety and Monitoring Board
DSUR	Development Safety Update Report
EC	European Commission
eCRF	Electronic Case Report Form
EMEA	European Medicines Agency
EU	European Union
EUCTD	European Clinical Trials Directive
EudraCT	European Clinical Trials Database
EudraVIGILANCE	European database for Pharmacovigilance
GCP	Good Clinical Practice
GMP	Good Manufacturing Practice
IB	Investigator Brochure
ICF	Informed Consent Form
ICH	International Conference on Harmonisation of technical requirements for registration of pharmaceuticals for human use.
IMP	Investigational Medicinal Product
IMPD	Investigational Medicinal Product Dossier
ISF	Investigator Site File
ISRCTN	International Standard Randomised Controlled Trials Number

LU	Lawson Unit (HIV department of Brighton and Sussex University Hospitals NHS Trust)
MA	Marketing Authorisation
MHRA	Medicines and Healthcare products Regulatory Agency
MS	Member State
MVC	Maraviroc
NAFLD	Non Alcoholic Fatty Liver Disease
NHS R&D	National Health Service Research & Development
NIMP	Non-Investigational Medicinal Product
OBT	Optimised Background Therapy
PI	Principal Investigator
PIC	Participant Identification Centre
PIS	Participant Information Sheet
QA	Quality Assurance
QC	Quality Control
QP	Qualified Person
RCT	Randomised Controlled Trial
REC	Research Ethics Committee
SAE	Serious Adverse Event
SAR	Serious Adverse Reaction
SDV	Source Data Verification
SOP	Standard Operating Procedure
SmPC	Summary of Product Characteristics
SSI	Site Specific Information
SUSAR	Suspected Unexpected Serious Adverse Reaction
TMG	Trial Management Group
TMF	Trial Master File
TSC	Trial Steering Committee
WOCBP	Women of Child Bearing Potential

TRIAL FLOW CHART

Table 1. Summary of trial procedures

	Screening visit (-42d)	Baseline	Wk 4[1] +/- 2d	Wk 24 +/- 7d	Wk 48 +/- 7d	Wk 72 +/- 7d	Wk 96 +/- 7d	Early Termination Visit
**Informed consent**	X							
**Demographic data and medical history including full ART history and alcohol assessment**	X							
**Randomisation**		X						
**Vital signs**	X[2]	X[2]	X[2]	X[2]	X[2]	X[2]	X[2]	X[2]
**Physical examination including height, weight and waist circumference**	X[3]	X[3,4]		X[3,4]	X[3,4]	X[3,4]	X[3,4]	X[3,4]
**ECG**	X							
**Urine dip[5] and pregnancy test (for WOCBP)**	X	X		X	X	X	X	X
**Concomitant medications**	X	X	X	X	X	X	X	X

**HIV associated conditions**	X	X		X	X	X	X	X
**Symptom & AE review**	X	X	X	X	X	X	X	X
**Diet and exercise history**		x			x		X	X
**CLDQ:NAFLD, SF36, WPAI:SHP questionnaires**		X			X		X	X
**ELF Score**		X			X		X	X
**CD4/CD8 T cell count**	X				X		X	X
**HIV-1 RNA level**	X		X	X	X	X	X	X
**Proviral DNA Geno2Pheno (tropism) Test[6]**		X						
**Haematology[7]**	X	X	X	X	X	X	X	X
**Routine chemistry[8]**	X	X	X	X	X	X	X	X
**Fasting chemistry[9]**		X			X		X	X
**Additional chemistry[10]**	X				X		X	X

**HIV, HBV & HCV serology**[11]	X							
**Full liver screen**[12]	X							
**Ultrasound Liver**[13]	X							
**Fibroscan**[14]	X				X		X	X
**CT liver:spleen attenuation ratio**[15]		X					X	
**Drug dispensation**[16]		X	X	X	X	X		

1 Wk 4 visit only for individuals receiving maraviroc. Bloods are unfasted.

2 HR, RR, Temp, BP, Lying and standing BP (postural BP at screening only and to be repeated if history indicates)

3 Height only at screening; weight and waist circumference at every visit except week 4

4 Symptom directed physical examination only

5 Point of care urine dip for haematuria, proteinuria, glycosuria, leucocytes and nitrites

6 If no result within the preceding 24 weeks

7 Haemoglobin, white cell count and differential, eosinophils, platelets

8 Sodium, potassium, chloride, creatinine, urea, alanine aminotransferase (ALT), aspartate aminotransferase (AST), bilirubin, alkaline phosphatase (ALP), gamma glutamyltransferase (GGT), albumin, phosphate, CK, glucose (screening, weeks 4, 24 and 72); lipids (total cholesterol, HDL, LDL, triglycerides) (weeks 24 and 72 only)

9 Fasting glucose and fasting lipids (total cholesterol, HDL, LDL, triglycerides)

10 HbA1c

11 anti-HCV Ab, HCV RNA or HCV antigen, HBsAg; if no prior record of result: anti-HBcAb. HIV Ab/Ag only if no previous documented result

12 If no previous record of result: INR, ferritin, caeruloplasmin, copper, thyroid function, alpha-1 antitrypsin, anti-mitochondrial antibodies, anti-nuclear antibodies, anti-smooth muscle antibody, anti-liver/kidney/microsomal antibodies-1, coeliac serology

13 If no previous imaging (US, CT, MRI) or liver biopsy result confirming fatty liver in the preceding 24 weeks. This may be performed at any point between the screening and baseline visits.

14 Includes both median stiffness and controlled attenuation parameter scores. This may be performed at any point between the screening and baseline visits. For the week 48 and 96 week visits, to be performed within 7 days of the study visit.

15 Optional. To be performed within 7 days of the study visit. Preference is for the 7 days prior to baseline.

16 Only for individuals assigned to the maraviroc group

STUDY PROTOCOL

A phase IV, open-label, pilot study, investigating non-invasive markers of hepatic fibrosis in people living with HIV-1 and non-alcoholic fatty liver disease randomised to receiving optimised background therapy (OBT) plus maraviroc or OBT

1 BACKGROUND

Liver disease represents an important cause of morbidity and mortality in HIV-infected cohorts in industrialised countries. In particular, data from several cohorts has identified a high prevalence of non-alcoholic fatty liver disease (NAFLD) of between 30-50% [1, 2]. Risk factors for NAFLD are widely recognised and largely associated with lifestyle factors include high BMI, type II diabetes mellitus, dyslipidaemia, smoking and high alcohol consumption as well as HIV-1 infection [3]. As NAFLD is an important risk factor for hepatic fibrosis, including cirrhosis, it represents an increasingly important comorbidity.

Several approaches are recommended for the treatment of NAFLD. These include addressing underlying risk factors including weight loss through dietary modification and increased exercise, reduction in alcohol consumption, smoking cessation and glycaemic control where applicable [4].

Pharmacological interventions include vitamin E and pioglitazone [4]. However, a relative lack of efficacy of currently available interventions has led to increasing interest in novel therapeutic approaches.

It is widely known that the chemokine CCL5/RANTES, the ligand for CCR5, plays an important role in the progression of hepatic inflammation and fibrosis. CCR5 mediates intrahepatic immune cell interactions that promote activation and migration of Kupffer cells and hepatic stellate cells which in turn promote inflammation and hepatic fibrosis [5, 6]. It could be postulated, therefore, that antagonism of this pathway would reduce hepatic pathology [6-8]

Maraviroc (MVC) is a drug licensed for the treatment of HIV-1 infection in both treatment-naïve and –experienced individuals, where the infecting strain is R5 tropic [9, 10]. It inhibits the binding of HIV-1 gp120 to the CCR5 co-receptor and therefore prevents the virus from entering its target cell. The property of MVC targeting the CCR5 receptor and thereby antagonising CCL5-CCR5 mediated interactions has led to interest in its potential anti-inflammatory benefits additional to its anti-HIV activity, including in the liver and brain [11-13]. Furthermore, there are over 7 years of data relating to the safety of MVC in humans and therefore MVC is considered to be well tolerated. In particular, it has also been demonstrated to be well tolerated in a cohort of patients with chronic liver disease caused by HIV/HCV or HIV/HBV coinfection [14].

In vitro, maraviroc reduces the release of pro-inflammatory cytokines implicated in fibrosis from immortalised human hepatic stellate cells, with reduced levels of extracellular matrix proteins (ECM) including type 1 collagen, alpha-SMA, TGF-1b, MMP-2 and MMP-9 [15]. In the mouse model, maraviroc has also been demonstrated to reduce the rate of hepatic fibrosis. Thirty-two male C57BL/6 mice were

randomised to receiving a normal or high fat diet (HFD), with or without MVC. Comparing, HFD versus HFD/MVC mice, the latter group exhibited a lower degree of hepatic steatosis at 16 weeks as well as lower hepatic CCL5/RANTES expression [16].

Furthermore, in HIV/HCV-coinfected individuals receiving MVC-containing ART, MVC reduced hepatic fibrosis progression. In this retrospective analysis of individuals followed up for a median of 18 months who were not on anti-HCV therapy, hepatic fibrosis was assessed through the AST:platelet ratio index (APRI). For 38 individuals receiving MVC over 2 years, 59% (n=27) had unchanged fibrosis stage and 29% (n=8) showed regression whilst 11% (n=3) progressed [12]. Early data from transient elastography studies also demonstrated a benefit of MVC on liver stiffness in HIV/HCV-coinfected individuals receiving MVC-based cART[17]

Finally, a novel antiretroviral, cenicriviroc (CVC), an antagonist of both CCR2 and CCR5, has been recently evaluated in ART-naïve individuals. Individuals (n=143) with CCR5-tropic HIV-1 were randomised to receive CVC 100mg, CVC 200mg or efavirenz (EFV) in combination with emtricitabine/tenofovir. Liver disease was assessed through the Enhanced Liver Fibrosis (ELF) biomarker index (calculated from serum levels of hyaluronic acid (HA), propeptide of type III procollagen (PIIINP) and tissue inhibitor of metalloproteinase-1 (TIMP-1)) at baseline and 48 weeks. At the CVC 200mg dose, a significant decrease in the ELF score was noted, a finding not seen for individuals treated with CVC 100mg or EFV[18]. A study investigating the potential benefit of CVC on histological changes in liver activity score and hepatic fibrosis in HIV-uninfected patients with biopsy-proven NASH is currently recruiting [19].

Following these observations, it is plausible that the CCR5-receptor antagonist MVC may have anti-fibrotic effects in vivo in HIV-monoinfected individuals with NAFLD. However, no study has yet investigated this.

2 RATIONALE

There is a high prevalence of NAFLD (30-40%) amongst HIV-infected patients, with at least 10-20% of these individuals likely to progress to steatohepatitis (NASH), a risk factor for hepatic fibrosis and cirrhosis [20]. There are currently few treatments for this condition, although recent data on cenicriviroc, an R2 and R5 antagonist, suggest it may have a beneficial impact in HIV-infected subjects [18] with further data on CVC awaited from a NASH study in HIV-uninfected subjects (Phase 2b) [19] . This raises the question as to whether or not the R5-antagonist MVC will demonstrate similar efficacy in NAFLD.

2.1 Assessment and management of risk

Maraviroc is licensed for the treatment of R5 tropic HIV-1 infection and is well known to be a safe and effective antiretroviral agent. Individuals who are intolerant or allergic to maraviroc, or in whom there is a potential risk of severe illness, such as those with previously diagnosed severe cardiovascular disease, will be excluded from the study.

The dose used will be that recommended in the SPC following published data in randomised clinical trials, and which is dependent on predicted interactions with the existing antiretroviral regimen or with other concomitantly prescribed agents.

This trial is categorised as:

- **Type B** Somewhat higher than the risk of standard medical care. The trial involves an authorised IMP being used for a new indication.

3 OBJECTIVES AND OUTCOME MEASURES/ENDPOINTS

3.1 Primary objectives

The primary objective will be to assess the feasibility and acceptability of the addition of maraviroc to OBT, versus maintenance of OBT alone, in the treatment of NALFD in people living with HIV. This will be assessed with respect to the following:

a) Acceptability of recruitment into the study to eligible individuals
b) Monthly participant recruitment rate
c) Participant retention rate
d) Completeness of the data set
e) Proportion of individuals reporting adverse events
f) Self-reported adherence to the study drug

The specific time points with respect to the above are provided in the ‘primary measures’ section below. The finding of confirmed feasibility and acceptability of the treatment approach of addition of maraviroc to effective cART in this population will support the establishment of larger, double-blind, placebo-controlled RCT to assess the efficacy of this intervention definitively.

3.2 Secondary objectives

a) Assessment of the effect of addition of maraviroc to OBT, versus continuing OBT alone, on non-invasive serum markers of hepatic fibrosis by 48 and 96 weeks in people living with HIV and NAFLD

b) Assessment of the effect of addition of maraviroc to OBT, versus continuing OBT alone, on non-invasive markers of hepatic fibrosis in people living with HIV and NAFLD by 48 and 96 weeks as measured by transient elastography

c) Assessment of the effect of addition of maraviroc to OBT, versus continuing OBT alone, on non-invasive markers of hepatic fibrosis in people living with HIV and NAFLD by 48 and 96 weeks as measured by computerised tomography [21].

d) Assessment of the effect of addition of maraviroc to OBT, versus continuing OBT alone, on clinical signs of the metabolic syndrome in people living with HIV and NAFLD by 48 and 96 weeks.

e) Assessment of the effect of addition of maraviroc to OBT, versus continuing OBT alone on blood-derived biochemistry in people living with HIV and NAFLD by 48 and 96 weeks.

f) Assessment of the effect of addition of maraviroc to OBT, versus continuing OBT alone on HIV parameters in people living with HIV and NAFLD by 48 and 96 weeks.

g) Assessment of the effect of addition of maraviroc to OBT, versus continuing OBT alone on quality of life in people living with HIV and NAFLD by 48 and 96 weeks.

3.3 Outcome measures/endpoints

Primary endpoint:

The primary objective will be assessed with respect to the following measures:

a) Proportion of eligible individuals approached who are successfully recruited
b) Monthly participant recruitment rate
c) Participant retention in the study at 48 and 96 weeks in the maraviroc and non-maraviroc assigned groups
d) Proportion of participants for whom there is missing data at 48 and 96 weeks in the maraviroc and non-maraviroc assigned groups
e) Proportion of participants reporting adverse events at 48 and 96 weeks in the maraviroc and non-maraviroc assigned groups
f) Level of self-reported adherence to the study drug at 48 and 96 weeks in those allocated to the maraviroc group

Secondary endpoints:

a) Mean change in the ELF score by 48 and 96 weeks comparing the maraviroc and non-maraviroc groups.

b) Mean change in Fibroscan stiffness by 48 and 96 weeks comparing the maraviroc and non-maraviroc groups

c) Mean change in the Fibroscan Controlled Attenuation Parameter (CAP) score by 48 and 96 weeks comparing the maraviroc and non-maraviroc groups

d) Change in the % with a CT liver:spleen attenuation ratio <1.0 by 96 weeks comparing the maraviroc and non-maraviroc groups

e) Mean change in blood-derived biochemistry by 48 and 96 weeks comparing the maraviroc and non-maraviroc groups: fasting HDL:chol ratio, LDL, HDL, TG, glucose, plus Hb1AC and ALT

f) Mean change in clinical signs of the metabolic syndrome comparing the maraviroc and non-maraviroc groups by 48 and 96 weeks: BMI, waist circumference and weight

g) Mean change in HIV parameters comparing the maraviroc and non-maraviroc groups: CD4 cell count and % with undetectable HIV VL.

h) Differences in the quality of life of participants in the maraviroc and non-maraviroc groups by 48 and 96 weeks as assessed by responses to the chronic liver disease questionnaire for NAFLD (CLDQ:NAFLD), and the SF-36 and WPAI:SHP questionnaires.

4 TRIAL DESIGN

This is a Phase IV, open-label, randomised, dual arm pilot study to assess the feasibility and potential efficacy of addition of maraviroc to OBT versus remaining on OBT alone in HIV-infected individuals with non-alcoholic fatty liver disease.

Randomisation will be stratified according to:

(1) current exposure or past history of ≥ 6 months exposure to protease inhibitor (PI)-containing antiretroviral therapy versus no current exposure and < 6 months past exposure to PI- containing therapy and

(2) BMI ≥ 25 versus < 25 and

(3) current exposure to a lipid-lowering agent*

(4) diabetes mellitus status (DM 1 or 2 versus no DM)

*HMG CoA reductase inhibitors eg statins

Cholesterol absorption inhibitor eg ezetimibe

Bile acid binding drugs eg cholestyramine

Fibrates

Omega 3 fatty acids

Stratification will be undertaken to balance the treatment groups on important prognostic factors (ie to prevent confounding) given that high BMI [2] and concurrent administration of PIs [1] may be associated with faster fibrosis progression in some studies. Statin use has conversely been associated with relative protection from hepatic fibrosis progression in some NAFLD studies [22]. Diabetes mellitus has been associated with faster hepatic fibrosis progression in several studies [2].

5 STUDY SETTING

The setting will be UK HIV centres. Potentially eligible patients will be identified from the HIV department by their clinicians from review of medical notes and imaging reports.

6 ELIGIBILITY CRITERIA

6.1 Inclusion criteria

(1) Aged 18 years and older

(2) HIV-1 infected with durably suppressed (≥ 6 months) HIV VL (<50 copies/ml)

NB. One HIV VL blip (50-200 copies/ml) is allowed in the 6 months prior to screen.

(3) Has evidence of NAFLD on hepatic imaging (USS, CT or MRI) or liver biopsy either at screen or in the 6 months prior to screen

(4) Provides written, informed consent to participate

(5) Is willing to comply with the protocol requirements

(6) If female and of child bearing potential, is using effective birth control methods (as agreed by the investigator) and willing to continue practicing these birth control measures during the trial and for at least 30 days after the end of the trial.

Note: Women who are postmenopausal for least 2 years, women with a total hysterectomy, and women who have a tubal ligation are considered of non-childbearing potential

(7) **If male, and sexually-active with female partners of child bearing potential, is using effective barrier contraception,** and willing to continue using this during the trial and for at least 30 days after the end of the trial

6.2 Exclusion criteria

(1) Severe cardiovascular disease including known angina or history of myocardial infarction

(2) History of postural hypotension, defined as a reduction in the systolic blood pressure of ≥ 20mmHg after standing for at least one minute

(3) Individuals previously exposed to MVC

(4) HIV viral load detectable (≥50 copies/ml). One blip (VL 50-200 copies/ml) within 6 months prior to screen is allowed.

(5) Current HCV or HBV (HBcAb-positive, HBsAg-negative is permitted; anti-HCV Ab positive with HCV RNA or HCV antigen negative for ≥ 6 months following treatment or spontaneous clearance is permitted)

(6) Other chronic liver disease including but not exclusively: cirrhosis, alcohol-related liver disease, autoimmune hepatitis, primary biliary cirrhosis, primary sclerosing cholangitis, haemochromatosis, Wilson's disease, alpha-1 antitrypsin deficiency, non-cirrhotic portal hypertension, drug-induced as deemed by a hepatologist
Note: alcohol-related liver disease includes liver disease in the presence of excess alcohol intake as defined according to EASL guidelines 2016 (ie >20g/day or >17 units/week for women and >30g/day or >26 units/week for men).

(7) ALT or AST > 5x the ULN (where ULN is defined as 41 IU/L)

(8) Severe renal insufficiency (creatinine clearance < 30 mL/min)

(9) HIV-2 infection

(10) Known allergy or intolerance to MVC or its constituents including hypersensitivity to peanuts or soya

(11) If female, pregnancy or breastfeeding

(12) Individuals currently taking medications or herbal agents that are contraindicated with MVC including St John's Wort.

7 TRIAL PROCEDURES

The schedule of assessments is summarised in Table 1

7.1 Recruitment

7.1.1 Patient identification

Clinical staff in the HIV department will identify participants by (i) review of a database of patients in the HIV department with known fatty liver disease, (ii) pre- identification of patients due to attend a routine pre-arranged follow up in the HIV department and (iii) review of medical notes during routine clinical follow up.. Potentially eligible participants will have had evidence of NAFLD on hepatic imaging. Clinical staff identifying patients will be members of the direct care team or by research nurses or doctors working within the same HIV multidisciplinary team. A medically qualified doctor on the study delegation log will confirm eligibility.

Anonymised information on participants who are not randomised / registered for CONSORT reporting will include:

- age,
- gender,
- ethnicity
- the reason not eligible for trial participation, or if they are eligible but declined

7.1.2 Screening Visit

Potentially eligible participants will be invited to attend for an appointment, having been provided with a participant information sheet. Adequate time (at least 24 hours) will be allowed for questions and to consider the study before agreeing to participate. The investigator or designee will provide adequate explanation of the aims, methods, objectives and potential hazards of the study. It will also be explained to the individual that they are free to refuse or withdraw from the study for any reason without detriment to their future care or treatment. Written, informed consent will then be taken by the investigator or co-investigator and this is mandatory prior to any study procedures

. The following evaluations must be performed within 42 days prior to randomisation:

- assessment of subject eligibility according to the inclusion and exclusion criteria
- demographics

- medical history including HIV-associated conditions, nadir CD4 count, alcohol assessment, recreational drug history and history of liver disease including result of any previous liver biopsy
- full antiretroviral history
- review of non-antiretroviral medication (any medication taken within the last 28 days)
- history of allergies, both drug and non-drug
- physical examination including height, weight and waist circumference
- vital signs (pulse, respiratory rate, lying and standing blood pressure, temperature)
- urine: macroanalysis and, for women of child bearing potential, urine pregnancy test
- laboratory confirmation of HIV-1 infection (only if documentation of such confirmation does not already exist)
- hepatitis B surface antigen and hepatitis C antibody and either HCV RNA or HCV antigen
- laboratory evaluations: full blood count, urea, electrolytes, glucose, liver function, creatinine kinase (CK), phosphate, HbA1c
- full liver screen if none previously recorded (ferritin, caeruloplasmin, copper, anti-SMA, AMA, ANA, anti-LKMA, coeliac serology, alpha-1 antitrypsin, clotting, thyroid function)
- CD4/CD8 counts and %
- HIV-1 RNA by licensed assay
- ECG
- USS liver (if no hepatic imaging or liver biopsy result is available within the preceding six months)
- Fibroscan

7.2 Consent

The Principal Investigator (PI) will retain overall responsibility for the informed consent of participants at site and will ensure that any person delegated responsibility to participate in the informed consent process is duly authorised, trained and competent to participate according to the ethically approved protocol, principles of Good Clinical Practice (GCP) and Declaration of Helsinki.

Informed consent will be obtained prior to the participant undergoing procedures that are specifically for the purposes of the trial and are outside standard routine care. The participant will remain free to withdraw at any time from the trial without giving reasons and without prejudicing his/her further treatment. Where a participant is required to re-consent or new information is required to be provided to a participant it will be the responsibility of the PI to ensure this is done in a timely manner.

The PI takes responsibility for ensuring that all vulnerable subjects are protected and participate voluntarily in an environment free from coercion or undue influence.

Consent will be received by the PI or co-investigator and will be obtained during the Screening Visit. Adequate explanation of the aims, methods, objectives and potential hazards of the study will be

provided to potential participants. A participant information sheet will be provided and adequate time allowed for questions and to consider the study, as well as to consider options for treatment outside the study, before agreeing to participate. Each participant will sign a consent form, a copy will put in the medical notes, one copy filed in the study centre file and the participant will be provided with a copy. If they decline to take a copy this will be documented in their medical notes.

7.3 The randomisation scheme

After written informed consent has been received and eligibility established, subjects will be randomised 1:1 into the maraviroc and non-maraviroc groups (Groups A and B respectively) at the baseline visit (day 1). In addition, there will be stratification according to (1) current exposure or past history of ≥ 6 months exposure to protease inhibitor (PI)-containing antiretroviral therapy versus no current exposure and less than 6 months past exposure to PI-containing therapy and (2) BMI ≥ 25 versus < 25 and (3) current exposure to a lipid-lowering agent and (4) being diabetic type 1 or 2 vs non-diabetic.

The rationale for this stratification is to attempt to reduce the potential for imbalance in factors considered a priori to be prognostic of ELF. Elevated BMI may be associated with faster progression of hepatic fibrosis in NAFLD patients. In addition, exposure to PI-containing therapy has in some studies been associated with accelerated fibrosis progression [1]. In other studies, lipid-lower therapies may reduce fibrosis progression [22]. Finally, diabetes mellitus has been identified as one of the strongest predictors of fibrosis progression in NAFLD patients [2].

The web-based Sealed Envelope[TM] system will be used to allocate individuals randomly to the maraviroc or non-maraviroc groups. The statistician will provide the randomisation list. The HEPMARC Randomisation Guide should be followed by the study team.

7.3.1 Method of implementing the allocation sequence

Sealed Envelope[TM] web based randomisation system will be used Investigators randomise patients by completing an on-screen form with patient details, stratification factors, inclusion and exclusion criteria. Investigators are immediately shown the treatment allocation. Trial managers have real-time access to recruitment statistics and are notified by email of every new randomisation. The randomisation application conforms to the requirements of FDA 21 CFR part 11, Electronic Records; Electronic Signatures and ICH GCP.

No-one can delete records from the randomisation database, so that all randomisations have to be accounted for. Audit log files detailing all activity on the randomisation system are available to the trial manager.

7.4 Blinding

Blinding will not be used. The rationale for this is:

(1) This is a pilot study. However, if results do indicate a possible benefit of maraviroc in NAFLD, they may be used to inform the design of a larger, blinded placebo-controlled randomised controlled trial.

(2) It is not considered likely that individuals in the maraviroc versus non-maraviroc group will differentially change their behaviour with respect to risk of fibrosis progression (eg dietary modifications, increase in exercise, reduction in alcohol consumption or other factor) and therefore the use of placebo is not considered essential for this pilotstudy.

7.5 Baseline data

The following baseline visit data will be collected:

- Targeted physical examination including BMI, weight, waist circumference
- Concomitant medicines check
- Blood draw for FBC, U+Es, LFTs, phosphate, CK,
- Blood draw for ELF score
- Blood draw for HIV-1 CCR5 coreceptor tropism (if no result in the previous 24 weeks)
- Urinary pregnancy test (WOCBP only) and dipstick
- eCRF completion including data transfer
- CLDQ:NAFLD, SF36, WPAI:SHP questionnaires
- Dietary history (daily intake of olive oil, fruit, vegetables or salad, legumes, fish, wine, meat, white bread, rice and whole-grain bread)[23] and exercise history (**number of times per week exercise is undertaken, number of minutes of exercise per episode and type of exercise)**
- Fasted glucose
- Fasted lipids (total cholesterol, LDL, HDL, triglycerides
- CT liver : spleen attenuation ratio *(optional)* following the protocol for an unenhanced CT abdomen. This may take place within 7 days of the baseline visit, with preference for the 7 days prior.

At baseline, all individuals will also be offered appropriate standard of care interventions regarding their NAFLD diagnosis in line with current NICE guidelines [4]. This will consist of advice regarding dietary modification including offer of referral to dietician; advice regarding exercise recommendations; advice regarding alcohol consumption with respect to national recommended limits and offer of referral to alcohol services where indicated. In addition, referral to the HIV/hepatology joint clinic or other specialist service will be recommended where appropriate.

7.6 Trial assessments

Please see Table 1 for details of the timeline for trial visits and which procedures and assessments will be performed at each visit.

The following are recorded at the baseline visit and then every 24 weeks until the final visit at 96 weeks (i.e. day 1, week 24, week 48, week 72 and week 96):

- history and if necessary symptom-directed examination
- adherence to maraviroc as assessed by (i) self reported adherence through completion of a diary card and (ii) pill counting by pharmacy/research team member.
- weight and BMI
- waist circumference
- liver function, renal function, phosphate, CK, glucose (fasted at baseline, weeks 48 and 96), and lipid profile (fasted at baseline, weeks 48 and 96)(blood draw)
- FBC (blood draw)
- HIV viral load (blood draw)
- Urine dipstick macroanalysis and, for WOCBP, pregnancy testing

The following are recorded at the baseline visit and then every 48 weeks until the final visit at 96 weeks (i.e. day 1, week 48 and week 96):

- CD4/CD8 cell count and % (blood draw)
- HbA1c (blood draw)
- ELF score (blood draw)
- CLDQ:NAFLD questionnaire
- SF-36 questionnaire
- WPAI-SHP questionnaire
- Dietary and exercise history

The following is recorded at screen, week 48 and week 96:

- Fibroscan. For screening, the Fibroscan may be performed up to 42 days prior to baseline. For the week 48 and 96 visits this may be performed within 7 days of the study visit.

The following **optional investigation** will be performed at the baseline visit and at the 96 week visit:

- CT liver : spleen attenuation ratio. This may be performed within 7 days of the study visit with preference for the 7 days prior to baseline.

An additional week 4 safety visit will be required for those individuals in the maraviroc group, with the following procedures being performed:

- assessment of any drug toxicity through targeted history and, if required, examination
- liver function, renal function, CK, phosphate, glucose, full blood count (blood draw)
- HIV viral load (blood draw)

The following will be performed at screening only (if no hepatic imaging or liver biopsy result is available in the preceding 24 weeks):

- USS liver

The following will be performed at baseline only (if not available in the preceding 24 weeks):

- CCR5 tropism (blood draw)

Early termination visit

Those individuals who withdraw from the study early will be requested to attend an early termination visit, during which the following assessments will be performed:

- history and examination
- adherence to maraviroc as assessed by (i) self reported adherence and (ii) pill counting by pharmacy/research team member.
- weight and BMI
- waist circumference
- liver function, renal function, CK, phosphate, fasting glucose, HbA1c and fasting lipid profile (blood draw)
- FBC (blood draw)
- HIV viral load (blood draw)
- ELF score (blood draw)
- Urine dipstick for macroanalysis and, for WOCBP, pregnancy testing
- CLDQ:NAFLD questionnaire
- SF-36 questionnaire

- WPAI-SHP questionnaire
- Dietary and exercise history
- Fibroscan

7.7 Withdrawal criteria

A subject is free to withdraw from the study at any time. In addition, the Investigator may decide, for reasons of medical prudence, to withdraw a subject. If a subject discontinues study medication dosing, every attempt should be made to keep the subject in the study and continue to perform the required study-related procedures and follow-up procedures. If this is not possible or acceptable to the subject or Investigator, the subject may be withdrawn from the study.

Study medication may also be discontinued in the following instances:

1. If the subject withdraws his/her consent.

2. If the investigator considers in the interest of the subject (i.e. intercurrent illness, unacceptable toxicity) that it is best for them to withdraw their consent.

3. The subject fails to comply with the protocol requirements or fails to cooperate with the investigator.

4. Pregnancy during the course of the study.

The date and reasons for the withdrawal will be clearly stated on the subject's eCRF and source document. Every attempt should be made to arrange follow up visits for subjects who are withdrawn from the trial (including where individuals fall pregnant).

Subjects withdrawing from the trial may be replaced if considered necessary by the Chief Investigator.

Toxicity Management

In the event of toxicity or intolerance to MVC in this study, subjects should be managed as in standard clinical practice. This may involve discontinuing MVC in some cases.

7.8 Storage and analysis of samples

Blood samples will be collected from patients at each study visit.

Routine biochemistry, haematology and virology testing will be performed through routine laboratory procedures. Samples will be delivered immediately within the internal courier system from the clinic room to the laboratory.

The following blood volumes will be required:

5mL gold top (SST) for LFTs, U+Es, lipids, phosphate, CK, and thyroid function

4mL EDTA for HbA1c

4mL EDTA for full blood count

5mL gold top (SST) for ferritin, copper, caeruloplasmin, alpha-1 antitrypsin, coeliac serology, and liver autoantibodies

2mL grey top (oxalate/fluoride) for glucose

2mL blue top (trisodium citrate) for INR

4mL EDTA for CD4/CD8 cell counts

5mL gold top (SST) for anti-HIV antibody/antigen, anti-HCV antibody, HCV antigen and HBsAg

8mL EDTA for HCV RNA

8mL EDTA for HIV RNA

8mL EDTA for HIV tropism

5mL gold top (SST) for ELF score (assays for TIMP-1, PIIINP, HA) *[research bloods]*

For the ELF score, samples will be frozen at -80C and stored at site, as described in the Laboratory Manual, and at the end of the study, batched and couriered on dry ice to the Centre for Hepatology, University College London, and assayed for the three components noted above.

Blood for the tropism assay will be couriered from the pathology service to the relevant laboratory according to routine procedures.

Total maximum volume of blood for each visit is as below:

Screen 47mL, baseline 24mL, week 4 19mL, week 24 19mL, week 48 32mL, week 72 19mL, week 96 32mL, ETV 28mL

Total maximum volume of blood in the study will be: 192mL

No blood samples will be stored after they have been tested for the designated test, except for the viral serology and viral PCR blood samples; surplus samples from these specimens are, as a routine, stored on site for 2 years as part of clinical care for the purpose of retrospective viral testing, should this be required for the patient's subsequent care (eg HIV resistance testing).

Blood tubes for ELF score will be provided by the sponsor.

Samples will be appropriately labelled in accordance with the trial procedures to comply with the 1998 Data Protection Act. Biological samples collected from participants as part of this trial will be transported, stored, accessed and processed in accordance with national legislation relating to the use and storage of human tissue for research purposes and such activities shall at least meet the requirements as set out in the 2004 Human Tissue Act.

Refer to the study Laboratory Manual for further site specific instructions.

For the creatinine clearance value calculations: https://www.mdcalc.com/creatinine-clearance-cockcroft-gault-equation web based calculator should be used and the obtained value should be verified according to the patient's BMI.

7.9 End of trial

The end of the trial will be the 96 week visit of the last participant.

8 TRIAL MEDICATION

8.1 Name and description of investigational medicinal product(s)

Maraviroc (Celsentri)

This is a licensed drug indicated with other antiretroviral medications for treatment-experienced adults infected with only CCR5-tropic HIV-1. It is supplied as a film-coated tablet. The recommended dose is 150mg, 300mg or 600mg twice daily depending on interactions with co-administered antiretroviral therapy and other medicinal products. For individuals with a creatinine clearance of between 30 mL/min and 80 mL/min, who are also receiving concomitant CYP3A4 inhibitors, the dosing interval should be increased to 150mg once daily. (For full details, see Table in the Separate document: current ‘Summary of product characteristics for maraviroc’)

Of note, the maraviroc dose does not vary according to concomitant nucleos(t)ide reverse transcriptase inhibitor use due to no significant interactions.

For commonly used non-nucleoside reverse transcriptase inhibitors (NNRTIs), protease inhibitors (PIs) and integrase strand transfer inhibitors (INSTIs), the dose of concomitant maraviroc is as follows:

Maraviroc 150mg BD

Darunavir/ritonavir, Atazanavir/ritonavir, Darunavir/cobicistat, Atazanavir/cobicistat, Elvitegravir/cobicistat

Maraviroc 300mg BD

Rilpivirine, Nevirapine

Raltegravir, Dolutegravir

Maraviroc 600mg BD

Efavirenz, Etravirine (in absence of a boosted PI) [note that Etravirine is only approved for use with a boosted protease inhibitor in the EU SPC]

8.2 Legal status of the drug

Maraviroc is licensed in the UK to be administered with other antiretroviral medications for treatment-experienced adults infected with only CCR5-tropic HIV-1.

8.3 Summary of Product Characteristics (SPC)

See separate RSI review document and the current version of 'Summary of Product Characteristics for Maraviroc'

8.4 Drug storage and supply

The trials pharmacy will maintain its own stock and will be responsible for ordering further IMP when necessary. The trials pharmacy will maintain accountability logs and ensure prescriptions are signed only by a medically-qualified doctor listed on the delegation log.

8.5 Preparation and labelling of Investigational Medicinal Product

IMP will be delivered to the Royal Free Hospital from the manufacturer where the QP process will be performed, including the labelling, release activity and QP certification. The labelled IMP will then be couriered to the clinical trials pharmacy. The first supply will be triggered once the first participant is screened.

8.6 Dosage schedules

Maraviroc will be taken by participants in Group A orally at a twice daily dosing schedule. The total quantity of drug will be determined according to interactions with other co-administered medications, including antiretrovirals, in line with SPC recommendations.

8.7 Dosage modifications

Modification of the dose of maraviroc will not be undertaken except where indicated due to potential drug-drug interactions with concomitant medications. Where a participant initiates a new medication or discontinues an existing medication, the investigator or his/her delegate will confirm whether or not a drug drug interaction could occur and hence any dose modification of MVC.

8.8 Known drug reactions and interaction with other therapies

Please see the current Summary of Product Characteristics for Maraviroc for known drug interactions

8.9 Concomitant medication

Please see the current Summary of Product Characteristics for Maraviroc for known drug interactions with respect to contraindicated medications.

8.10 Trial restrictions

For women of child bearing potential, contraception needs to be used for the duration of the study. This includes the following, according to the woman's preference and DDIs with concomitant medications:

- Intrauterine Device (IUD)

- Hormonal based contraception (pill, contraceptive injection, implant, IUS etc.)
- Double Barrier contraception (condom and occlusive cap e.g. diaphragm or cervical cap with spermicide)
- True abstinence

8.11 Assessment of compliance

Aim: To describe how compliance will be assessed

Participant adherence to MVC will be assessed through:

a) Self reporting of doses of MVC taken via a diary card
b) Pill counting at each visit by a pharmacist/research team member and recording of the number of pills returned

Participants will bring in all pill bottles at each study visit. The total number of IMP pills remaining at each visit will be counted and, then returned to the participant to take until the bottle is finished.

The percentage of IMP compliance for each participant will be calculated. Where this figure is <80%, this will lead to likely withdrawal from the study although this will be at the discretion of the PI. Where a discrepancy exists between self reported compliance and compliance identified via pill counting, any decision to withdraw the participant will rest with the study PI.

8.12 Name and description of each Non-Investigational Medicinal Product (NIMP)

All participants will be receiving an OBT at screen which may consist of any cART regimen which has achieved a sustained undetectable HIV VL.

9. PHARMACOVIGILANCE

9.1 Definitions

Term	Definition
**Adverse Event (AE)**	Any untoward medical occurrence in a participant to whom a medicinal product has been administered, including occurrences which are not necessarily caused by or related to that product.
**Adverse Reaction (AR)**	An untoward and unintended response in a participant to an investigational medicinal product which is related to any dose administered to that participant.<br>The phrase "response to an investigational medicinal product" means that a causal relationship between a trial medication and an AE is at least a reasonable possibility, i.e. the relationship cannot be ruled out.<br>All cases judged by either the reporting medically qualified professional or the Sponsor as having a reasonable suspected causal relationship to the trial medication qualify as adverse reactions.

**Serious Adverse Event (SAE)**	A serious adverse event is any untoward medical occurrence that:<br>• results in death<br>• is life-threatening<br>• requires inpatient hospitalisation or prolongation of existing hospitalisation<br>• results in persistent or significant disability/incapacity<br>• consists of a congenital anomaly or birth defect<br><br>Other ‘important medical events’ may also be considered serious if they jeopardise the participant or require an intervention to prevent one of the above consequences.<br><br>NOTE: The term "life-threatening" in the definition of "serious" refers to an event in which the participant was at risk of death at the time of the event; it does not refer to an event which hypothetically might have caused death if it were more severe.
**Serious Adverse Reaction (SAR)**	An adverse event that is both serious and, in the opinion of the reporting Investigator, believed with reasonable probability to be due to one of the trial treatments, based on the information provided.
**Suspected Unexpected Serious Adverse Reaction (SUSAR)**	A serious adverse reaction, the nature and severity of which is not consistent with the information about the medicinal product in question set out:<br>• in the case of a product with a marketing authorisation, in the summary of product characteristics (SmPC) for that product<br>• in the case of any other investigational medicinal product, in the investigator’s brochure (IB) relating to the trial in question

NB: to avoid confusion or misunderstanding of the difference between the terms “serious” and “severe”, the following note of clarification is provided: “Severe” is often used to describe intensity of a specific event, which may be of relatively minor medical significance. “Seriousness” is the regulatory definition supplied above. For example, a headache may be severe in intensity but would not be classified as serious unless it met one of the criteria for serious events.

9.2 Adverse Events and Toxicity Management

Adverse events observed by the Investigator, or reported by the subject, and any remedial action taken, will be recorded in the subject’s eCRF and should be verifiable in the subject’s notes throughout the study. The nature of each event, date of onset, duration and severity will be documented together with the Investigator’s opinion of the causal relationship to the treatment (unrelated, unlikely, possible, probable, and definite).

All subjects experiencing adverse events, whether considered associated with the use of the study medication or not, must be monitored until the symptoms subside and any clinically relevant changes in laboratory values have returned to baseline, or until there is a satisfactory explanation for the changes observed.

Procedures such as surgery should not be reported as adverse events. However, the medical condition for which the procedure was performed should be reported if it meets the definition of an adverse event. For example, an acute appendicitis that begins during the adverse event reporting period should be reported as the adverse event and the resulting appendectomy noted on the eCRF.

Planned procedures such as surgery planned prior to the subject's enrolment into the study need not be reported as adverse events if these are documented as planned at the screening visit.

Clinically significant changes in physical examination and blood safety profiles should also be recorded as adverse events.

An adverse event includes:

- An exacerbation of a pre-existing illness
- An increase in frequency or intensity of a pre-existing episodic event or condition
- A condition detected or diagnosed after trial medication administration even though it may have been present prior to the start of the trial
- Continuous persistent disease/symptoms present at baseline that worsen following the start of the trial

An adverse event does **NOT** include:

- Medical or surgical procedures (e.g. surgery, endoscopy, tooth extraction, transfusion); the condition that leads to the procedure is an AE.
- Pre-existing disease or conditions present at the start of the trial that do not worsen.
- Situations where an untoward medical occurrence has not occurred (e.g. hospitalisations for cosmetic elective surgery/social/convenience admissions).
- HIV-associated conditions or signs/symptoms associated with such conditions unless more severe than expected for the subject's condition.
- Overdose of either antiretroviral agents or concomitant medication without any signs or symptoms.
- A laboratory abnormality that is judged by the investigator not to be of clinical significance

Assessment of Intensity

Severity should be recorded and graded according to the AIDS Clinical Trial Group (ACTG) Grading Scale (Appendix 2).

All events deemed to be Grade 4 (potentially life threatening) according to the ACTG grading scale should be routinely reported as a serious adverse event. However there may be occasions where in the investigator's clinical judgement they do not consider the event to be life threatening; therefore they do not consider the event to meet the definition of an SAE. In these cases the investigator must document clearly in the participants source documentation that the Grade 4 event has been assessed and why in their clinical judgement they do not consider the event to be life threatening.

Assessment of Causality

The relationship to study drug of each adverse event will be assessed using the following definitions:

DEFINITE: distinct temporal relationship with drug treatment. Known reaction to agent or chemical group, or predicted by known pharmacology. Event cannot be explained by subject's clinical state or other factors.

PROBABLE: reasonable temporal relationship with drug treatment. Likely to be known reaction to agent or chemical group, or predicted by known pharmacology. Event cannot easily be explained by subject's clinical state or other factors.

POSSIBLE: reasonable temporal relationship with drug treatment. Event could be explained by subject's clinical state or other factors.

UNLIKELY: poor temporal relationship with drug treatment. Event easily explained by subject's clinical state or other factors.

UNRELATED: the event occurs prior to dosing. Event or intercurrent illness is due wholly to factors other than drug treatment.

9.3 Recording and reporting of AEs, ARs, SAEs, SARs AND SUSARs

All AEs, ARs, SAEs, SARs and SUSARs will be documented in the eCRF.

The adverse event reporting period will be from consent until the subject's final study visit. In addition, any untoward event that may occur subsequent to the reporting period that the Investigator assesses as possibly, probably or definitely related to the study drug medication will also be reported as an Adverse Reaction.

AEs, ARs, SAEs, , SARs and SUSARs may be directly observed, reported spontaneously by the subject or by questioning the subject at each study visit. These will be followed up until they are resolved or the

subject's participation in the study ends (i.e. until the final eCRF is completed for that subject). In addition, all serious adverse events assessed by the Investigator as possibly related to the investigational medication should continue to be followed even after the subject's participation in the study is over.

Such events should be followed until resolution, or until no further change can reasonably be expected. Deaths occurring more than 30 days after the final dose, which are considered to be unrelated to the study medication, should not be reported as a Serious Adverse Event.

SAEs should be reported immediately to the BSCTU.

All **SAEs** occurring from the time of **consent** until **30** days post cessation of trial treatment must be recorded on the SAE Form and emailed to the BSCTU immediately at BSCTUsafety@bsms.ac.uk, at least **within 24 hours** of the research staff becoming aware of the event.

For each **SAE** the following information will be collected:

- full details in medical terms and case description
- event duration (start and end dates, if applicable)
- action taken
- outcome
- seriousness criteria
- causality (i.e. relatedness to trial drug / investigation), in the opinion of the investigator
- whether the event would be considered expected or unexpected in relation to the Reference Safety Information.

Any change of condition or other follow-up information should be emailed to the Sponsor as soon as it is available or at least within 24 hours of the information becoming available. Events will be followed up until the event has resolved or a final outcome has been reached.

All SAEs assigned by the PI or delegate (or following central review by the CI) as both suspected to be related to IMP-treatment and unexpected will be classified as SUSARs and will be subject to expedited reporting to the Medicines and Healthcare Products Regulatory Agency (MHRA). The Sponsor will inform the MHRA and the REC of SUSARs within the required expedited reporting timescales.

See Appendix 3 for a Safety Reporting Flowchart.

The following are clinically important adverse reactions of moderate intensity or more occurring among patients receiving maraviroc at rates greater than rates in the comparator as set out in the SPC:

Tabulated list of adverse reactions (reference safety information)

The adverse reactions are listed by system organ class (SOC) and frequency. Within each frequency grouping, undesirable effects are presented in order of decreasing seriousness. Frequencies are defined as very common (≥ 1/10), common (≥ 1/100 to < 1/10), uncommon (≥1/1000 to <1/100), rare (≥1/10,000 to <1/1,000) not known (cannot be estimated from the available data). The adverse reactions and laboratory abnormalities presented below are not exposure adjusted.

**System Organ Class**	**Adverse reaction**	**Frequency**
Infections and infestations	Pneumonia, oesophageal candidiasis	uncommon
Neoplasm benign, malignant and unspecified (including cysts and polyps)	Bile duct cancer, diffuse large B-cell lymphoma, Hodgkin's disease, metastases to bone, metastases to liver, metastases to peritoneum, nasopharyngeal cancer, oesophageal carcinoma	rare
Blood and lymphatic system disorders	Anaemia	common
	Pancytopenia, granulocytopenia	rare
Metabolism and nutrition disorders	Anorexia	common
Psychiatric disorders	Depression, insomnia	common
Nervous system disorders	Seizures and seizure disorders	uncommon
Cardiac disorders	Angina pectoris	rare
Vascular disorders	Postural hypotension	uncommon
Gastrointestinal disorders	Abdominal pain, flatulence, nausea	common
Hepatobiliary disorders	Alanine aminotransferase increased, aspartate aminotransferase increased	common
	Hyperbilirubinaemia, gamma-glutamyltransferase increased	uncommon
	Hepatitis toxic, hepatic failure, hepatic cirrhosis, blood alkaline phosphatase increased	rare
	Hepatic failure with allergic features	very rare
Skin and subcutaneous tissue disorders	Rash	common
	Stevens-Johnson syndrome / Toxic epidermal necrolysis	rare / not known
Musculoskeletal and connective tissue disorders	Myositis, blood creatine phosphokinase increased	uncommon

	Muscle atrophy	rare
Renal and urinary disorders	Renal failure, proteinuria	uncommon
General disorders and administration site conditions	Asthenia	common

Cases of osteonecrosis have been reported, particularly in patients with generally acknowledged risk factors, advanced HIV disease or long-term exposure to combination antiretroviral therapy (cART). The frequency of this is unknown.

Delayed type hypersensitivity reactions, typically occurring within 2-6 weeks after start of therapy and including rash, fever, eosinophilia and liver reactions have been reported. Skin and liver reactions can occur as single events, or in combination.

In HIV infected patients with severe immune deficiency at the time of initiation of combination antiretroviral therapy, an inflammatory reaction to asymptomatic or residual opportunistic infections may arise. Autoimmune disorders (such as Graves' disease and autoimmune hepatitis) have also been reported; however, the reported time to onset is more variable and these events can occur many months after initiation of treatment.

Cases of syncope caused by postural hypotension have been reported.

For SUSARs, defined in section 9.1, the expedited reporting requirement will follow BSCTU SOP.

9.4 Responsibilities

"Principal Investigator (PI):

Checking for AEs and ARs when participants attend for treatment / follow-up.

1. Using medical judgement in assigning seriousness, causality and expectedness using the Reference Safety Information approved for the trial.
2. Ensuring that all SAEs and SARs (including SUSARs) are recorded and reported to the Sponsor within 24 hours of becoming aware of the event and provide further follow-up information as soon as available. Ensuring that SAEs and SARs (including SUSARs) are chased with Sponsor if a record of receipt is not received within 2 working days of initial reporting.
3. Ensuring that AEs and ARs are recorded and reported to the Sponsor in line with the requirements of the protocol.

Chief Investigator (CI) / delegate or independent clinical reviewer:

1. Clinical oversight of the safety of patients participating in the trial, including an ongoing review of the risk / benefit.
2. Using medical judgement in assigning seriousness, causality and expectedness of SAEs where it has not been possible to obtain local medical assessment.
3. Using medical judgement in assigning expectedness
4. Immediate review of all SUSARs.
5. Review of SAEs and SARs in accordance with the trial risk assessment and protocol as detailed in the Trial Monitoring Plan.
6. Assigning Medical Dictionary for Regulatory Activities (MedDRA) or Body System coding to all SAEs and SARs.
7. Preparing the clinical sections and final sign off of the Development Safety Update Report (DSUR).

Sponsor (as delegated to BSCTU):

1. Central data collection and verification of AEs, ARs, SAEs, SARs and SUSARs according to the trial protocol onto a MACRO database.
2. Reporting safety information to the CI, delegate or independent clinical reviewer for the ongoing assessment of the risk / benefit according to the Trial Monitoring Plan.
3. Reporting safety information to the independent oversight committees identified for the trial (Data Safety and Monitoring Board(DSMB) and / or Trial Steering Committee (TSC)) according to the Trial Monitoring Plan.
4. Expedited reporting of SUSARs to the Competent Authority (MHRA in UK) and REC within required timelines.
5. Notifying Investigators of SUSARs that occur within the trial.
6. Checking for (annually) and notifying PIs of updates to the Reference Safety Information for the trial.
7. Preparing standard tables and other relevant information for the DSUR in collaboration with the CI and ensuring timely submission to the MHRA and REC.

The role of the Trial Steering Committee (TSC) is to provide overall supervision for the HEPMARC trial on behalf of the Trial Sponsor and the Trial Funder and to ensure that the trial is conducted according to the guidelines for Good Clinical Practice (GCP), Research Governance Framework for Health and Social Care and all relevant regulations and local policies.

Data Safety and Monitoring Board (DSMB):

In accordance with the Trial Terms of Reference for the DSMB, periodically reviewing overall safety data to determine patterns and trends of events, or to identify safety issues, which would not be apparent on an individual case basis.

9.5 Notification of deaths

All deaths will be reported to the sponsor immediately irrespective of whether the death is related to disease progression, the IMP, or an unrelated event. When recording death, the event or condition that contributed to the fatal outcome will be recorded as a single medical concept, with death as an outcome of that event or condition.

9.6 Pregnancy reporting

- All pregnancies within the trial (either the trial participant or the participant's partner) will be reported to the BSCTU using the relevant Pregnancy Reporting Form within 24 hours of notification
- Pregnancy will not be considered an AE unless a negative or consequential outcome is recorded for the mother or child/foetus. If the outcome meets the serious criteria, this will be considered an SAE.
- Pregnancy subjects will be followed up by the HIV pregnancy service of the relevant site and reported to the antiretroviral pregnancy registry.

9.7 Overdose

- Overdoses will be reported to the sponsor immediately and this information will be placed on the deviation log)
- Overdose will be observed from patient comments.
- An overdose will constitute more than 1200mg of maraviroc per day
- There is no specific antidote for overdose with maraviroc. Treatment of overdose will depend on the amount of overdose and should consist of general supportive measures which may include keeping the patient in a supine position, careful assessment of patient vital signs, blood pressure and ECG. If indicated, elimination of unabsorbed active maraviroc should be achieved by emesis or gastric lavage. Administration of activated charcoal may also be used to aid in removal of unabsorbed active substance. Since maraviroc is moderately protein bound, dialysis may be beneficial in removal of this medicine. Further management will be as recommended by the national poisons centre.
- If an SAE is associated with the overdose, the overdose will be fully described in the SAE report form.

9.8 Reporting urgent safety measures

If any urgent safety measures are taken the CI/Sponsor shall immediately and in any event no later than 3 days from the date the measures are taken, give written notice to the MHRA and the relevant REC of the measures taken and the circumstances giving rise to those measures.

9.9 The type and duration of the follow-up of subjects after adverse events.

The type and duration of follow up of subjects after adverse events will depend on the seriousness of the event and the specific nature of the event to be determined at the discretion of the chief investigator.

Any SUSAR related to the IMP will need to be reported to the Sponsor irrespective of how long after IMP administration the reaction has occurred.

9.10 Development safety update reports

The BSCTU will submit DSURs once a year throughout the clinical trial, or on request to the Competent Authority (MHRA in the UK), Ethics Committee and NHS Trusts.

10 STATISTICS AND DATA ANALYSIS

10.1 Sample size calculation

This is a pilot study to evaluate the feasibility and potential efficacy of addition of maraviroc to optimised background combination antiretroviral therapy and therefore no formal sample size calculation has been conducted. Results may be used to estimate the variability of the treatment effect of MVC on the ELF score which in turn may inform the sample size calculation for a larger placebo-controlled RCT.

In a previous biopsy study, a unit increase of 1 in the ELF score was associated with a 2.5-fold increased risk of a liver-related event (adjusted for age and stage of fibrosis) and therefore a unit increase of 1 is deemed to represent a clinically important entity [24]. Assuming the SD of the ELF score is 1.12, with 20 patients in each group for the analysis, a difference in ELF of 1 point can be estimated with a 95% confidence interval from 0.6 to 1.4. Assuming an attrition rate of 33%, a target of 30 individuals will be recruited per group [23].

A t-method will be used to estimate the difference in ELF scores, together with 95% confidence intervals, between the two groups. P-values will not be presented.

10.2 Planned recruitment rate

An estimated 3 individuals will be recruited per month over a 21 month period. This takes into account the following factors:

- The estimated prevalence of NAFLD amongst HIV-infected cohorts is predicted to be 30%.Although a large proportion of these individuals are likely to remain undiagnosed in the absence of a NAFLD screening programme, recruitment at several sites with > 5000 HIV-infected patients overall is expected to yield at least 200 who have been diagnosed with NAFLD on previous imaging as part of routine clinical care
- All HIV-infected patients are expected to attend a minimum of two medical appointments per year. For a minimum of 5000 HIV-infected patients, a monthly target of 4-5 is predicted to be feasible, allowing for 1-2 screen failures per month leaving 3-4 recruited per month.

10.3 Statistical analysis plan

10.3.1 Summary of baseline data and flow of patients

Baseline comparability between the randomised groups (maraviroc-allocated, versus non-maraviroc allocated,) will be described by considering the variables below.

For categorical data, % will be presented.

For continuous data, mean or median will be presented for variables following a normal and non-normal distribution, respectively, in addition to the interquartile range (IQR).

Age (IQR)

% male

% each ethnicity

Duration of HIV infection (IQR)

Nadir CD4 count (IQR)

Baseline CD4 count (IQR)

% with undetectable HIV VL

% receiving PI-based cART vs non-PI based cART

% receiving concomitant lipid lowering therapy

BMI (IQR)

Waist circumference (IQR)

Weight (IQR)

BP (IQR)

Fasting glucose (IQR)

HbA1c (IQR)

Bilirubin (IQR)

ALT (IQR)

Fasting TG (IQR)

Fasting LDL (IQR)

Fasting HDL (IQR)

Fasting total cholesterol (IQR)

Fasting HDL:chol ratio (IQR)

Baseline ELF score (IQR)

Baseline Fibroscan stiffness result (IQR)

Baseline Fibroscan CAP score (IQR)
% with a CT liver:spleen attenuation ratio <1.0
Baseline diet score (IQR)

See Figure 1 for the CONSORT flow diagram for this study.

CONSORT
TRANSPARENT REPORTING of TRIALS

Figure 1. HEPMARC Flow Diagram

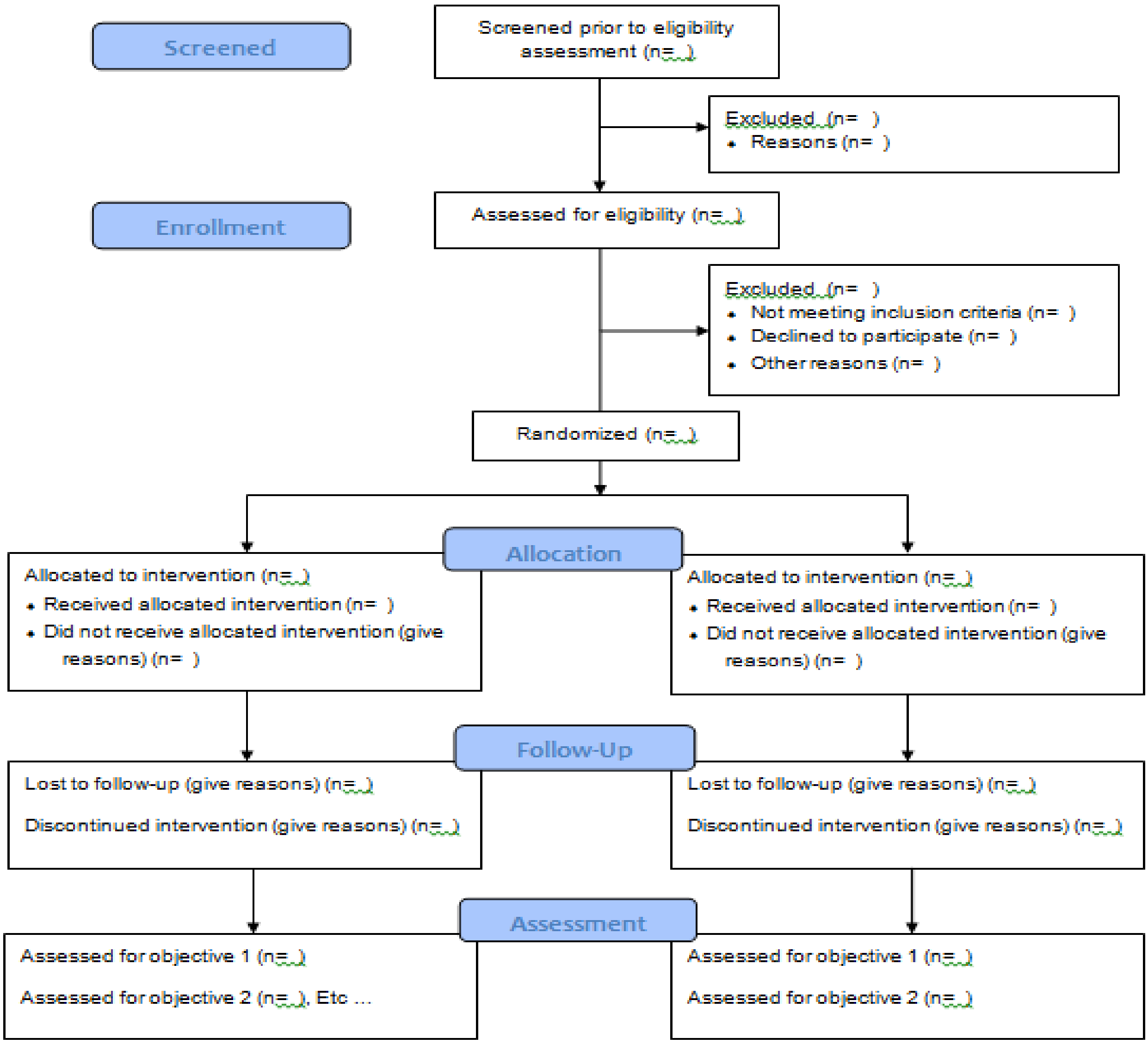

Citation: Eldridge SM, Chan CL, Campbell MJ, Bond CM, Hopewell S, Thabane L, et al. CONSORT 2010 statement: extension to randomised pilot and feasibility trials. BMJ. 2016;355.

HEPMARC Consort Flow Diagram v1.0

Figure 1: CONSORT flow diagram for the HEPMARC study.

10.3.2 Feasibility Outcome analyses

All analyses will be performed on available cases according to intention to treat (ITT) principles. Therefore, all individuals randomised within the study (including those subsequently withdrawn) will be included in outcome analyses.

The following analyses will be presented:

a) Proportion of eligible individuals approached who are successfully recruited
It is envisaged that a recruitment rate of at least 50% will achieved. This takes into account the likelihood that a proportion of potential recruits will decline particularly owing to the requirement for twice daily pill taking.

b) Monthly participant recruitment rate. This is expected to be a minimum of at least 2 participants consistently per month, with an average of 3-4.

c) Participant retention in the study at 48 and 96 weeks in the maraviroc and non-maraviroc assigned groups. This is expected to be at least 65% (cf 35% discontinuation in the MOTIVATE study [9]).

d) Proportion of participants for whom there is missing data at 48 and 96 weeks in the maraviroc and non-maraviroc assigned groups. This is expected to be less than 20% for all participants.

e) Proportion of participants reporting adverse events at 48 and 96 weeks in the maraviroc and non-maraviroc assigned groups. This is expected to be less than 10% [18].

f) Level of self-reported adherence to the study drug at 48 and 96 weeks in those allocated to the maraviroc group. This is expected to be greater than 90%.

10.3.3 Secondary outcome analysis

The analysis of the following secondary outcomes will be presented as the estimated difference and 95% confidence interval between groups using a t-method from baseline to 48 and 96 weeks.

The mean change in ELF score

Mean change in the Fibroscan stiffness

Mean change in Fibroscan CAP score

Mean change in fasting lipids (HDL:cholesterol ratio, HDL, LDL, TG), ALT, fasting glucose, HbA1c, in the maraviroc and non-maraviroc groups

Mean change in the BMI, waist circumference and weight

Mean change in the CD4 count

Mean scores for the CLDQ:NAFLD , SF-36 and WAIP:SAH questionnaires

Differences in the following measures from baseline to 48 and 96 weeks will be presented exact 95% binomial confidence intervals:

% with HIV viral load not detected (48 and 96 weeks)

% with CT liver:spleen attenuation ratio <1.0 (96 weeks only)

10. 4 Procedure(s) to account for missing or spurious data

In the event of missing data, only available data will be included in the analyses.

11 DATA HANDLING

11.1 Data collection tools and source document identification

An Electronic Data management system MACRO^{TM} will be used in this study.

All information will be recorded in source data and documentation that will be filed in patients' notes.

A source data worksheet will be created to capture all the relevant information and will be filed as source documentation in the patient notes. A source data agreement will be completed prior to recruitment commencing to ensure all parties are aware which documents constitute source data.

Data from the source will be entered onto the electronic case report form (eCRF) on the Web-based MACRO^{TM} electronic data capture system.

11.2 Data handling and record keeping

MACRO^{TM} will be used for data entry. Data entered will be checked by the Data Manager in accordance with the Data Management Plan and queries raised to the clinical sites via MACRO when appropriate. Clinical sites will be responsible for the entry of data into the eCRF. Patient data will be entered using study number only and no patient identifiable data will be seen by the data management team. Analysis will be the responsibility of the CTU statisticians.

11.3 Access to Data

Direct access will be granted to authorised representatives from the Sponsor, host institution and the regulatory authorities when appropriate to permit trial-related monitoring, audits and inspections.

11.4 Archiving

Archiving will be the responsibility of the Sponsor and all documentation will be archived for 25 years, with the exception of the health records which will be kept in accordance with UK law and local policy. The archiving will be sent off site to Iron Mountain. The sponsor named archivist will be responsible for ensuring the documentation is prepared and sent to Iron Mountain in line with the relevant Sponsor SOP.

12 MONITORING, AUDIT & INSPECTION

Aim: to describe the procedures for monitoring audit and inspection

The protocol should state:

- A Trial Monitoring Plan will be developed and agreed by the Trial Management Group (TMG) based on the trial risk assessment which may include on site monitoring
- The procedures and anticipated frequency for monitoring. A monitoring plan will be developed based on a detailed risk assessment.
- If monitoring procedures are detailed elsewhere (e.g., monitoring manual), where the full details can be obtained
- The degree of independence from the trial investigators and sponsor of the monitoring personnel
- The processes reviewed can relate to participant enrolment, consent, eligibility, and allocation to trial groups; adherence to trial interventions and policies to protect participants, including reporting of harm and completeness, accuracy, and timeliness of data collection
- Monitoring can be done by exploring the trial dataset or performing site visits
- Any obligations that will be expected of sites to assist the sponsor in monitoring the study. These may include hosting site visits, providing information for remote monitoring, or putting procedures in place to monitor the study internally
- Monitoring might be initially conducted across all sites, and subsequently conducted using a risk based approach that focuses, for example, on sites that have the highest enrolment rates, large numbers of withdrawals, or atypical (low or high) numbers of reported adverse events.

13 ETHICAL AND REGULATORY CONSIDERATIONS

13.1 Research Ethics Committee (REC) and Health Research Authority review & reports

- Before the start of the trial, a favourable opinion will be sought from the REC as well as approval from the HRA for the trial protocol, informed consent forms and other relevant study documents

  Substantial amendments that require review by REC, MHRA and/or NHS R&D will not be implemented until the REC grants a favourable opinion for the study

- All correspondence with the REC and the HRA will be retained in the Trial Master File/Investigator Site File
- An annual progress report (APR) will be submitted to the REC within 30 days of the anniversary date on which the favourable opinion was given, and annually until the trial is declared ended
- The Chief Investigator will produce the annual reports as required.
- The Chief Investigator will notify the REC of the end of the study within the required timelines
- If the study is ended prematurely, the Chief Investigator will notify the REC, including the reasons for the premature termination within 15 days of this decision
- Within one year after the end of the study, the Chief Investigator will submit a final report with the results, including any publications/abstracts, to the REC

13.2 Peer review

Peer review of the protocol has been undertaken as part of sponsor approval process as well as part of the funder, which approved funding following submission of the proposal through an open competitive process.

13.3 Regulatory Compliance

- The trial will not commence until a Clinical Trial Authorisation (CTA) is obtained from the MHRA
- the protocol and trial conduct will comply with the Medicines for Human Use (Clinical Trials) Regulations 2004 and any relevant amendments
- Before any site can enrol patients into the trial, the Chief Investigator/Principal Investigator or designee will apply for Confirmation of Capacity and Capability from the site's Research & Development (R&D) department
- For any amendment that will potentially affect a site's Confirmation of Capacity and Capability, the Chief Investigator/ Principal Investigator or designee will confirm with that site's R&D department that NHS permission is ongoing (note that both substantial amendments, and amendments considered to be non-substantial for the purposes of REC and/or MHRA may still need to be notified to NHS R&D depending upon the HRA categorisation of the amendment).

13.4 Protocol compliance

Protocol deviations, non-compliances, or breaches are departures from the approved protocol.

- Prospective, planned deviations or waivers to the protocol are not allowed under the UK regulations on Clinical Trials and must not be used e.g. it is not acceptable to enrol a subject if they do not meet the eligibility criteria or restrictions specified in the trial protocol
- Accidental protocol deviations can happen at any time. They must be adequately documented on the relevant forms and reported to the Chief Investigator and Sponsor immediately.
- Deviations from the protocol which are found to frequently recur are not acceptable, will require immediate action and could potentially be classified as a serious breach.

13.5 Notification of Serious Breaches to GCP and/or the protocol

A “serious breach” is a breach which is likely to effect to a significant degree –

(a) the safety or physical or mental integrity of the subjects of the trial; or
(b) the scientific value of the trial

- The sponsor will be notified immediately of any case where the above definition applies during the trial conduct phase
- The sponsor of a clinical trial will notify the licensing authority in writing of any serious breach of

  (a) the conditions and principles of GCP in connection with that trial; or
  (b) the protocol relating to that trial, as amended from time to time, within 7 days of becoming aware of that breach

13.6 Data protection and patient confidentiality

All investigators and trial site staff will comply with the requirements of the Data Protection Act 1998 with regards to the collection, storage, processing and disclosure of personal information and will uphold the Act’s core principles.

- Personal information will be collected, kept secure, and maintained. This will involve:
  - the creation of coded, depersonalised data where the participant’s identifying information is replaced by an unrelated sequence of characters
  - secure maintenance of the data and the linking code in separate locations using encrypted digital files within password protected folders and storage media
  - limiting access to the minimum number of individuals necessary for quality control, audit, and analysis
- The confidentiality of data will be preserved when the data are transmitted to sponsors and co-investigators by using only pseudynomised codes rather than personal identifiable information

- Data will be stored for 25 years
- The PI at site is the data custodian.

13.7 Financial and other competing interests for the chief investigator, PI at site and committee members for the overall trial management

The Chief Investigator does not have any competing interests or conflicts of interest. The members of the data safety committee are independent and not members of the direct study or protocol team.

13.8 Indemnity

Full NHS Indemnity will be provided by the Sponsor.

13.9 Amendments

Any changes to the protocol will need sponsor approval prior to be submitted to the Ethics and/or regulatory authorities. It is the sponsor responsibility to assess whether the amendment is substantial or non-substantial. Once the sponsor has made and documented the decision it is the responsibility of the CI to communicate this to the relevant stakeholders but they may delegate this task to a member of the CTU trial management team.

13.10 Post trial care

Post trial, all individuals will be provided with standard of care interventions indicated for the treatment of NAFLD in line with current NICE guidelines. In addition, all participants will be offered ongoing follow-up within appropriate services at the local site.

All participating individuals will be advised, as part of the informed consent process, that maraviroc would be discontinued post-trial. Where individual patients and/or their treating clinicians request the continuation of maraviroc post trial, this request will be considered on a case by case basis by the HIV multidisciplinary team at each NHS site. Although the primary endpoint of the current study does not include efficacy of the drug, a substantial improvement in hepatic fibrosis markers in individual patients is likely to strengthen the case for continuation of drug, which will be made based on its need within the overall HIV treatment regimen.

In addition, any beneficial effect identified in the trial may inform a change in relevant guidelines as well as the establishment of a larger study on the effect of maraviroc in NAFLD in PLWH. This in turn may lead to a change to national recommendations with the possibility that maraviroc could be prescribed to them through existing NHS funding schemes.

13.11 Access to the final trial dataset

Only members of the direct study team will have access to the final dataset. Any requests for data will need to be approved by the Chief Investigator.

14 DISSEMINATION POLICY

14.1 Dissemination policy

Results will be presented at both national and international meetings eg the British HIV Association and the American Association for the Study of Liver Disease.

Findings will also be written up for publication in target journals targeting audiences with interests in liver disease and HIV such as *Journal of Hepatology* and *AIDS*, respectively.

A newsletter for study participants will be provided at the end of the study to summarise findings.

Any member of the study team wanting to use the data for publication will need approval from the Chief Investigator.

14.2 Authorship eligibility guidelines and any intended use of professional writers

In accordance with the BSUH SOP on authorship eligibility, all authors of the final trial report will have contributed significantly to the design, execution, analysis and/or writing up of the study.

15 REFERENCES

1. Vuille-Lessard E, Lebouche B, Lennox L, Routy JP, Costiniuk CT, Pexos C, et al. Nonalcoholic fatty liver disease diagnosed by transient elastography with controlled attenuation parameter in unselected HIV monoinfected patients. Aids. 2016;30(17):2635-43. doi: 10.1097/QAD.0000000000001241. PubMed PMID: 27603289.
2. Maurice JB, Patel A, Scott AJ, Patel K, Thursz M, Lemoine M. Prevalence and risk factors of non-alcoholic fatty liver disease in HIV-monoinfection: a systematic review and meta-analysis. Aids. 2017. doi: 10.1097/QAD.0000000000001504. PubMed PMID: 28398960.
3. Tafesh Z, Verna, EC. Managing nonalcoholic fatty liver disease in patients living with HIV. Current opinion in infectious diseases. 2017;30:12-20.
4. NICE. https://www.nice.org.uk/guidance/NG49/chapter/Recommendations#lifestyle-modifications-for-nafld 2016 [2017].
5. Schwabe RF, Bataller R, Brenner DA. Human hepatic stellate cells express CCR5 and RANTES to induce proliferation and migration. American journal of physiology Gastrointestinal and liver physiology. 2003;285(5):G949-58. doi: 10.1152/ajpgi.00215.2003. PubMed PMID: 12829440.
6. Seki E, De Minicis S, Gwak GY, Kluwe J, Inokuchi S, Bursill CA, et al. CCR1 and CCR5 promote hepatic fibrosis in mice. The Journal of clinical investigation. 2009;119(7):1858-70. PubMed PMID: 19603542; PubMed Central PMCID: PMC2701864.
7. Berres ML, Koenen RR, Rueland A, Zaldivar MM, Heinrichs D, Sahin H, et al. Antagonism of the chemokine Ccl5 ameliorates experimental liver fibrosis in mice. The Journal of clinical investigation. 2010;120(11):4129-40. doi: 10.1172/JCI41732. PubMed PMID: 20978355; PubMed Central PMCID: PMC2964968.
8. Barashi N, Weiss ID, Wald O, Wald H, Beider K, Abraham M, et al. Inflammation-induced hepatocellular carcinoma is dependent on CCR5 in mice. Hepatology. 2013;58(3):1021-30. doi: 10.1002/hep.26403. PubMed PMID: 23526353.
9. Gulick RM, Lalezari J, Goodrich J, Clumeck N, DeJesus E, Horban A, et al. Maraviroc for previously treated patients with R5 HIV-1 infection. The New England journal of medicine. 2008;359(14):1429-41. doi: 10.1056/NEJMoa0803152. PubMed PMID: 18832244; PubMed Central PMCID: PMC3078519.
10. Cooper DA, Heera J, Goodrich J, Tawadrous M, Saag M, Dejesus E, et al. Maraviroc versus efavirenz, both in combination with zidovudine-lamivudine, for the treatment of antiretroviral-naive subjects with CCR5-

tropic HIV-1 infection. The Journal of infectious diseases. 2010;201(6):803-13. doi: 10.1086/650697. PubMed PMID: 20151839.
11. Martin-Blondel G, Brassat D, Bauer J, Lassmann H, Liblau RS. CCR5 blockade for neuroinflammatory diseases--beyond control of HIV. Nature reviews Neurology. 2016;12(2):95-105. doi: 10.1038/nrneurol.2015.248. PubMed PMID: 26782333.
12. Gonzalez EO, Boix V, Deltoro MG, Aldeguer JL, Portilla J, Montero M, et al. The effects of Maraviroc on liver fibrosis in HIV/HCV co-infected patients. J Int AIDS Soc. 2014;17(4 Suppl 3):19643. doi: 10.7448/IAS.17.4.19643. PubMed PMID: 25394147; PubMed Central PMCID: PMC4224825.
13. Blanco JR, Ochoa-Callejero L. Off-label use of maraviroc in clinical practice. Expert review of anti-infective therapy. 2016;14(1):5-8. doi: 10.1586/14787210.2016.1100535. PubMed PMID: 26509356.
14. Rockstroh JK, Soriano V, Plonski F, Bansal M, Fatkenheuer G, Small CB, et al. Hepatic safety in subjects with HIV-1 and hepatitis C and/or B virus: a randomized, double-blind study of maraviroc versus placebo in combination with antiretroviral agents. HIV clinical trials. 2015;16(2):72-80. doi: 10.1179/1528433614Z.0000000011. PubMed PMID: 25923596.
15. Martini S, Perna A, Lucariello A, Macera M, Carleo MA, Guerra G, et al., editors. Effects of treatment with maraviroc - a CCR5 inhibitor on human hepatic stellate cells. Conference on Retroviruses and Opportunistic Infections; 2017 13-16 February; Seattle.
16. Perez-Martinez L, Perez-Matute P, Aguilera-Lizarraga J, Rubio-Mediavilla S, Narro J, Recio E, et al. Maraviroc, a CCR5 antagonist, ameliorates the development of hepatic steatosis in a mouse model of non-alcoholic fatty liver disease (NAFLD). The Journal of antimicrobial chemotherapy. 2014;69(7):1903-10. doi: 10.1093/jac/dku071. PubMed PMID: 24651825.
17. Nasta P, Gatti F, Borghi F, Chiari E, Paderni A, Carosi G. Liver stiffness (LS) change in HIV-hepatitis C (HCV) coinfected patients treated with CCR5 inhibitor based antiretroviral therapy. 51st Interscience Conference on Antimicrobial Agents and Chemotherapy; 17-20 September; Chicago2011.
18. Thompson M, Saag M, DeJesus E, Gathe J, Lalezari J, Landay A, et al. A 48-week randomized phase 2b study evaluating cenicriviroc versus efavirenz in treatment-naive HIV-infected adults with C-C chemokine receptor type 5-tropic virus Aids. 2016;30:869-78.
19. Friedman S, Sanyal A, Goodman Z, Lefebvre E, Gottwald M, Fischer L, et al. Efficacy and safety study of cenicriviroc for the treatment of non-alcoholic steatohepatitis in adult subjects with liver fibrosis: CENTAUR Phase 2b study design. Contemporary clinical trials. 2016;47:356-65. doi: 10.1016/j.cct.2016.02.012. PubMed PMID: 26944023.
20. Review T, LaBrecque DR, Abbas Z, Anania F, Ferenci P, Khan AG, et al. World Gastroenterology Organisation global guidelines: Nonalcoholic fatty liver disease and nonalcoholic steatohepatitis. Journal of clinical gastroenterology. 2014;48(6):467-73. doi: 10.1097/MCG.0000000000000116. PubMed PMID: 24921212.
21. Zeb I, Li D, Nasir K, Katz R, Larijani VN, Budoff MJ. Computed tomography scans in the evaluation of fatty liver disease in a population based study: the multi-ethnic study of atherosclerosis. Academic radiology. 2012;19(7):811-8. doi: 10.1016/j.acra.2012.02.022. PubMed PMID: 22521729; PubMed Central PMCID: PMC3377794.
22. Dongiovanni P, Petta S, Mannisto V, Mancina RM, Pipitone R, Karja V, et al. Statin use and non-alcoholic steatohepatitis in at risk individuals. Journal of hepatology. 2015;63(3):705-12. doi: 10.1016/j.jhep.2015.05.006. PubMed PMID: 25980762.
23. Martinez-Gonzalez MA, Fernandez-Jarne E, Serrano-Martinez M, Wright M, Gomez-Gracia E. Development of a short dietary intake questionnaire for the quantitative estimation of adherence to a cardioprotective Mediterranean diet. European journal of clinical nutrition. 2004;58(11):1550-2. doi: 10.1038/sj.ejcn.1602004. PubMed PMID: 15162136.
24. Irvine KM, Wockner LF, Shanker M, Fagan KJ, Horsfall LU, Fletcher LM, et al. The Enhanced liver fibrosis score is associated with clinical outcomes and disease progression in patients with chronic liver disease. Liver international : official journal of the International Association for the Study of the Liver. 2016;36(3):370-7. doi: 10.1111/liv.12896. PubMed PMID: 26104018.

25. Lin EC. Radiation risk from medical imaging. Mayo Clinic proceedings. 2010;85(12):1142-6; quiz 6. doi: 10.4065/mcp.2010.0260. PubMed PMID: 21123642; PubMed Central PMCID: PMC2996147.

16. APPENDICES

16.1 Appendix 1-Risk

Risks associated with trial interventions

☐ LOW ≡ Comparable to the risk of standard medical care

☒ MODERATE ≡ Somewhat higher than the risk of standard medical care

☐ HIGH ≡ Markedly higher than the risk of standard medical care

Justification: Briefly justify the risk category selected and your conclusions below (where the table is completed in detail the detail need not be repeated, however a summary should be given):

This study is a phase IV study in which participants are unlikely to undergo any risks additional to those that they would undergo as part of routine clinical care. Maraviroc has been proven to be a well tolerated antiretroviral with a low risk of toxicity.

The intervention of two CT scans (liver and spleen) represents a small additional risk with exposure to ionising radiation and therefore an increase in the risk of developing cancer. However, this is very small with the dose of radiation for each CT equivalent to 3 years of natural background exposure [25]. Additionally, the CT scans will be optional.

The interventions of venepuncture, hepatic ultrasound and Fibroscan are not envisaged to cause any additional harm to participants.

What are the key risks related to therapeutic interventions you plan to monitor in this trial?		How will these risks be minimised?		
IMP/Intervention	Body system/Hazard	Activity	Frequency	Comments
Maraviroc	Cardiovascular disease	Exclude patients with severe known CVD		
CT scans	Risk of cancer	Optional scans	BL and 96 wk	

DSMB will review results on an interim basis and will have the authority to stop the study early in the unlikely event of a safety signal being identified.

16.2 Appendix 2 AIDS Clinical Trial Group (ACTG) Grading Scale

DAIDS GRADING SCALE

DIVISION OF AIDS TABLE FOR GRADING THE SEVERITY OF
ADULT AND PEDIATRIC ADVERSE EVENTS
PUBLISH DATE: DECEMBER, 2004

Quick Reference

The Division of AIDS Table for Grading the Severity of Adult and Pediatric Adverse Events ("DAIDS AE grading table") is a descriptive terminology which can be utilized for Adverse Event (AE) reporting. A grading (severity) scale is provided for each AE term.

General Instructions

Estimating Severity Grade

If the need arises to grade a clinical AE that is not identified in the DAIDS AE grading table, use the category "Estimating Severity Grade". For AEs that are not listed in the table but will be collected systematically for a study/trial, protocol teams are highly encouraged to define study specific severity scales within the protocol or an appendix to the protocol. (Please see "Template Wording for the Expedited Adverse Event Reporting Section of DAIDS-sponsored Protocols".) This is particularly important for laboratory values because the "Estimating Severity Grade" category only applies to clinical symptoms.

Grading Adult and Pediatric AEs

The DAIDS AE grading table includes parameters for grading both Adult and Pediatric AEs. When a single set of parameters is not appropriate for grading specific types of AEs for both Adult and Pediatric populations, separate sets of parameters for Adult and/or Pediatric populations (with specified respective age ranges) are given in the table. If there is no distinction in the table between Adult and Pediatric values for a type of AE, then the single set of parameters listed is to be used for grading the severity of both Adult and Pediatric events of that type.

Determining Severity Grade

If the severity of an AE could fall under either one of two grades (e.g., the severity of an AE could be either Grade 2 or Grade 3), select the higher of the two grades for the AE.

Definitions

Basic Self-care Functions	Adult

	Activities such as bathing, dressing, toileting, transfer/movement, continence, and feeding.<br>Young Children<br>Activities that are age and culturally appropriate (e.g., feeding self with culturally appropriate eating implement).
LLN	Lower limit of normal
Medical Intervention	Use of pharmacologic or biologic agent(s) for treatment of an AE.
NA	Not Applicable
Operative Intervention	Surgical OR other invasive mechanical procedures.
ULN	Upper limit of normal
Usual Social & Functional Activities	Adult<br>Adaptive tasks and desirable activities, such as going to work, shopping, cooking, use of transportation, pursuing a hobby, etc.<br>Young Children<br>Activities that are age and culturally appropriate (e.g., social interactions, play activities, learning tasks, etc.).

CLINICAL				
**PARAMETER**	**GRADE 1 MILD**	**GRADE 2 MODERATE**	**GRADE 3 SEVERE**	**GRADE 4 POTENTIALLY LIFE-THREATENING**
**ESTIMATING SEVERITY GRADE**				
Clinical adverse event NOT identified elsewhere in this DAIDS AE grading table	Symptoms causing no or minimal interference with usual social & functional activities	Symptoms causing greater than minimal interference with usual social & functional activities	Symptoms causing inability to perform usual social & functional activities	Symptoms causing inability to perform basic self-care functions OR Medical or operative intervention indicated to prevent permanent impairment, persistent disability, or death
**SYSTEMIC**				
Acute systemic allergic reaction	Localized urticaria (wheals) with no medical intervention indicated	Localized urticaria with medical intervention indicated OR Mild angioedema with no medical intervention indicated	Generalized urticaria OR Angioedema with medical intervention indicated OR Symptomatic mild bronchospasm	Acute anaphylaxis OR Life-threatening bronchospasm OR laryngeal edema
Chills	Symptoms causing no or minimal interference with usual social & functional activities	Symptoms causing greater than minimal interference with usual social & functional activities	Symptoms causing inability to perform usual social & functional activities	NA

Fatigue<br>Malaise	Symptoms causing no or minimal interference with usual social & functional activities	Symptoms causing greater than minimal interference with usual social & functional activities	Symptoms causing inability to perform usual social & functional activities	Incapacitating fatigue/ malaise symptoms causing inability to perform basic self-care functions
Fever (nonaxillary)	37.7 – 38.6°C	38.7 – 39.3°C	39.4 – 40.5°C	> 40.5°C
Pain (indicate body site)<br>DO NOT use for pain due to injection (See Injection Site Reactions: Injection site pain)<br>See also Headache, Arthralgia, and Myalgia	Pain causing no or minimal interference with usual social & functional activities	Pain causing greater than minimal interference with usual social & functional activities	Pain causing inability to perform usual social & functional activities	Disabling pain causing inability to perform basic self-care functions OR Hospitalization (other than emergency room visit) indicated

Basic Self-care Functions – Adult: Activities such as bathing, dressing, toileting, transfer/movement, continence, and feeding.

Basic Self-care Functions – Young Children: Activities that are age and culturally appropriate (e.g., feeding self with culturally appropriate eating implement).

Usual Social & Functional Activities – Adult: Adaptive tasks and desirable activities, such as going to work, shopping, cooking, use of transportation, pursuing a hobby, etc.

Usual Social & Functional Activities – Young Children: Activities that are age and culturally appropriate (e.g., social interactions, play activities, learning tasks, etc.).

CLINICAL				
PARAMETER	GRADE 1<br>MILD	GRADE 2<br>MODERATE	GRADE 3<br>SEVERE	GRADE 4<br>POTENTIALLY LIFE-THREATENING
Unintentional weight loss	***NA***	***5 – 9% loss in body weight from baseline***	***10 – 19% loss in body weight from baseline***	***≥20% loss in body weight from baseline OR Aggressive intervention indicated [e.g., tube feeding or total parenteral nutrition (TPN)]***
INFECTION				
Infection (any other than HIV infection)	***Localized, no systemic antimicrobial treatment indicated AND Symptoms causing no or minimal interference with usual social & functional activities***	***Systemic antimicrobial treatment indicated OR Symptoms causing greater than minimal interference with usual social & functional activities***	***Systemic antimicrobial treatment indicated AND Symptoms causing inability to perform usual social & functional activities OR Operative intervention (other than simple incision and drainage) indicated***	***Life-threatening consequences (e.g., septic shock)***
INJECTION SITE REACTIONS				

Injection site pain (pain without touching) ***Or*** ***Tenderness (pain when area is touched)***	***Pain/tenderness causing no or minimal limitation of use of limb***	***Pain/tenderness limiting use of limb OR Pain/tenderness causing greater than minimal interference with usual social & functional activities***	***Pain/tenderness causing inability to perform usual social & functional activities***	***Pain/tenderness causing inability to perform basic self-care function OR Hospitalization (other than emergency room visit) indicated for management of pain/tenderness***
Injection site reaction (localized)				
Adult > 15 years	***Erythema OR Induration*** ***of 5x5 cm – 9x9 cm*** ***(or 25 cm² – 81cm²)***	***Erythema OR Induration OR Edema*** ***> 9 cm any diameter (or > 81 cm²)***	***Ulceration OR Secondary infection OR Phlebitis OR Sterile abscess OR Drainage***	***Necrosis (involving dermis and deeper tissue)***
Pediatric ≤ 15 years	***Erythema OR Induration OR Edema present but ≤ 2.5 cm diameter***	***Erythema OR Induration OR Edema > 2.5 cm diameter but < 50% surface area of the extremity segment (e.g., upper arm/thigh)***	***Erythema OR Induration OR Edema involving ≥ 50% surface area of the extremity segment (e.g., upper arm/thigh) OR Ulceration OR Secondary infection OR Phlebitis OR Sterile abscess OR Drainage***	***Necrosis (involving dermis and deeper tissue)***

Basic Self-care Functions – Adult: Activities such as bathing, dressing, toileting, transfer/movement, continence, and feeding.

Basic Self-care Functions – Young Children: Activities that are age and culturally appropriate (e.g., feeding self with culturally appropriate eating implement).

Usual Social & Functional Activities – Adult: Adaptive tasks and desirable activities, such as going to work, shopping, cooking, use of transportation, pursuing a hobby, etc.

Usual Social & Functional Activities – Young Children: Activities that are age and culturally appropriate (e.g., social interactions, play activities, learning tasks, etc.).

CLINICAL				
**PARAMETER**	**GRADE 1**<br>**MILD**	**GRADE 2**<br>**MODERATE**	**GRADE 3**<br>**SEVERE**	**GRADE 4**<br>**POTENTIALLY LIFE-THREATENING**
Pruritis associated with injection<br>***See also Skin: Pruritis (itching - no skin lesions)***	***Itching localized to injection site AND Relieved spontaneously or with < 48 hours treatment***	***Itching beyond the injection site but not generalized OR Itching localized to injection site requiring ≥ 48 hours treatment***	***Generalized itching causing inability to perform usual social & functional activities***	***NA***
SKIN – DERMATOLOGICAL				
Alopecia	***Thinning detectable by study participant (or by caregiver for young children and disabled adults)***	***Thinning or patchy hair loss detectable by health care provider***	***Complete hair loss***	***NA***
Cutaneous reaction – rash	***Localized macular rash***	***Diffuse macular, maculopapular, or morbilliform rash OR***<br>***Target lesions***	***Diffuse macular, maculopapular, or morbilliform rash with vesicles or limited number of bullae OR Superficial ulcerations of mucous membrane limited to one site***	***Extensive or generalized bullous lesions OR Stevens-Johnson syndrome OR Ulceration of mucous membrane involving two or more distinct mucosal sites OR Toxic epidermal necrolysis (TEN)***
Hyperpigmentation	***Slight or localized***	***Marked or generalized***	***NA***	***NA***
Hypopigmentation	***Slight or localized***	***Marked or generalized***	***NA***	***NA***

Pruritis (itching – no skin lesions) ***(See also Injection Site Reactions: Pruritis associated with injection)***	***Itching causing no or minimal interference with usual social & functional activities***	***Itching causing greater than minimal interference with usual social & functional activities***	***Itching causing inability to perform usual social & functional activities***	***NA***
CARDIOVASCULAR				
Cardiac arrhythmia (general) ***(By ECG or physical exam)***	***Asymptomatic AND No intervention indicated***	***Asymptomatic AND Non-urgent medical intervention indicated***	***Symptomatic, non-lifethreatening AND Nonurgent medical intervention indicated***	***Life-threatening arrhythmia OR Urgent intervention indicated***
Cardiac ischemia/ infarction	***NA***	***NA***	***Symptomatic ischemia (stable angina) OR Testing consistent with ischemia***	***Unstable angina OR Acute myocardial Infarction***

Basic Self-care Functions – Adult: Activities such as bathing, dressing, toileting, transfer/movement, continence, and feeding.

Basic Self-care Functions – Young Children: Activities that are age and culturally appropriate (e.g., feeding self with culturally appropriate eating implement).

Usual Social & Functional Activities – Adult: Adaptive tasks and desirable activities, such as going to work, shopping, cooking, use of transportation, pursuing a hobby, etc.

Usual Social & Functional Activities – Young Children: Activities that are age and culturally appropriate (e.g., social interactions, play activities, learning tasks, etc.).

CLINICAL				
**PARAMETER**	**GRADE 1 MILD**	**GRADE 2 MODERATE**	**GRADE 3 SEVERE**	**GRADE 4 POTENTIALLY LIFE-THREATENING**
Hemorrhage (significant acute blood loss)	***NA***	***Symptomatic AND No transfusion indicated***	***Symptomatic AND Transfusion of ≤ 2 units packed RBCs (for children ≤ 10 cc/kg) indicated***	***Life-threatening hypotension OR Transfusion of > 2 units packed RBCs (for children > 10 cc/kg) indicated***
Hypertension				
Adult > 17 years (with repeat testing at same visit)	***> 140 – 159 mmHg systolic OR > 90 – 99 mmHg diastolic***	***> 160 – 179 mmHg systolic OR > 100 – 109 mmHg diastolic***	***> 180 mmHg systolic OR > 110 mmHg diastolic***	***Life-threatening consequences (e.g., malignant hypertension) OR Hospitalization indicated (other than emergency room visit)***
Pediatric ≤ 17 years (with repeat testing at same visit)	***NA***	***91st – 94th percentile adjusted for age, height, and gender (systolic and/or diastolic)***	***≥ 95th percentile adjusted for age, height, and gender (systolic and/or diastolic)***	***Life-threatening consequences (e.g., malignant hypertension) OR Hospitalization indicated (other than emergency room visit)***
Hypotension	***NA***	***Symptomatic, corrected with oral fluid replacement***	***Symptomatic, IV fluids indicated***	***Shock requiring use of vasopressors or mechanical assistance to maintain blood pressure***

Pericardial effusion		***Asymptomatic, small effusion requiring no intervention***	***Asymptomatic, moderate or larger effusion requiring no intervention***	***Effusion with non-life threatening physiologic consequences OR Effusion with non-urgent intervention indicated***	***Life-threatening consequences (e.g., tamponade) OR Urgent intervention indicated***
Prolonged PR interval					
	Adult > 16 years	***PR interval 0.21 – 0.25 sec***	***PR interval > 0.25 sec***	***Type II 2nd degree AV block OR Ventricular pause > 3.0 sec***	***Complete AV block***
	Pediatric ≤16 Years	***1st degree AV block (PR > normal for age and rate)***	***Type I 2nd degree AV block***	***Type II 2nd degree AV block***	***Complete AV block***

Basic Self-care Functions – Adult: Activities such as bathing, dressing, toileting, transfer/movement, continence, and feeding.

Basic Self-care Functions – Young Children: Activities that are age and culturally appropriate (e.g., feeding self with culturally appropriate eating implement).

Usual Social & Functional Activities – Adult: Adaptive tasks and desirable activities, such as going to work, shopping, cooking, use of transportation, pursuing a hobby, etc.

Usual Social & Functional Activities – Young Children: Activities that are age and culturally appropriate (e.g., social interactions, play activities, learning tasks, etc.).

CLINICAL					
PARAMETER		GRADE 1 MILD	GRADE 2 MODERATE	GRADE 3 SEVERE	GRADE 4 POTENTIALLY LIFE-THREATENING
Prolonged QTc					
	Adult > 16 years	***Asymptomatic, QTc interval 0.45 – 0.47 sec OR Increase interval < 0.03 sec above baseline***	***Asymptomatic, QTc interval 0.48 – 0.49 sec OR Increase in interval 0.03 – 0.05 sec above baseline***	***Asymptomatic, QTc interval ≥ 0.50 sec OR Increase in interval ≥ 0.06 sec above baseline***	***Life-threatening consequences, e.g. Torsade de pointes or other associated serious ventricular dysrhythmia***
	Pediatric ≤ 16 years	***Asymptomatic, QTc interval 0.450 – 0.464 sec***	***Asymptomatic, QTc interval 0.465 – 0.479 sec***	***Asymptomatic, QTc interval ≥ 0.480 sec***	***Life-threatening consequences, e.g. Torsade de pointes or other associated serious ventricular dysrhythmia***
Thrombosis/embolism		***NA***	***Deep vein thrombosis AND No intervention indicated (e.g., anticoagulation, lysis filter, invasive procedure)***	***Deep vein thrombosis AND Intervention indicated (e.g., anticoagulation, lysis filter, invasive procedure)***	***Embolic event (e.g., pulmonary embolism, life-threatening thrombus)***
Vasovagal episode (associated with a procedure of any kind)		***Present without loss of consciousness***	***Present with transient loss of consciousness***	***NA***	***NA***
Ventricular dysfunction (congestive heart failure)		***NA***	***Asymptomatic diagnostic finding AND intervention indicated***	***New onset with symptoms OR Worsening symptomatic congestive heart failure***	***Life-threatening congestive heart failure***

GASTROINTESTINAL				
Anorexia	***Loss of appetite without decreased oral intake***	***Loss of appetite associated with decreased oral intake without significant weight loss***	***Loss of appetite associated with significant weight loss***	***Life-threatening consequences OR Aggressive intervention indicated [e.g., tube feeding or total parenteral nutrition (TPN)]***
Ascites	***Asymptomatic***	***Symptomatic AND Intervention indicated (e.g., diuretics or therapeutic paracentesis)***	***Symptomatic despite intervention***	***Life-threatening consequences***

Basic Self-care Functions – Adult: Activities such as bathing, dressing, toileting, transfer/movement, continence, and feeding.

Basic Self-care Functions – Young Children: Activities that are age and culturally appropriate (e.g., feeding self with culturally appropriate eating implement).

Usual Social & Functional Activities – Adult: Adaptive tasks and desirable activities, such as going to work, shopping, cooking, use of transportation, pursuing a hobby, etc.

Usual Social & Functional Activities – Young Children: Activities that are age and culturally appropriate (e.g., social interactions, play activities, learning tasks, etc.).

CLINICAL				
PARAMETER	GRADE 1<br>MILD	GRADE 2<br>MODERATE	GRADE 3<br>SEVERE	GRADE 4<br>POTENTIALLY LIFE-THREATENING
Cholecystitis	***NA***	***Symptomatic AND Medical intervention indicated***	***Radiologic, endoscopic, or operative intervention indicated***	***Life-threatening consequences (e.g., sepsis or perforation)***
Constipation	***NA***	***Persistent constipation requiring regular use of dietary modifications, laxatives, or enemas***	***Obstipation with manual evacuation indicated***	***Life-threatening consequences (e.g., obstruction)***
Diarrhoea				
Adult and Pediatric ≥ 1 year	***Transient or intermittent episodes of unformed stools OR Increase of ≤ 3 stools over baseline per 24-hour period***	***Persistent episodes of unformed to watery stools OR Increase of 4 – 6 stools over baseline per 24-hour period***	***Bloody diarrhoea OR Increase of ≥ 7 stools per 24-hour period OR IV fluid replacement indicated***	***Life-threatening consequences (e.g., hypotensive shock)***
Pediatric < 1 year	***Liquid stools (more unformed than usual) but usual number of stools***	***Liquid stools with increased number of stools OR Mild dehydration***	***Liquid stools with moderate dehydration***	***Liquid stools resulting in severe dehydration with aggressive rehydration indicated OR Hypotensive shock***
Dysphagia-Odynophagia	***Symptomatic but able to eat usual diet***	***Symptoms causing altered dietary intake without medical intervention indicated***	***Symptoms causing severely altered dietary intake with medical intervention indicated***	***Life-threatening reduction in oral intake***

Mucositis/stomatitis (clinical exam)<br>Indicate site (e.g., larynx, oral)<br>See Genitourinary for Vulvovaginitis<br>See also Dysphagia-Odynophagia and Proctitis	Erythema of the Mucosa	***Patchy pseudomembranes or ulcerations***	***Confluent pseudomembranes or ulcerations OR Mucosal bleeding with minor trauma***	***Tissue necrosis OR Diffuse spontaneous mucosal bleeding OR Life-threatening consequences (e.g., aspiration, choking)***
Nausea	Transient (< 24 hours) or intermittent nausea with no or minimal interference with oral intake	Persistent nausea resulting in decreased oral intake for 24 – 48 hours	***Persistent nausea resulting in minimal oral intake for > 48 hours OR Aggressive rehydration indicated (e.g., IV fluids)***	Life-threatening consequences (e.g., hypotensive shock)

Basic Self-care Functions – Adult: Activities such as bathing, dressing, toileting, transfer/movement, continence, and feeding.

Basic Self-care Functions – Young Children: Activities that are age and culturally appropriate (e.g., feeding self with culturally appropriate eating implement).

Usual Social & Functional Activities – Adult: Adaptive tasks and desirable activities, such as going to work, shopping, cooking, use of transportation, pursuing a hobby, etc.

Usual Social & Functional Activities – Young Children: Activities that are age and culturally appropriate (e.g., social interactions, play activities, learning tasks, etc.).

CLINICAL				
**PARAMETER**	**GRADE 1**<br><br>**MILD**	**GRADE 2**<br><br>**MODERATE**	**GRADE 3**<br><br>**SEVERE**	**GRADE 4**<br><br>**POTENTIALLY LIFE-THREATENING**
Pancreatitis	***NA***	***Symptomatic AND Hospitalization not indicated (other than emergency room visit)***	***Symptomatic AND Hospitalization indicated (other than emergency room visit)***	***Life-threatening consequences (e.g., circulatory failure, hemorrhage, sepsis)***
Proctitis (functional-symptomatic)<br>***Also see Mucositis/stomatitis for clinical exam***	***Rectal discomfort AND No intervention Indicated***	Symptoms causing greater than minimal interference with usual social & functional activities OR Medical intervention indicated	***Symptoms causing inability to perform usual social & functional activities OR Operative intervention indicated***	Life-threatening consequences (e.g., perforation)
Vomiting	Transient or intermittent vomiting with no or minimal interference with oral intake	Frequent episodes of vomiting with no or mild dehydration	Persistent vomiting resulting in orthostatic hypotension OR Aggressive rehydration indicated (e.g., IV fluids)	Life-threatening consequences (e.g., hypotensive shock)

NEUROLOGIC				
Alteration in personality-behavior or in mood<br>(e.g., agitation, anxiety, depression, mania, psychosis)	Alteration causing no or minimal interference with usual social & functional activities	Alteration causing greater than minimal interference with usual social & functional activities	Alteration causing inability to perform usual social & functional activities	Behavior potentially harmful to self or others<br>(e.g., suicidal and homicidal ideation or attempt, acute psychosis) OR Causing inability to perform basic self-care functions
Altered Mental Status<br>For Dementia, see Cognitive and behavioral/attentional disturbance (including dementia and attention deficit disorder)	Changes causing no or minimal interference with usual social & functional activities	Mild lethargy or somnolence causing greater than minimal interference with usual social & functional activities	Confusion, memory impairment, lethargy, or somnolence causing inability to perform usual social & functional activities	Delirium OR obtundation, OR coma
Ataxia	Asymptomatic ataxia detectable on exam OR Minimal ataxia causing no or minimal interference with usual social & functional activities	Symptomatic ataxia causing greater than minimal interference with usual social & functional activities	Symptomatic ataxia causing inability to perform usual social & functional activities	Disabling ataxia causing inability to perform basic self-care functions

Basic Self-care Functions – Adult: Activities such as bathing, dressing, toileting, transfer/movement, continence, and feeding.

Basic Self-care Functions – Young Children: Activities that are age and culturally appropriate (e.g., feeding self with culturally appropriate eating implement).

Usual Social & Functional Activities – Adult: Adaptive tasks and desirable activities, such as going to work, shopping, cooking, use of transportation, pursuing a hobby, etc.

Usual Social & Functional Activities – Young Children: Activities that are age and culturally appropriate (e.g., social interactions, play activities, learning tasks, etc.).

CLINICAL				
**PARAMETER**	**GRADE 1 MILD**	**GRADE 2 MODERATE**	**GRADE 3 SEVERE**	**GRADE 4 POTENTIALLY LIFE-THREATENING**
Cognitive and behavioural/attentional disturbance (including dementia and attention deficit disorder)	***Disability causing no or minimal interference with usual social & functional activities OR Specialized resources not indicated***	***Disability causing greater than minimal interference with usual social & functional activities OR Specialized resources on part-time basis indicated***	***Disability causing inability to perform usual social & functional activities OR Specialized resources on a full-time basis indicated***	***Disability causing inability to perform basic self-care functions OR Institutionalization Indicated***
CNS ischemia (acute)	NA	NA	Transient Ischemic Attack	Cerebral Vascular Accident (CVA, stroke) with neurological deficit
Developmental delay **– Pediatric ≤ 16 years**	Mild developmental delay, either motor or cognitive, as determined by comparison with a developmental screening tool appropriate for the setting	Moderate developmental delay, either motor or cognitive, as determined by comparison with a developmental screening tool appropriate for the setting	Severe developmental delay, either motor or cognitive, as determined by comparison with a developmental screening tool appropriate for the setting	Developmental regression, either motor or cognitive, as determined by comparison with a developmental screening tool appropriate for the setting

Headache	Symptoms causing no or minimal interference with usual social & functional activities	Symptoms causing greater than minimal interference with usual social & functional activities	Symptoms causing inability to perform usual social & functional activities	Symptoms causing inability to perform basic self-care functions OR Hospitalization indicated (other than emergency room visit) OR Headache with significant impairment of alertness or other neurologic function
Insomnia	NA	Difficulty sleeping causing greater than minimal interference with usual social & functional activities	Difficulty sleeping causing inability to perform usual social & functional activities	Disabling insomnia causing inability to perform basic self-care functions
Neuromuscular weakness (including myopathy & neuropathy)	Asymptomatic with decreased strength on exam OR Minimal muscle weakness causing no or minimal interference with usual social & functional activities	Muscle weakness causing greater than minimal interference with usual social & functional activities	Muscle weakness causing inability to perform usual social & functional activities	Disabling muscle weakness causing inability to perform basic self-care functions OR Respiratory muscle weakness impairing ventilation

Basic Self-care Functions – Adult: Activities such as bathing, dressing, toileting, transfer/movement, continence, and feeding.

Basic Self-care Functions – Young Children: Activities that are age and culturally appropriate (e.g., feeding self with culturally appropriate eating implement).

Usual Social & Functional Activities – Adult: Adaptive tasks and desirable activities, such as going to work, shopping, cooking, use of transportation, pursuing a hobby, etc.

Usual Social & Functional Activities – Young Children: Activities that are age and culturally appropriate (e.g., social interactions, play activities, learning tasks, etc.).

CLINICAL				
**PARAMETER**	**GRADE 1**<br>**MILD**	**GRADE 2**<br>**MODERATE**	**GRADE 3**<br>**SEVERE**	**GRADE 4**<br>**POTENTIALLY LIFE-THREATENING**
Neurosensory alteration (including paresthesia and painful neuropathy)	***Asymptomatic with sensory alteration on exam or minimal paresthesia causing no or minimal interference with usual social & functional activities***	***Sensory alteration or paresthesia causing greater than minimal interference with usual social & functional activities***	***Sensory alteration or paresthesia causing inability to perform usual social & functional activities***	***Disabling sensory alteration or paresthesia causing inability to perform basic self-care functions***
Seizure: (new onset) **– Adult ≥ 18 years**<br>See also Seizure: (known pre-existing seizure disorder)	NA	1 seizure	2 **–** 4 seizures	Seizures of any kind which are prolonged, repetitive (e.g., status epilepticus), or difficult to control (e.g., refractory epilepsy)
Seizure: (known preexisting seizure disorder)<br>**– Adult ≥ 18 years**<br>For worsening of existing epilepsy the grades should be based on an increase from previous level of control to any of these levels.	NA	Increased frequency of pre-existing seizures (non-repetitive) without change in seizure character OR infrequent breakthrough seizures while on stable medication in a previously controlled seizure disorder	Change in seizure character from baseline either in duration or quality (e.g., severity or focality)	Seizures of any kind which are prolonged, repetitive (e.g., status epilepticus), or difficult to control (e.g., refractory epilepsy)

Seizure **– Pediatric < 18 years**	Seizure, generalized onset with or without secondary generalization, lasting < 5 minutes with < 24 hours post ictal state	Seizure, generalized onset with or without secondary generalization, lasting 5 – 20 minutes with < 24 hours post ictal state	Seizure, generalized onset with or without secondary generalization, lasting > 20 minutes	Seizure, generalized onset with or without secondary generalization, requiring intubation and sedation
Syncope (not associated with a procedure)	NA	Present	NA	NA
Vertigo	Vertigo causing no or minimal interference with usual social & functional activities	Vertigo causing greater than minimal interference with usual social & functional activities	Vertigo causing inability to perform usual social & functional activities	Disabling vertigo causing inability to perform basic self-care functions

Basic Self-care Functions – Adult: Activities such as bathing, dressing, toileting, transfer/movement, continence, and feeding.

Basic Self-care Functions – Young Children: Activities that are age and culturally appropriate (e.g., feeding self with culturally appropriate eating implement).

Usual Social & Functional Activities – Adult: Adaptive tasks and desirable activities, such as going to work, shopping, cooking, use of transportation, pursuing a hobby, etc.

Usual Social & Functional Activities – Young Children: Activities that are age and culturally appropriate (e.g., social interactions, play activities, learning tasks, etc.).

CLINICAL					
PARAMETER		GRADE 1<br>MILD	GRADE 2<br>MODERATE	GRADE 3<br>SEVERE	GRADE 4<br>POTENTIALLY LIFE-THREATENING
**RESPIRATORY**					
Bronchospasm (acute)		***FEV1 or peak flow reduced to 70 – 80%***	***FEV1 or peak flow 50 – 69%***	***FEV1 or peak flow 25 – 49%***	***Cyanosis OR FEV1 or peak flow < 25% OR Intubation***
Dyspnea or respiratory distress					
	Adult ≥ 14 years	***Dyspnea on exertion with no or minimal interference with usual social & functional activities***	Dyspnea on exertion causing greater than minimal interference with usual social & functional activities	Dyspnea at rest causing inability to perform usual social & functional activities	Respiratory failure with ventilatory support indicated
	Pediatric < 14 Years	Wheezing OR minimal increase in respiratory rate for age	Nasal flaring OR Intercostal retractions OR Pulse oximetry 90 – 95%	Dyspnea at rest causing inability to perform usual social & functional activities OR Pulse oximetry < 90%	Respiratory failure with ventilatory support indicated
**MUSCULOSKELETAL**					
Arthralgia<br>See also Arthritis		Joint pain causing no or minimal interference with usual social & functional activities	Joint pain causing greater than minimal interference with usual social & functional activities	Joint pain causing inability to perform usual social & functional activities	Disabling joint pain causing inability to perform basic self-care functions

Arthritis<br>See also Arthralgia	Stiffness or joint swelling causing no or minimal interference with usual social & functional activities	Stiffness or joint swelling causing greater than minimal interference with usual social & functional activities	Stiffness or joint swelling causing inability to perform usual social & functional activities	Disabling joint stiffness or swelling causing inability to perform basic self-care functions
Bone Mineral Loss				
**Adult ≥ 21 years**	BMD t-score<br>-2.5 to -1.0	BMD t-score < -2.5	Pathological fracture (including loss of vertebral height)	Pathologic fracture causing life-threatening consequences
**Pediatric < 21 Years**	BMD z-score<br>-2.5 to -1.0	BMD z-score < -2.5	Pathological fracture (including loss of vertebral height)	Pathologic fracture causing life-threatening consequences

Basic Self-care Functions – Adult: Activities such as bathing, dressing, toileting, transfer/movement, continence, and feeding.

Basic Self-care Functions – Young Children: Activities that are age and culturally appropriate (e.g., feeding self with culturally appropriate eating implement).

Usual Social & Functional Activities – Adult: Adaptive tasks and desirable activities, such as going to work, shopping, cooking, use of transportation, pursuing a hobby, etc.

Usual Social & Functional Activities – Young Children: Activities that are age and culturally appropriate (e.g., social interactions, play activities, learning tasks, etc.).

CLINICAL				
PARAMETER	GRADE 1<br>MILD	GRADE 2<br>MODERATE	GRADE 3<br>SEVERE	GRADE 4<br>POTENTIALLY LIFE-THREATENING
Myalgia (non-injection site)	***Muscle pain causing no or minimal interference with usual social & functional activities***	***Muscle pain causing greater than minimal interference with usual social & functional activities***	***Muscle pain causing inability to perform usual social & functional activities***	***Disabling muscle pain causing inability to perform basic self-care functions***
Osteonecrosis	NA	Asymptomatic with radiographic findings AND No operative intervention indicated	Symptomatic bone pain with radiographic findings OR Operative intervention indicated	Disabling bone pain with radiographic findings causing inability to perform basic self-care functions
**GENITOURINARY**				
Cervicitis (symptoms)<br>(For use in studies evaluating topical study agents)<br>*For other cervicitis see Infection: Infection (any other than HIV infection)*	Symptoms causing no or minimal interference with usual social & functional activities	Symptoms causing greater than minimal interference with usual social & functional activities	Symptoms causing inability to perform usual social & functional activities	Symptoms causing inability to perform basic self-care functions

Cervicitis (<u>clinical exam</u>) (For use in studies evaluating topical study agents) For other cervicitis see Infection: Infection (any other than HIV infection)	Minimal cervical abnormalities on examination (erythema, mucopurulent discharge, or friability) OR Epithelial disruption < 25% of total surface	Moderate cervical abnormalities on examination (erythema, mucopurulent discharge, or friability) OR Epithelial disruption of 25 – 49% total surface	Severe cervical abnormalities on examination (erythema, mucopurulent discharge, or friability) OR Epithelial disruption 50 – 75% total surface	Epithelial disruption > 75% total surface
Inter-menstrual bleeding (IMB)	Spotting observed by participant OR Minimal blood observed during clinical or colposcopic examination	Inter-menstrual bleeding not greater in duration or amount than usual menstrual cycle	Inter-menstrual bleeding greater in duration or amount than usual menstrual cycle	Hemorrhage with lifethreatening hypotension OR Operative intervention indicated
Urinary tract obstruction (e.g., stone)	NA	Signs or symptoms of urinary tract obstruction without hydronephrosis or renal dysfunction	Signs or symptoms of urinary tract obstruction with hydronephrosis or renal dysfunction	Obstruction causing lifethreatening Consequences

Basic Self-care Functions – Adult: Activities such as bathing, dressing, toileting, transfer/movement, continence, and feeding.

Basic Self-care Functions – Young Children: Activities that are age and culturally appropriate (e.g., feeding self with culturally appropriate eating implement).

Usual Social & Functional Activities – Adult: Adaptive tasks and desirable activities, such as going to work, shopping, cooking, use of transportation, pursuing a hobby, etc.

Usual Social & Functional Activities – Young Children: Activities that are age and culturally appropriate (e.g., social interactions, play activities, learning tasks, etc.).

CLINICAL				
**PARAMETER**	**GRADE 1 MILD**	**GRADE 2 MODERATE**	**GRADE 3 SEVERE**	**GRADE 4 POTENTIALLY LIFE-THREATENING**
Vulvovaginitis (symptoms) (Use in studies evaluating topical study agents) *For other vulvovaginitis see Infection: Infection (any other than HIV infection)*	***Symptoms causing no or minimal interference with usual social & functional activities***	***Symptoms causing greater than minimal interference with usual social & functional activities***	***Symptoms causing inability to perform usual social & functional activities***	***Symptoms causing inability to perform basic self-care functions***
Vulvovaginitis (clinical exam) (Use in studies evaluating topical study agents) For other vulvovaginitis see Infection: Infection (any other than HIV infection)	Minimal vaginal abnormalities on examination OR Epithelial disruption < 25% of total surface	Moderate vaginal abnormalities on examination OR Epithelial disruption of 25 - 49% total surface	Severe vaginal abnormalities on examination OR Epithelial disruption 50 - 75% total surface	Vaginal perforation OR Epithelial disruption > 75% total surface
**OCULAR/VISUAL**				
Uveitis	Asymptomatic but detectable on exam	Symptomatic anterior uveitis OR Medical intervention indicated	Posterior or pan-uveitis OR Operative intervention indicated	Disabling visual loss in affected eye(s)

Visual changes (from baseline)	Visual changes causing no or minimal interference with usual social & functional activities	Visual changes causing greater than minimal interference with usual social & functional activities	Visual changes causing inability to perform usual social & functional activities	Disabling visual loss in affected eye(s)
**ENDOCRINE/METABOLIC**				
Abnormal fat accumulation (e.g., back of neck, breasts, abdomen)	Detectable by study participant (or by caregiver for young children and disabled adults)	Detectable on physical exam by health care provider	Disfiguring OR Obvious changes on casual visual inspection	NA

Basic Self-care Functions – Adult: Activities such as bathing, dressing, toileting, transfer/movement, continence, and feeding.

Basic Self-care Functions – Young Children: Activities that are age and culturally appropriate (e.g., feeding self with culturally appropriate eating implement).

Usual Social & Functional Activities – Adult: Adaptive tasks and desirable activities, such as going to work, shopping, cooking, use of transportation, pursuing a hobby, etc.

Usual Social & Functional Activities – Young Children: Activities that are age and culturally appropriate (e.g., social interactions, play activities, learning tasks, etc.).

CLINICAL				
**PARAMETER**	**GRADE 1 MILD**	**GRADE 2 MODERATE**	**GRADE 3 SEVERE**	**GRADE 4 POTENTIALLY LIFE-THREATENING**
Diabetes mellitus	***NA***	***New onset without need to initiate medication OR Modification of current medications to regain glucose control***	***New onset with initiation of medication indicated OR Diabetes uncontrolled despite treatment modification***	***Life-threatening consequences (e.g., ketoacidosis, hyperosmolar nonketotic coma)***
Gynecomastia	***Detectable by study participant or caregiver (for young children and disabled adults)***	Detectable on physical exam by health care provider	Disfiguring OR Obvious on casual visual inspection	NA
Hyperthyroidism	Asymptomatic	Symptomatic causing greater than minimal interference with usual social & functional activities OR Thyroid suppression therapy indicated	Symptoms causing inability to perform usual social & functional activities OR Uncontrolled despite treatment modification	Life-threatening consequences (e.g., thyroid storm)
Hypothyroidism	Asymptomatic	Symptomatic causing greater than minimal interference with usual social & functional activities OR Thyroid replacement therapy indicated	Symptoms causing inability to perform usual social & functional activities OR Uncontrolled despite treatment modification	Life-threatening consequences (e.g., myxedema coma)

Lipoatrophy (e.g., fat loss from the face, extremities, buttocks)	Detectable by study participant (or by caregiver for young children and disabled adults)	Detectable on physical exam by health care provider	Disfiguring OR Obvious on casual visual inspection	NA

Basic Self-care Functions – Adult: Activities such as bathing, dressing, toileting, transfer/movement, continence, and feeding.

Basic Self-care Functions – Young Children: Activities that are age and culturally appropriate (e.g., feeding self with culturally appropriate eating implement).

Usual Social & Functional Activities – Adult: Adaptive tasks and desirable activities, such as going to work, shopping, cooking, use of transportation, pursuing a hobby, etc.

Usual Social & Functional Activities – Young Children: Activities that are age and culturally appropriate (e.g., social interactions, play activities, learning tasks, etc.).

LABORATORY					
PARAMETER		GRADE 1<br>MILD	GRADE 2<br>MODERATE	GRADE 3<br>SEVERE	GRADE 4<br>POTENTIALLY LIFE-THREATENING
**HEMATOLOGY** ***Standard International Units are listed in italics***					
Absolute CD4+ count<br>**– Adult and Pediatric > 13 years**<br>(HIV NEGATIVE ONLY)		300 – 400/mm³<br>*300 – 400/µL*	200 – 299/mm³<br>*200 – 299/µL*	100 – 199/mm³<br>*100 – 199/µL*	< 100/mm³<br>*< 100/µL*
Absolute lymphocyte count<br>**– Adult and Pediatric > 13 years**<br>(HIV NEGATIVE ONLY)		600 – 650/mm³<br>*0.600 x 10^9 – 0.650 x 10^9/L*	500 – 599/mm³<br>*0.500 x 10^9 – 0.599 x 10^9/L*	350 – 499/mm³<br>*0.350 x 10^9 – 0.499 x 10^9/L*	< 350/mm³<br>*< 0.350 x 10^9/L*
Absolute neutrophil count (ANC)					
	**Adult and Pediatric, > 7 days**	1,000 – 1,300/mm³<br>*1.000 x 10^9 – 1.300 x 10^9/L*	750 – 999/mm³<br>*0.750 x 10^9 – 0.999 x 10^9/L*	500 – 749/mm³<br>*0.500 x 10^9 – 0.749 x 10^9/L*	< 500/mm³<br>*< 0.500 x 10^9/L*
	**Infant•†, 2 – ≤ 7 days**	1,250 – 1,500/mm³<br>*1.250 x 10^9 – 1.500 x 10^9/L*	1,000 – 1,249/mm³<br>*1.000 x 10^9 – 1.249 x 10^9/L*	750 – 999/mm³<br>*0.750 x 10^9 – 0.999 x 10^9/L*	< 750/mm³<br>*< 0.750 x 10^9/L*
	**Infant•†, 1 day**	4,000 – 5,000/mm³<br>*4.000 x 10^9 – 5.000 x 10^9/L*	3,000 – 3,999/mm³<br>*3.000 x 10^9 – 3.999 x10^9/L*	1,500 – 2,999/mm³<br>*1.500 x 10^9 – 2.999 x 10^9/L*	< 1,500/mm³<br>*< 1.500 x 10^9/L*

Fibrinogen, decreased	100 – 200 mg/dL<br>*1.00 – 2.00 g/L*<br>OR<br>0.75 – 0.99 x LLN	75 – 99 mg/dL<br>*0.75 – 0.99 g/L*<br>OR<br>0.50 – 0.74 x LLN	50 – 74 mg/dL<br>*0.50 – 0.74 g/L*<br>OR<br>0.25 – 0.49 x LLN	< 50 mg/dL<br>*< 0.50 g/L*<br>OR<br>< 0.25 x LLN<br>OR Associated with gross Bleeding
Hemoglobin (Hgb)				
**Adult and Pediatric ≥ 57 days** (HIV POSITIVE ONLY)	8.5 – 10.0 g/dL<br>*1.32 – 1.55 mmol/L*	7.5 – 8.4 g/dL<br>*1.16 – 1.31 mmol/L*	6.50 – 7.4 g/dL<br>*1.01 – 1.15 mmol/L*	< 6.5 g/dL<br>*< 1.01 mmol/L*
**Adult and Pediatric ≥ 57 days** (HIV NEGATIVE ONLY)	10.0 – 10.9 g/dL<br>*1.55 – 1.69 mmol/L*<br>OR<br>Any decrease<br>2.5 – 3.4 g/dL<br>*0.39 – 0.53 mmol/L*	9.0 – 9.9 g/dL<br>*1.40 – 1.54 mmol/L*<br>OR<br>Any decrease<br>3.5 – 4.4 g/dL<br>*0.54 – 0.68 mmol/L*	7.0 – 8.9 g/dL<br>*1.09 – 1.39 mmol/L*<br>OR<br>Any decrease<br>≥ 4.5 g/dL<br>*≥ 0.69 mmol/L*	< 7.0 g/dL<br>*< 1.09 mmol/L*
**Infant[•,†], 36 – 56 days** (HIV POSITIVE OR NEGATIVE)	8.5 – 9.4 g/dL<br>*1.32 – 1.46 mmol/L*	7.0 – 8.4 g/dL<br>*1.09 – 1.31 mmol/L*	6.0 – 6.9 g/dL<br>*0.93 – 1.08 mmol/L*	< 6.00 g/dL<br>*< 0.93 mmol/L*

[•] Values are for term infants.

[†] Use age and sex appropriate values (e.g., bilirubin), including preterm infants.

LABORATORY				
**PARAMETER**	**GRADE 1 MILD**	**GRADE 2 MODERATE**	**GRADE 3 SEVERE**	**GRADE 4 POTENTIALLY LIFE-THREATENING**
**Infant•†, 22 – 35 days** *(HIV POSITIVE OR NEGATIVE)*	9.5 – 10.5 g/dL<br>1.47 – 1.63 mmol/L	8.0 – 9.4 g/dL<br>1.24 – 1.46 mmol/L	7.0 – 7.9 g/dL<br>1.09 – 1.23 mmol/L	< 7.00 g/dL<br>< 1.09 mmol/L
**Infant•†, 1 – 21 days** (HIV POSITIVE OR NEGATIVE)	12.0 – 13.0 g/dL<br>*1.86 – 2.02 mmol/L*	10.0 – 11.9 g/dL<br>*1.55 – 1.85 mmol/L*	9.0 – 9.9 g/dL<br>*1.40 – 1.54 mmol/L*	< 9.0 g/dL<br>*< 1.40 mmol/L*
International Normalized Ratio of prothrombin time (INR)	1.1 – 1.5 x ULN	1.6 – 2.0 x ULN	2.1 – 3.0 x ULN	> 3.0 x ULN
Methemoglobin	5.0 – 10.0%	10.1 – 15.0%	15.1 – 20.0%	> 20.0%
Prothrombin Time (PT)	1.1 – 1.25 x ULN	1.26 – 1.50 x ULN	1.51 – 3.00 x ULN	> 3.00 x ULN
Partial Thromboplastin Time (PTT)	1.1 – 1.66 x ULN	1.67 – 2.33 x ULN	2.34 – 3.00 x ULN	> 3.00 x ULN
Platelets, decreased	100,000 – 124,999/mm³<br>*100.000 x 10⁹ – 124.999 x 10⁹/L*	50,000 – 99,999/mm³<br>*50.000 x 10⁹ – 99.999 x 10⁹/L*	25,000 – 49,999/mm³<br>*25.000 x 10⁹ – 49.999 x 10⁹/L*	< 25,000/mm³<br>*< 25.000 x 10⁹/L*
WBC, decreased	2,000 – 2,500/mm³<br>*2.000 x 10⁹ – 2.500 x 10⁹/L*	1,500 – 1,999/mm³<br>*1.500 x 10⁹ – 1.999 x 10⁹/L*	1,000 – 1,499/mm³<br>*1.000 x 10⁹ – 1.499 x 10⁹/L*	< 1,000/mm³<br>*< 1.000 x 10⁹/L*
**CHEMISTRIES** ***Standard International Units are listed in italics***				
Acidosis	NA	pH < normal, but ≥ 7.3	pH < 7.3 without lifethreatening Consequences	pH < 7.3 with lifethreatening consequences

Albumin, serum, low	3.0 g/dL – < LLN<br>*30 g/L – < LLN*	2.0 – 2.9 g/dL<br>*20 – 29 g/L*	< 2.0 g/dL<br>*< 20 g/L*	NA
Alkaline Phosphatase	1.25 – 2.5 x ULN[†]	2.6 – 5.0 x ULN[†]	5.1 – 10.0 x ULN[†]	> 10.0 x ULN[†]
Alkalosis	NA	pH > normal, but ≤ 7.5	pH > 7.5 without lifethreatening Consequences	pH > 7.5 with lifethreatening consequences
ALT (SGPT)	1.25 – 2.5 x ULN	2.6 – 5.0 x ULN	5.1 – 10.0 x ULN	> 10.0 x ULN
AST (SGOT)	1.25 – 2.5 x ULN	2.6 – 5.0 x ULN	5.1 – 10.0 x ULN	> 10.0 x ULN
Bicarbonate, serum, low	16.0 mEq/L – < LLN<br>*16.0 mmol/L – < LLN*	11.0 – 15.9 mEq/L<br>*11.0 – 15.9 mmol/L*	8.0 – 10.9 mEq/L<br>*8.0 – 10.9 mmol/L*	< 8.0 mEq/L<br>*< 8.0 mmol/L*
Bilirubin (Total)				
**Adult and Pediatric >14 days**	1.1 – 1.5 x ULN	1.6 – 2.5 x ULN	2.6 – 5.0 x ULN	> 5.0 x ULN

• Values are for term infants.

[†] Use age and sex appropriate values (e.g., bilirubin), including preterm infants.

LABORATORY				
**PARAMETER**	**GRADE 1 MILD**	**GRADE 2 MODERATE**	**GRADE 3 SEVERE**	**GRADE 4 POTENTIALLY LIFE-THREATENING**
Infant•†, ≤14 days *(non-hemolytic)*	*NA*	20.0 – 25.0 mg/dL<br>342 – 428 µmol/L	25.1 – 30.0 mg/dL<br>429 – 513 µmol/L	> 30.0 mg/dL<br>> 513.0 µmol/L
**Infant•†, ≤ 14 days** (hemolytic)	*NA*	NA	20.0 – 25.0 mg/dL<br>*342 – 428 µmol/L*	> 25.0 mg/dL<br>*> 428 µmol/L*
Calcium, serum, high (corrected for albumin)				
**Adult and Pediatric ≥ 7 days**	10.6 – 11.5 mg/dL<br>2.65 – 2.88 mmol/L	11.6 – 12.5 mg/dL<br>*2.89 – 3.13 mmol/L*	12.6 – 13.5 mg/dL<br>*3.14 – 3.38 mmol/L*	> 13.5 mg/dL<br>*> 3.38 mmol/L*
**Infant•†, < 7 days**	11.5 – 12.4 mg/dL<br>*2.88 – 3.10 mmol/L*	12.5 – 12.9 mg/dL<br>*3.11 – 3.23 mmol/L*	13.0 – 13.5 mg/dL<br>*3.245 – 3.38 mmol/L*	> 13.5 mg/dL<br>*> 3.38 mmol/L*
Calcium, serum, low (corrected for albumin)				
**Adult and Pediatric ≥ 7 days**	7.8 – 8.4 mg/dL<br>*1.95 – 2.10 mmol/L*	7.0 – 7.7 mg/dL<br>*1.75 – 1.94 mmol/L*	6.1 – 6.9 mg/dL<br>*1.53 – 1.74 mmol/L*	< 6.1 mg/dL<br>*< 1.53 mmol/L*
**Infant•†, < 7 days**	6.5 – 7.5 mg/dL<br>*1.63 – 1.88 mmol/L*	6.0 – 6.4 mg/dL<br>*1.50 – 1.62 mmol/L*	5.50 – 5.90 mg/dL<br>*1.38 – 1.51 mmol/L*	< 5.50 mg/dL<br>*< 1.38 mmol/L*
Cardiac troponin I (cTnI)	NA	NA	NA	Levels consistent with myocardial infarction or unstable angina as defined by the manufacturer

Cardiac troponin T (cTnT)		NA	NA	NA	≥ 0.20 ng/mL OR Levels consistent with myocardial infarction or unstable angina as defined by the manufacturer
Cholesterol (fasting)					
	**Adult ≥ 18 years**	200 – 239 mg/dL<br>*5.18 – 6.19 mmol/L*	240 – 300 mg/dL<br>*6.20 – 7.77 mmol/L*	> 300 mg/dL<br>*> 7.77 mmol/L*	NA
	**Pediatric < 18 years**	170 – 199 mg/dL<br>*4.40 – 5.15 mmol/L*	200 – 300 mg/dL<br>*5.16 – 7.77 mmol/L*	> 300 mg/dL<br>*> 7.77 mmol/L*	NA
Creatine Kinase		3.0 – 5.9 x ULN†	6.0 – 9.9 x ULN†	10.0 – 19.9 x ULN†	≥ 20.0 x ULN†
Creatinine		1.1 – 1.3 x ULN†	1.4 – 1.8 x ULN†	1.9 – 3.4 x ULN†	≥ 3.5 x ULN†
Glucose, serum, high					
	Nonfasting	116 – 160 mg/dL<br>*6.44 – 8.88 mmol/L*	161 – 250 mg/dL<br>*8.89 – 13.88 mmol/L*	251 – 500 mg/dL<br>*13.89 – 27.75 mmol/L*	> 500 mg/dL<br>*> 27.75 mmol/L*
	Fasting	110 – 125 mg/dL<br>*6.11 – 6.94 mmol/L*	126 – 250 mg/dL<br>*6.95 – 13.88 mmol/L*	251 – 500 mg/dL<br>*13.89 – 27.75 mmol/L*	> 500 mg/dL<br>*> 27.75 mmol/L*

• Values are for term infants.

† Use age and sex appropriate values (e.g., bilirubin), including preterm infants.

LABORATORY				
**PARAMETER**	**GRADE 1 MILD**	**GRADE 2 MODERATE**	**GRADE 3 SEVERE**	**GRADE 4 POTENTIALLY LIFE-THREATENING**
Glucose, serum, low				
Adult and Pediatric ≥1 month	55 – 64 mg/dL 3.05 – 3.55 mmol/L	40 – 54 mg/dL 2.22 – 3.06 mmol/L	30 – 39 mg/dL 1.67 – 2.23 mmol/L	< 30 mg/dL < 1.67 mmol/L
**Infant•†, < 1 month**	50 – 54 mg/dL *2.78 – 3.00 mmol/L*	40 – 49 mg/dL *2.22 – 2.77 mmol/L*	30 – 39 mg/dL *1.67 – 2.21 mmol/L*	< 30 mg/dL *< 1.67 mmol/L*
Lactate	< 2.0 x ULN without acidosis	≥ 2.0 x ULN without acidosis	Increased lactate with pH < 7.3 without lifethreatening Consequences	Increased lactate with pH < 7.3 with lifethreatening consequences
LDL cholesterol (fasting)				
**Adult ≥ 18 years**	130 – 159 mg/dL *3.37 – 4.12 mmol/L*	160 – 190 mg/dL *4.13 – 4.90 mmol/L*	≥ 190 mg/dL *≥4.91 mmol/L*	NA
**Pediatric > 2 - < 18 Years**	110 – 129 mg/dL *2.85 – 3.34 mmol/L*	130 – 189 mg/dL *3.35 – 4.90 mmol/L*	≥ 190 mg/dL *≥4.91 mmol/L*	NA
Lipase	1.1 – 1.5 x ULN	1.6 – 3.0 x ULN	3.1 – 5.0 x ULN	> 5.0 x ULN
Magnesium, serum, low	1.2 – 1.4 mEq/L *0.60 – 0.70 mmol/L*	0.9 – 1.1 mEq/L *0.45 – 0.59 mmol/L*	0.6 – 0.8 mEq/L *0.30 – 0.44 mmol/L*	< 0.60 mEq/L *< 0.30 mmol/L*
Pancreatic amylase	1.1 – 1.5 x ULN	1.6 – 2.0 x ULN	2.1 – 5.0 x ULN	> 5.0 x ULN
Phosphate, serum, low				
**Adult and Pediatric > 14 years**	2.5 mg/dL – < LLN *0.81 mmol/L – < LLN*	2.0 – 2.4 mg/dL *0.65 – 0.80 mmol/L*	1.0 – 1.9 mg/dL *0.32 – 0.64 mmol/L*	< 1.00 mg/dL *< 0.32 mmol/L*
**Pediatric 1 year – 14 Years**	3.0 – 3.5 mg/dL *0.97 – 1.13 mmol/L*	2.5 – 2.9 mg/dL *0.81 – 0.96 mmol/L*	1.5 – 2.4 mg/dL *0.48 – 0.80 mmol/L*	< 1.50 mg/dL *< 0.48 mmol/L*
**Pediatric < 1 year**	3.5 – 4.5 mg/dL *1.13 – 1.45 mmol/L*	2.5 – 3.4 mg/dL *0.81 – 1.12 mmol/L*	1.5 – 2.4 mg/dL *0.48 – 0.80 mmol/L*	< 1.50 mg/dL *< 0.48 mmol/L*

Potassium, serum, high	5.6 – 6.0 mEq/L<br>*5.6 – 6.0 mmol/L*	6.1 – 6.5 mEq/L<br>*6.1 – 6.5 mmol/L*	6.6 – 7.0 mEq/L<br>*6.6 – 7.0 mmol/L*	> 7.0 mEq/L<br>*> 7.0 mmol/L*
Potassium, serum, low	3.0 – 3.4 mEq/L<br>*3.0 – 3.4 mmol/L*	2.5 – 2.9 mEq/L<br>*2.5 – 2.9 mmol/L*	2.0 – 2.4 mEq/L<br>*2.0 – 2.4 mmol/L*	< 2.0 mEq/L<br>*< 2.0 mmol/L*
Sodium, serum, high	146 – 150 mEq/L<br>*146 – 150 mmol/L*	151 – 154 mEq/L<br>*151 – 154 mmol/L*	155 – 159 mEq/L<br>*155 – 159 mmol/L*	≥ 160 mEq/L<br>*≥ 160 mmol/L*
Sodium, serum, low	130 – 135 mEq/L<br>*130 – 135 mmol/L*	125 – 129 mEq/L<br>*125 – 129 mmol/L*	121 – 124 mEq/L<br>*121 – 124 mmol/L*	≤ 120 mEq/L<br>*≤ 120 mmol/L*
Triglycerides (fasting)	NA	500 – 750 mg/dL<br>*5.65 – 8.48 mmol/L*	751 – 1,200 mg/dL<br>*8.49 – 13.56 mmol/L*	> 1,200 mg/dL<br>*> 13.56 mmol/L*
Uric acid	7.5 – 10.0 mg/dL<br>*0.45 – 0.59 mmol/L*	10.1 – 12.0 mg/dL<br>*0.60 – 0.71 mmol/L*	12.1 – 15.0 mg/dL<br>*0.72 – 0.89 mmol/L*	> 15.0 mg/dL<br>*> 0.89 mmol/L*

• Values are for term infants.

† Use age and sex appropriate values (e.g., bilirubin), including preterm infants.

LABORATORY					
PARAMETER		GRADE 1<br>MILD	GRADE 2<br>MODERATE	GRADE 3<br>SEVERE	GRADE 4<br>POTENTIALLY LIFE-THREATENING
**URINALYSIS** ***Standard International Units are listed in italics***					
Haematuria (microscopic)		6 – 10 RBC/HPF	> 10 RBC/HPF	Gross, with or without<br>clots OR with RBC Casts	Transfusion indicated
Proteinuria, random collection		1 +	2 – 3 +	4 +	NA
Proteinuria, 24 hour collection					
	Adult and Pediatric ≥ 10 years	200 – 999 mg/24 h<br>*0.200 – 0.999 g/d*	1,000 – 1,999 mg/24 h<br>*1.000 – 1.999 g/d*	2,000 – 3,500 mg/24 h<br>*2.000 – 3.500 g/d*	> 3,500 mg/24 h<br>*> 3.500 g/d*
	Pediatric > 3 mo - < 10 years	201 – 499 mg/m2/24 h<br>*0.201 – 0.499 g/d*	500 – 799 mg/m2/24 h<br>*0.500 – 0.799 g/d*	800 – 1,000 mg/m2/24 h<br>*0.800 – 1.000 g/d*	> 1,000 mg/ m2/24 h<br>*> 1.000 sg/d*

• Values are for term infants.

† Use age and sex appropriate values (e.g., bilirubin), including preterm infants.

16.3 Appendix 3 Safety Reporting Flow Chart

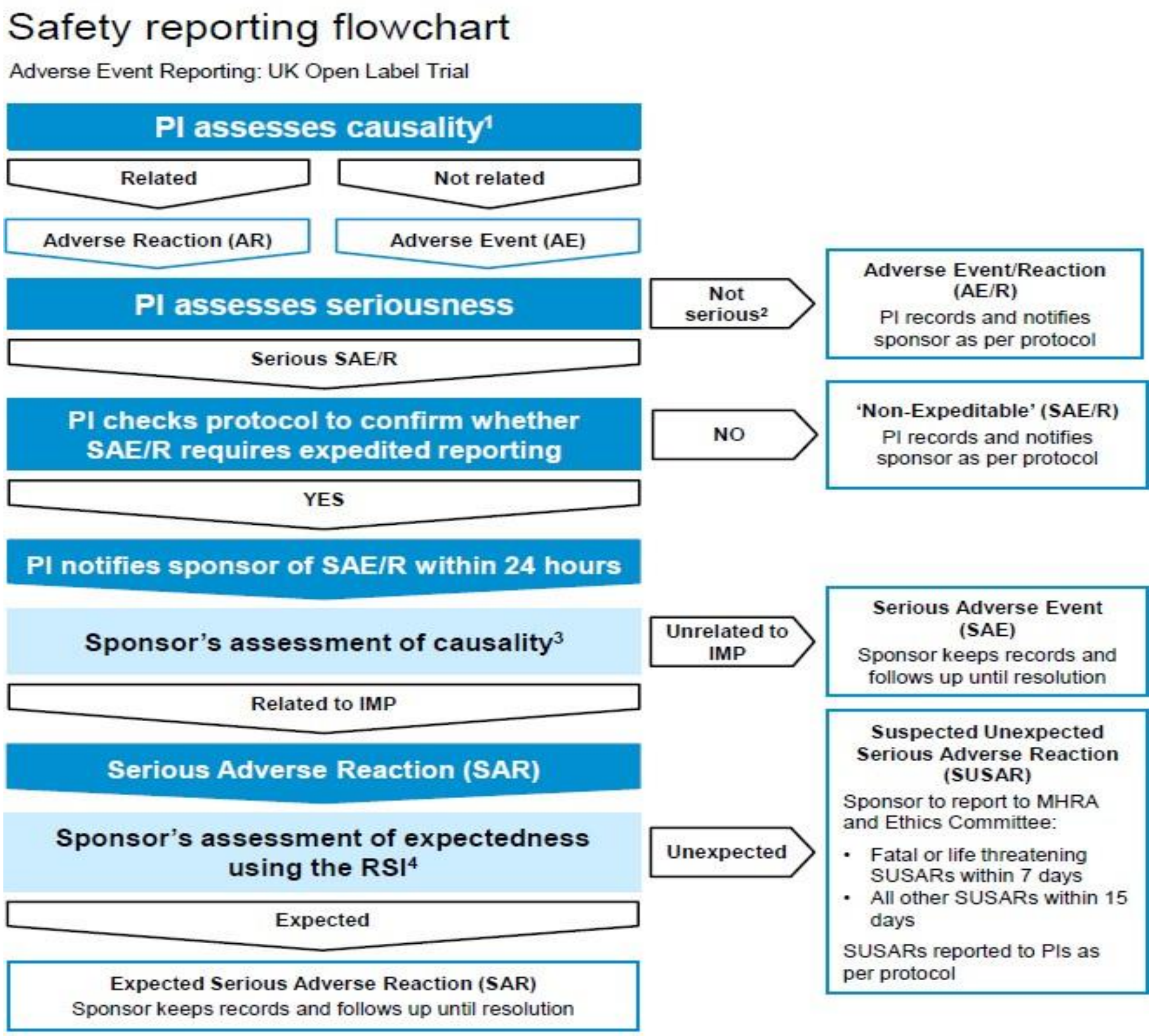

Adverse Event (AE):
Any untoward medical occurrence in a clinical trial subject administered a medicinal product and which does not necessarily have a causal relationship with this treatment.

Adverse Reaction (AR):
Any untoward and unintended response to an IMP which is related (a reasonable causal relationship) to any dose administered.

Serious Adverse Event/Reaction (SAE/R):
- Results in death,
- is life-threatening,
- requires hospitalisation or prolongation of existing hospitalisation,
- results in persistent or significant disability or incapacity,
- is a congenital anomaly or birth defect,
- any other safety issues considered medically important.

PI should **actively seek** follow-up information on reported SAE/Rs.

Footnotes
[1] PI or delegate.
[2] Notable or safety critical events must be reported as per protocol.
[3] Sponsor cannot downgrade the PI's causality assessment, but can upgrade it.
[4] Reference Safety Information (RSI) in IB or SmPC.

16.4 Appendix 4 Amendment History

Amendment No.	Protocol version no.	Date issued	Author(s) of changes	Details of changes made
01	3	20 Feb 2018	Dan Bradshaw	1. Addition of contraindicated concomitant medication of St John's Wort as an exclusion criteria<br>2. Addition of diet / exercise history to the study timeline at baseline, week 48 and 96 and early termination visit. Diet and exercise is standard of care for patients with non-alcoholic fatty liver disease and is a set of questions asked during consultation.<br>3. Alcohol assessment – completed by the patient at screening.<br>4. Addition of phosphate, CK, coeliac serology and thyroid function to the existing blood tests (no additional volume required)<br>5. We have removed the specific names of individuals form the Trial Management groups and Data management committee listed in protocol to avoid future amendments when staff change.<br>6. Informed consent visit removed to avoid additional visits and fasting glucose and lipids now moved to the baseline visit. This has been done on the recommendation of the clinical team who think this will assist in recruitment.<br>7. Clarification added to the exclusion criteria – point 6

				regarding definition of liver disease in accordance with European guidelines.<br>8. Volume of blood draw decreased from 198ml to 192ml
02	4.0	19th Nov 2018	Dan Bradshaw	1. Extension of window between Screen and Baseline from 28 to 42 days<br>2. Urine dip and pregnancy test (for WOCBP) included in the trial flow chart at termination visit<br>3. Clarification of definition of Trial Management Group, Trial Steering Committee and Data Safety and Monitoring Board<br>4. Clarification of AE reporting<br>5. Clarification of definition of creatinine clearance and preferred tool for calculating this<br>6. Clarification of the procedures for processing bloods for the ELF score and tropism test<br>7. Change in exclusion criterion (7) 'ALT or AST' raised from '>3x the ULN' to '>5x the ULN' (where ULN is defined as 41 IU/L)<br>8. Change of exclusion criterion (5), Addition of an alternative test for confirming HCV status: HCV antigen test and shortening the window of HCV clearance from 12 months to 6 months: Current HCV or HBV (HBcAb-positive, HBsAg-negative is permitted; anti-HCV Ab positive with HCV RNA or HCV antigen negative for ≥ 6 months following treatment or spontaneous clearance is permitted)<br>9. Clarification of the window between the visit and fibroscan date and the visit and the CT scan:

				‘to be within 7 days of the study visit’.<br>10. Clarification of AIDS related conditions: appendix 5 to the protocol, section 16.5

List details of all protocol amendments here whenever a new version of the protocol is produced.

Protocol amendments must be submitted to the Sponsor for approval prior to submission to the REC committee or MHRA.

16.5 Appendix 5

**CDC Classification of Clinical Categories for HIV-associated conditions (MMWR, 63: 1-10, 2014; MMWR, 41: 1-19, 1992)**		
**A**	**B**	**C (AIDS defining)**
Asymptomatic HIV infection	Bacillary angiomatosis	Bacterial infections, multiple or recurrent*
Persistent generalized lymphadenopathy	Candidiasis, oropharyngeal (thrush)	Candidiasis of bronchi, trachea, or lungs
Acute (primary) HIV infection with accompanying illness	Candidiasis, vulvovaginal; persistent, frequent, or poorly responsive to therapy	Candidiasis of esophagus
	Cervical dysplasia (moderate or severe)/cervical carcinoma in situ	Cervical cancer, invasive
	Constitutional symptoms, such as fever (38.5 C) or diarrhea lasting greater than 1 month	Coccidioidomycosis, disseminated or extrapulmonary
	Hairy leukoplakia, oral	Cryptococcosis, extrapulmonary
	Herpes zoster (shingles), involving at least two distinct episodes or more than one dermatome	Cryptosporidiosis, chronic intestinal (>1 month's duration)

	Idiopathic thrombocytopenic purpura	Cytomegalovirus disease (other than liver, spleen, or nodes), onset at age >1 month
	Listeriosis	Cytomegalovirus retinitis (with loss of vision)
	Pelvic inflammatory disease, particularly if complicated by tubo-ovarian abscess	Encephalopathy attributed to HIV
	Peripheral neuropathy	Herpes simplex: chronic ulcers (>1 month's duration) or bronchitis, pneumonitis, or esophagitis (onset at age >1 month)
		Histoplasmosis, disseminated or extrapulmonary
		Isosporiasis, chronic intestinal (>1 month's duration)
		Kaposi sarcoma
		Lymphoma, Burkitt (or equivalent term)
		Lymphoma, immunoblastic (or equivalent term)
		Lymphoma, primary, of brain
		Mycobacterium avium complex or *Mycobacterium kansasii*, disseminated or extrapulmonary
		Mycobacterium tuberculosis of any site, pulmonary†, disseminated, or extrapulmonary
		Mycobacterium, other species or unidentified species, disseminated or extrapulmonary
		Pneumocystis jirovecii (previously known as "*Pneumocystis carinii*") pneumonia
		Pneumonia, recurrent
		Progressive multifocal leukoencephalopathy
		Salmonella septicemia, recurrent

		Toxoplasmosis of brain, onset at age >1 month
		Wasting syndrome attributed to HIV
